# Non-canonical function of DGCR8 in DNA double-strand break repair signaling and tumor radioresistance

**DOI:** 10.1038/s41467-021-24298-z

**Published:** 2021-06-29

**Authors:** Qinglei Hang, Liyong Zeng, Li Wang, Litong Nie, Fan Yao, Hongqi Teng, Yalan Deng, Shannon Yap, Yutong Sun, Steven J. Frank, Junjie Chen, Li Ma

**Affiliations:** 1 Department of Experimental Radiation Oncology, The University of Texas MD Anderson Cancer Center, Houston, TX USA; 2 Department of Radiation Oncology, The University of Texas MD Anderson Cancer Center, Houston, TX USA; 3 Department of Molecular and Cellular Oncology, The University of Texas MD Anderson Cancer Center, Houston, TX USA; 4 The University of Texas MD Anderson UTHealth Graduate School of Biomedical Sciences, Houston, TX USA; 5 Present Address: College of Biomedicine and Health, College of Life Science and Technology, Huazhong Agricultural University, Wuhan, Hubei China

**Keywords:** Radiotherapy, Ubiquitylation, Double-strand DNA breaks

## Abstract

In response to DNA double-strand breaks (DSBs), repair proteins are recruited to the damaged sites. Ubiquitin signaling plays a critical role in coordinating protein recruitment during the DNA damage response. Here, we find that the microRNA biogenesis factor DGCR8 promotes tumor resistance to X-ray radiation independently of its Drosha-binding ability. Upon radiation, the kinase ATM and the deubiquitinase USP51 mediate the activation and stabilization of DGCR8 through phosphorylation and deubiquitination. Specifically, radiation-induced ATM-dependent phosphorylation of DGCR8 at serine 677 facilitates USP51 to bind, deubiquitinate, and stabilize DGCR8, which leads to the recruitment of DGCR8 and DGCR8’s binding partner RNF168 to MDC1 and RNF8 at DSBs. This, in turn, promotes ubiquitination of histone H2A, repair of DSBs, and radioresistance. Altogether, these findings reveal the non-canonical function of DGCR8 in DSB repair and suggest that radiation treatment may result in therapy-induced tumor radioresistance through ATM- and USP51-mediated activation and upregulation of DGCR8.

## Abstract (web summary)

The molecular mechanisms underlying cancer cell radioresistance need to be elucidated. In this study, the authors show that the microRNA biogenesis factor DGCR8 is stabilized by USP51 and ATM upon irradiation and by consequence it promotes the repair of DNA double-strand breaks and radioresistance by recruiting RNF168 to sites of damage.

## Introduction

Radiotherapy, the use of high-energy particles or waves such as X-rays to cure or shrink tumors, has been a common treatment for many patients with cancer^1^. Although both normal and cancerous cells are damaged by radiation, the goal of radiation therapy is to selectively destroy cancer cells, since normal cells can often repair much of the damage caused by radiotherapy^2^. Unfortunately, some tumors exhibit either intrinsic or therapy-induced radioresistance, and effective and safe radiosensitizers are still lacking^3–6^.

DNA double-strand breaks (DSBs), which are generated by endogenous agents and exogenous insults including ionizing radiation (IR), are highly cytotoxic cellular lesions that, if not properly repaired, can cause genomic instability, cell-cycle arrest, and cell death^7–10^. To maintain genome stability, cells have evolved a complex system called the DNA damage response (DDR) system, and radioresistant tumor cells often exhibit increased DDR and DNA repair ability^3,10^. In the DDR system, genomic DSBs are sensed by the MRE11-RAD50-NBS1 complex, which recruits the kinase ATM to the vicinity of DNA lesions. The resulting phosphorylation of the histone variant H2AX by ATM allows the accumulation of MDC1 protein and its binding partners^9,11–13^. As the early event in a ubiquitination cascade, phosphorylated MDC1 recruits RNF8, a RING-type ubiquitin ligase that initiates mono-ubiquitination and K63-linked poly-ubiquitination of H2A type histones at sites of DNA damage^14–17^. Subsequently, another ubiquitin ligase, RNF168, is recruited to the chromatin surrounding DNA damage sites to augment the ubiquitination of histone H2A/H2AX. An alternative model, however, proposes that RNF168 initiates H2A/H2AX ubiquitination on K13-15, while RNF8 extends the histone ubiquitination by adding K63-linked ubiquitin chains^18^. Ultimately, H2A/H2AX ubiquitination triggers a second wave of protein accumulation, including DNA repair proteins BRCA1 and 53BP1^14–17,19–23^. Ubiquitination involved in DDR is subject to regulation at several levels, and dysregulated histone ubiquitination leads to aberrant transcriptional silencing, defects in DNA damage checkpoint activation and cell-cycle arrest, and genomic instability^24^. In addition to the ubiquitin ligases, several deubiquitinating enzymes (DUBs), such as OTUB1, USP3, USP16, USP44, and USP51, are also involved in DDR^25–31^. Altogether, these studies underscore the importance of coordinated ubiquitination at DNA damage sites during DDR. Despite these advances, how cells precisely orchestrate the ubiquitination and deubiquitination events and recruit the DNA repair proteins to the DNA lesions has not been fully unraveled.

MicroRNAs (miRNAs) are endogenously expressed small non-coding RNAs that regulate gene expression post-transcriptionally^32^. In the nucleus, the microprocessor complex consisting of the RNase Drosha and its binding partner DGCR8 cleaves the primary transcripts of miRNAs (pri-miRNAs) to generate miRNA precursors (pre-miRNAs). The pre-miRNAs are then exported by Exportin-5 to the cytoplasm, where they are cleaved by another RNase, Dicer, to generate mature miRNAs^33^. We and others have demonstrated that certain miRNAs participate in DDR and radiation response^34–40^. Moreover, the miRNA biogenesis machinery has been implicated in DNA repair outside of canonical miRNA-mediated target mRNA cleavage and translational inhibition. For example, Dicer and Drosha can generate dsRNAs that promote DNA repair by facilitating the recruitment of DDR factors^41–43^ or through the formation of DNA:RNA hybrids around DNA break sites^44^. Moreover, both Dicer and DGCR8 have been reported to regulate nucleotide excision repair of ultraviolet-induced lesions (pyrimidine dimers and base modifications)^45,46^. However, the precise function of miRNA biogenesis factors in DSB repair and radiotherapy response remains unknown. In this study, we uncovered a non-canonical function of DGCR8 in DSB repair signaling and tumor radioresistance. In response to IR, the kinase ATM and the deubiquitinase USP51 mediate the activation and stabilization of DGCR8 through phosphorylation and deubiquitination. Once phosphorylated, DGCR8 and its constitutive binding partner RNF168 in turn interact with MDC1 and RNF8 to promote histone ubiquitination, DSB repair, and radioresistance.

## Results

### DGCR8 promotes tumor radioresistance independently of Drosha binding

**Fig. 1:**
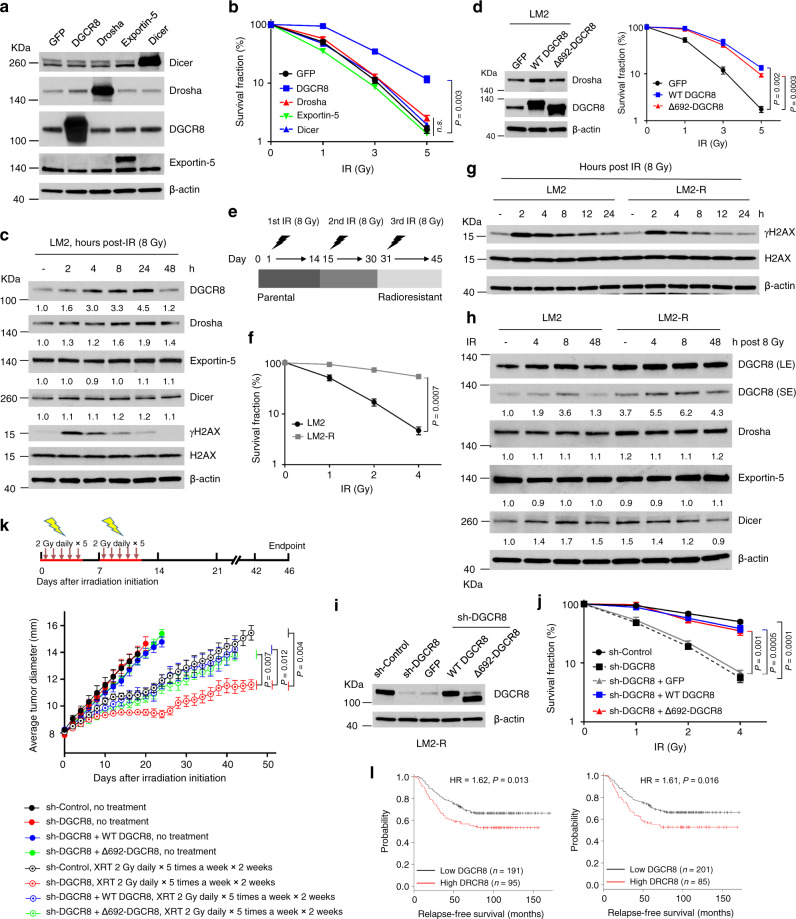
DGCR8 promotes tumor radioresistance independently of Drosha binding. **a** Immunoblotting of Dicer, Drosha, DGCR8, Exportin-5, and β-actin in LM2 cells transduced with GFP, Drosha, DGCR8, Exportin-5, or Dicer. **b** Clonogenic survival assays of LM2 cells transduced with GFP, Drosha, DGCR8, Exportin-5, or Dicer after X-ray ionizing radiation (IR) treatment. *n* = 3 wells per group. **c** Immunoblotting of DGCR8, Drosha, Exportin-5, Dicer, γH2AX, H2AX, and β-actin in LM2 cells collected at the indicated times after X-ray IR treatment. Quantification results were normalized to β-actin. **d** Immunoblotting of Drosha, DGCR8, and β-actin (left panel) and clonogenic survival assays (right panel) of LM2 stable cell lines overexpressing GFP, wild-type (WT) DGCR8, or Δ692-DGCR8 (the Drosha binding-deficient mutant). *n* = 3 wells per group. **e** Schematic representation of the generation of radioresistant sublines (LM2-R and MCF-7-R) from parental LM2 and MCF-7 breast cancer cell lines by three rounds of X-ray irradiation. **f** Clonogenic survival assays of parental LM2 and LM2-R cells after X-ray IR treatment. *n* = 3 wells per group. **g** Immunoblotting of γH2AX, H2AX, and β-actin in parental LM2 and LM2-R cells collected at the indicated times after IR. **h** Immunoblotting of DGCR8, Drosha, Dicer, Exportin-5, and β-actin in LM2 and LM2-R cells collected at the indicated times after IR. LE long exposure, SE short exposure. Quantification results were normalized to β-actin. **i**, **j** Immunoblotting of DGCR8 and β-actin (**i**) and clonogenic survival assays (**j**) of control or DGCR8-knockdown LM2-R cells transduced with GFP, WT DGCR8, or Δ692-DGCR8. *n* = 3 wells per group. **k** Tumor size of mice bearing xenograft tumors formed by control or DGCR8-knockdown LM2-R cells transduced with WT DGCR8 or Δ692-DGCR8, with or without fractionated doses of localized X-ray IR treatment (XRT) using an X-RAD 320 irradiator. *n* = 5 mice per group. **l** Kaplan–Meier curves of relapse-free survival of breast cancer patients (dataset: GSE2034; *n* = 286 patients, 87% of whom received radiotherapy), stratified by *DGCR8* expression levels. Data were generated from the KM Plotter (probes: 64474_g_at in the left panel and 91617_at in the right panel). The auto-select best cutoff was used in the analysis. Statistical significance was determined by a log-rank test. HR hazard ratio. Statistical significance in **b**, **d**, **f**, **j**, and **k** was determined by a two-tailed unpaired *t*-test. Error bars are mean ± SEM. Source data are provided as a Source Data file.

To examine the association between the key miRNA biogenesis proteins and radioresistance, we stably expressed Drosha, DGCR8, Exportin-5, or Dicer in LM2 cells (Fig. 1a), a lung-metastatic subline of MDA-MB-231 human breast cancer cells^47^. Only DGCR8, but not Drosha, Exportin-5, or Dicer, increased clonogenic survival after X-ray IR (Fig. 1b). Moreover, among these four proteins, only DGCR8 was substantially upregulated upon IR treatment in a time-dependent manner (Fig. 1c).

Next, we compared the effects of wild-type (WT) DGCR8 and the Drosha binding-deficient mutant Δ692-DGCR8, which lacks the Drosha-binding domain and RNA processing ability^48^, on clonogenic survival of several cell lines after X-ray radiation treatment. Overexpression of the Δ692-DGCR8 mutant in LM2, T47D, BT549, HCT116, HepG2, and HeLa cell lines promoted radioresistance just like WT DGCR8 (Fig. 1d and Supplementary Fig. 1a). Conversely, shRNA-mediated knockdown of DGCR8 in LM2, MCF-7, and BT549 cell lines sensitized the cells to X-ray treatment (Supplementary Fig. 1b); this radiosensitizing effect could be reversed by ectopic expression of either WT DGCR8 or Δ692-DGCR8 (Supplementary Fig. 1c, d). Taken together, these results suggest that DGCR8 positively regulates cancer cell radioresistance independently of its Drosha-binding ability.

To determine whether DGCR8 is upregulated in radioresistant tumor cells, we used X-ray IR to select radioresistant subpopulations from LM2 and MCF-7 cell lines. After an 8-Gray (Gy) dose, surviving cells formed colonies. We pooled the colonies and repeated the dose twice (Fig. 1e). Cells derived from this selection, named LM2-R and MCF-7-R cells, showed increased clonogenic survival upon IR relative to the parental cells (Fig. 1f and Supplementary Fig. 1e). We also compared levels of γH2AX, a marker of DSBs, based on the knowledge that IR-induced DSBs result in the formation of γH2AX foci, and that the persistence of γH2AX foci indicates delayed repair and is associated with radiosensitivity^49–51^. Notably, 12 h after irradiation, γH2AX levels stayed high in parental LM2 cells but decreased substantially in LM2-R cells (Fig. 1g), indicating that the LM2-R subline is more able to clear DSBs. We then examined the protein levels of Drosha, DGCR8, Exportin-5, and Dicer. Not only was DGCR8 markedly upregulated in LM2-R cells compared with parental LM2 cells, but it also showed a substantial increase after IR treatment (Fig. 1h). Similarly, DGCR8 protein was also upregulated in MCF-7-R cells compared with parental MCF-7 cells (Supplementary Fig. 1e). Because DGCR8 was upregulated in the radioresistant cells, we silenced its expression, which led to radiosensitization of both LM2-R (Fig. 1i, j) and MCF-7-R (Supplementary Fig. 1e) cells. Importantly, the inhibited clonogenic survival ability upon IR could be rescued by ectopic expression of either WT DGCR8 or the Δ692-DGCR8 mutant (Fig. 1i, j).

We further validated our findings in mice bearing LM2-R xenograft tumors. When the tumor diameters reached 8 mm, we irradiated the tumors with a 2-Gy fractionated dose of X-ray once a day for 5 consecutive days a week, and the treatment lasted 2 weeks. As shown in Fig. 1k, knockdown of DGCR8, as well as restoration of DGCR8 expression, had no substantial effect on tumor growth without irradiation. In contrast, treatment with fractionated X-ray led to sustained growth inhibition of tumors formed by DGCR8-knockdown cells, whereas tumors formed by either control LM2-R cells or cells with re-expression of DGCR8 (either WT or the Δ692 mutant) showed an initial response and then re-grew (Fig. 1k). Collectively, these findings indicate that DGCR8 promotes the radioresistance of breast cancer cells in vitro and in vivo independently of Drosha binding.

To assess the clinical relevance of DGCR8 expression to radiotherapy in patients, we analyzed a cohort of human breast cancer patients in which transcriptomic profiling was obtained from 286 tumor samples; 87% of these patients had received radiotherapy^52^. This analysis revealed that patients with high DGCR8 expression levels in their tumors had worse relapse-free survival than those with low DGCR8 expression levels (Fig. 1l; with two different probes), suggesting that overexpression of DGCR8 may lead to radioresistance and eventually relapse in patients with breast cancer.

### DGCR8 promotes the recruitment of RNF168 to RNF8 and MDC1 at DSBs, histone ubiquitination, and DNA repair after irradiation

**Fig. 2:**
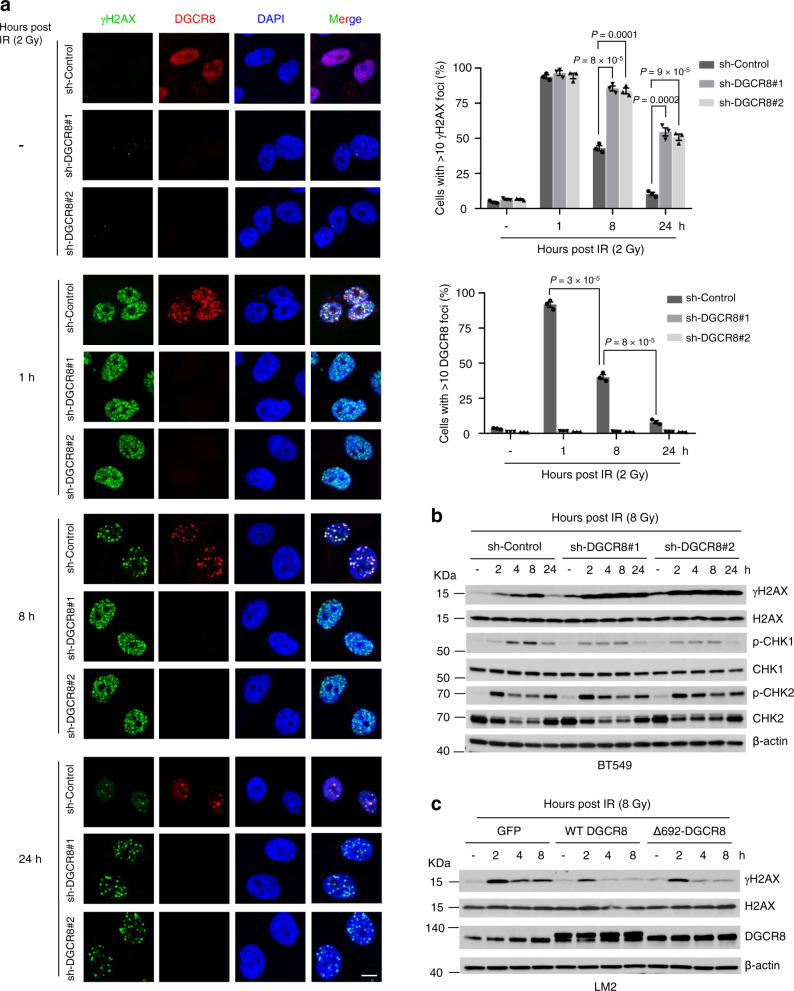
DGCR8 promotes the clearance of DNA breaks after irradiation. **a** Left panels: DGCR8-knockdown LM2 cells were treated with 2-Gy ionizing radiation (IR). After the indicated times, cells were collected for immunofluorescent staining of γH2AX (green), DGCR8 (red), and DAPI (blue). Scale bar, 10 μm. Right panels: quantification of γH2AX-positive and DGCR8-positive foci. *n* = 3 biological replicates. Statistical significance was determined by a two-tailed unpaired *t*-test. Error bars are mean ± SEM. **b** Immunoblotting of γH2AX, H2AX, p-CHK1, CHK1, p-CHK2, CHK2, and β-actin in DGCR8-knockdown BT549 cells collected at the indicated times after X-ray IR treatment. **c** Immunoblotting of γH2AX, H2AX, DGCR8, and β-actin in GFP-, WT DGCR8-, and Δ692-DGCR8-overexpressing LM2 cells collected at the indicated times after X-ray IR treatment. Source data are provided as a Source Data file.

**Fig. 3:**
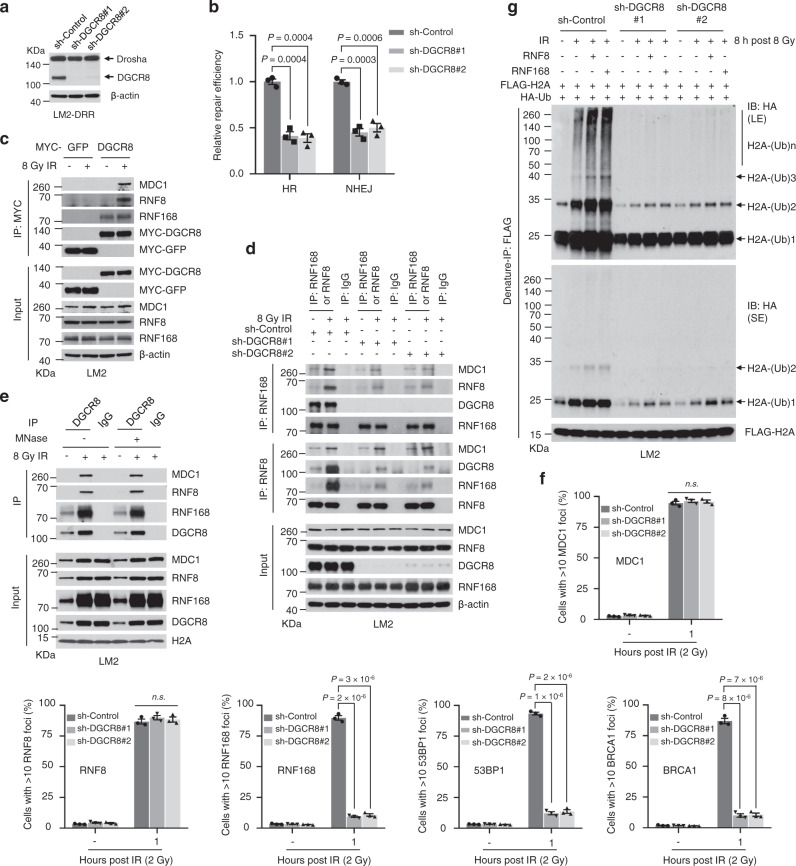
DGCR8 promotes the recruitment of RNF168 to RNF8 and MDC1 at DSBs, histone ubiquitination, and DNA repair upon irradiation. **a** Immunoblotting of Drosha, DGCR8, and β-actin in the LM2-DRR (expressing the pLCN DSB Repair Reporter) cell line transduced with DGCR8 shRNA. **b** Knockdown of DGCR8 decreased HR and NHEJ efficiency in LM2-DRR cells. Two days after co-transfection of I-SceI endonuclease and an exogenous donor for HR (pCAGGS DRR mCherry Donor EF1a BFP) into the DGCR8-knockdown LM2-DRR cells, the percentages of GFP-positive and mCherry-positive cells, gated on BFP-positive cells, were determined by flow cytometry. Repair by HR or NHEJ leads to mCherry or GFP expression. Data were normalized to the control cells. *n* = 3 biological replicates. **c** MYC-DGCR8-overexpressing LM2 cells were treated with IR (8 Gy) and cultured for 1 h, followed by pulldown with MYC beads and immunoblotting with the indicated antibodies. **d** Control and DGCR8-knockdown LM2 cells were treated with IR (8 Gy) and cultured for 1 h, followed by immunoprecipitation with an antibody against RNF168 or RNF8 and immunoblotting with the indicated antibodies. **e** Chromatin was extracted from LM2 cells that were treated with IR (8 Gy) and cultured for 1 h. The chromatin fractions, with or without MNase treatment, were immunoprecipitated with a DGCR8-specific antibody and immunoblotted with the indicated antibodies. **f** Quantification of MDC1, RNF8, RNF168, 53BP1, and BRCA1 foci in DGCR8-knockdown LM2 cells. Cells were incubated for 1 h after 2-Gy IR and immunostained with antibodies against γH2AX, MDC1, RNF8, RNF168, 53BP1, and BRCA1 (see representative images in Supplementary Fig. 3). *n* = 3 biological replicates. **g** Control and DGCR8-knockdown LM2 cells with stable overexpression of FLAG-H2A and RNF8 or RNF168 were transfected with HA-ubiquitin (Ub), treated with IR (8 Gy), and cultured for 8 h, followed by immunoprecipitation with anti-FLAG beads and immunoblotting with antibodies against HA and FLAG. Before immunoprecipitation, lysates were heated at 95 °C for 5 min in the presence of 1% SDS (for denaturing), followed by a 10-fold dilution with lysis buffer and sonication. LE long exposure, SE short exposure. Statistical significance in **b** and **f** was determined by a two-tailed unpaired *t*-test. Error bars are mean ± SEM. *n.s*. not statistically significant. Source data are provided as a Source Data file.

To understand the molecular basis of DGCR8-mediated radioresistance, we first examined γH2AX foci at different time points after X-ray IR, and we observed persistent γH2AX signals after irradiation in DGCR8-knockdown LM2 and BT549 cells, but not in the control cells (Fig. 2a, b). Importantly, DGCR8 was localized to IR-induced foci that overlapped with γH2AX-positive foci in a time-dependent manner (Fig. 2a). Conversely, overexpression of either WT DGCR8 or Δ692-DGCR8 in parental LM2 cells accelerated the clearance of γH2AX after irradiation (Fig. 2c), suggesting that DGCR8 may empower cells to repair DSBs in an RNA processing-independent manner. Non-homologous end-joining (NHEJ) and homologous recombination (HR) are two distinct DSB repair pathways in eukaryotic cells^53,54^. We used a previously established fluorescence-based reporter system that allows quantitative analysis of NHEJ and HR in the same cells through the repair of two inverted endonuclease I-SceI cuts^55^; by constructing a reporter cell line (LM2-DRR), we found that depletion of DGCR8 significantly decreased HR and NHEJ repair efficiency (Fig. 3a, b and Supplementary Fig. 2a).

To further investigate the molecular mechanism by which DGCR8 regulates DNA repair, we attempted to identify DGCR8-interacting DDR proteins with or without IR treatment of LM2 cells by using a triple epitope (S-protein, FLAG tag, and streptavidin-binding peptide)-tagged version of DGCR8 (SFB-DGCR8). We performed tandem-affinity purification with streptavidin-sepharose beads and S-protein agarose beads, followed by mass spectrometric analysis, which identified several DGCR8-interacting DDR proteins in both non-irradiated and irradiated LM2 cells; among these DDR proteins, RNF168 was ranked the top one according to the intensity-based absolute quantitation (iBAQ) value (Supplementary Fig. 2b). On the other hand, another two DDR proteins, MDC1 and RNF8, were the top two DGCR8-interacting DDR proteins identified only in irradiated cells (Supplementary Fig. 2b). The cellular response to DSBs involves activation of ATM and subsequent production of γH2AX, which binds and recruits MDC1 to DNA damage sites^56,57^. Then, the ubiquitin ligases RNF8 and RNF168 are recruited in an MDC1-dependent manner^20,21,58^. Consistent with our mass spectrometry results, co-immunoprecipitation (Co-IP) assays showed that IR treatment induced the interaction of DGCR8 with MDC1 and RNF8 in both LM2 and HEK293T cells, whereas the DGCR8-RNF168 interaction was constitutive (Fig. 3c and Supplementary Fig. 2c). By performing glutathione S transferase (GST) pulldown assays under cell-free conditions, we detected interaction between purified DGCR8 and purified RNF168 (Supplementary Fig. 2d), suggesting that RNF168 is DGCR8’s direct and constitutive binding partner. Importantly, by performing Co-IP experiments to examine the interactions between endogenous proteins, we found that knockdown of DGCR8 in LM2 cells blocked IR-induced RNF168-RNF8 and RNF168-MDC1 interactions, without inhibiting the RNF8-MDC1 interaction (Fig. 3d), which suggests that DGCR8 is required for recruiting RNF168 to MDC1 and RNF8 after irradiation. Consistent with the DGCR8-knockdown effect (Fig. 3a, b), knockdown of RNF168 also reduced HR and NHEJ repair efficiency in LM2-DRR cells (Supplementary Fig. 2e–g).

Next, we performed chromatin extraction followed by IP, with or without MNase treatment. As shown in Fig. 3e, the recruitment of DGCR8, MDC1, RNF8, and RNF168 to chromatin was induced by irradiation, and the interactions of DGCR8 with RNF168, RNF8, and MDC1 were detected in chromatin extracts. Interestingly, similar interaction patterns were observed with or without MNase treatment (Fig. 3e). These results indicate that the interactions of DGCR8 with RNF168, RNF8, and MDC1 occur on chromatin, but these protein–protein interactions are not mediated by chromatin. Importantly, immunofluorescent staining of irradiated LM2 cells showed that knockdown of DGCR8 did not affect the recruitment of MDC1 or RNF8 to DSBs, but abolished the localization of RNF168, 53BP1, and BRCA1 to DNA damage sites (Fig. 3f and Supplementary Fig. 3). Given that RNF8 and RNF168 ubiquitinate H2A to drive DDR^16,18,20^, we determined the effect of DGCR8 on RNF8- and RNF168-induced ubiquitination of H2A. Upon IR, both RNF8 and RNF168 could enhance mono-, di-, and poly-ubiquitination of H2A, which was blocked by knockdown of DGCR8 (Fig. 3g). Taken together, these results suggest that after radiation treatment, DGCR8 mediates the recruitment of its binding partner RNF168 to MDC1 and RNF8 at DSBs, leading to amplification of histone ubiquitination.

### Radiation-induced USP51 mediates deubiquitination and stabilization of DGCR8

**Fig. 4:**
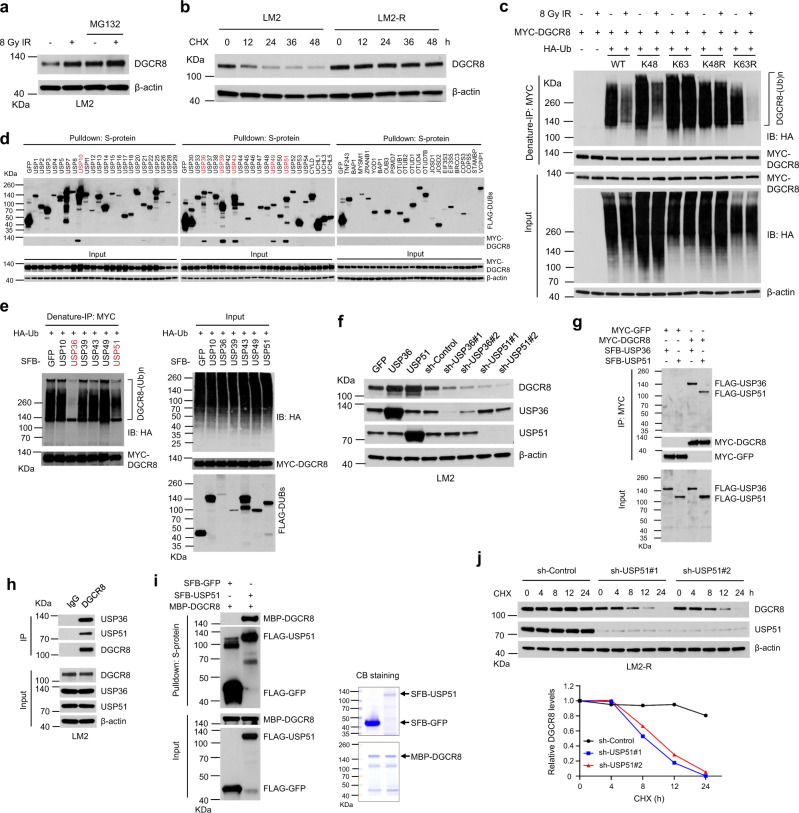
USP51 binds and stabilizes DGCR8. **a** LM2 cells were treated with 10 μM MG132, irradiated with 8 Gy X-ray, and collected 6 h later. Lysates were immunoblotted with antibodies against DGCR8 and β-actin. **b** Parental and radioresistant LM2 cells were treated with 100 μg/ml cycloheximide (CHX). Cells were collected at different time points and immunoblotted with antibodies against DGCR8 and β-actin. **c** HEK293T cells were co-transfected with MYC-DGCR8 and HA-ubiquitin (Ub) or its lysine-specific mutants (K48, K63, K48R, or K63R), followed by immunoprecipitation with anti-MYC beads and immunoblotting with antibodies against HA and MYC. Cells were treated with IR (8 Gy), followed by treatment with 10 μM MG132 for 6 h. Before immunoprecipitation, lysates were heated at 95 °C for 5 min in the presence of 1% SDS (for denaturing), followed by a 10-fold dilution with lysis buffer and sonication. **d** SFB-tagged DUBs were individually co-transfected with MYC-DGCR8 into HEK293T cells, followed by pulldown with S-protein beads and immunoblotting with antibodies against FLAG and MYC. **e** HEK293T cells were co-transfected with MYC-DGCR8, HA-ubiquitin, and a candidate DUB. After treatment with MG132 for 6 h, cells were lysed, denatured, and subjected to immunoprecipitation with anti-MYC beads and immunoblotting with antibodies against HA and MYC. **f** Immunoblotting of DGCR8, USP36, USP51, and β-actin in LM2 cells with overexpression or knockdown of USP36 or USP51. **g** HEK293T cells were co-transfected with MYC-DGCR8 and SFB-tagged USP36 or USP51. After 48 h, cells were lysed, immunoprecipitated with anti-MYC beads, and immunoblotted with antibodies against FLAG and MYC. **h** Co-IP of endogenous DGCR8 with endogenous USP36 and USP51. DGCR8 was immunoprecipitated from LM2 cells and immunoblotted with antibodies against USP36, USP51, and DGCR8. **i** USP51 binds DGCR8 in vitro. Left panel: SFB-GFP or SFB-USP51 was retained on S-protein beads and incubated with purified MBP-DGCR8. The bound proteins were eluted by boiling in Laemmli buffer and immunoblotted with antibodies against MBP and FLAG. Right panel: purified SFB-GFP, SFB-USP51, and MBP-DGCR8 proteins were analyzed by SDS-PAGE and Coomassie blue (CB) staining. **j** Upper panel: LM2-R cells stably infected with USP51 shRNA were treated with 100 μg/ml CHX for the indicated times. Lysates were subjected to immunoblotting with antibodies against DGCR8, USP51, and β-actin. Lower panel: DGCR8 levels were quantitated and normalized to β-actin. Source data are provided as a Source Data file.

We next sought to understand how radiation leads to the upregulation of DGCR8 protein. Although X-ray IR had little or no effect on *DGCR8* mRNA levels (Supplementary Fig. 4a), irradiation of both LM2 and MCF-7 cells markedly upregulated DGCR8 protein to levels comparable to treatment with the proteasome inhibitor MG132 (Fig. 4a and Supplementary Fig. 4b). Moreover, we examined DGCR8 levels in the presence of the protein synthesis inhibitor cycloheximide (CHX) and found that compared with parental LM2 and MCF-7 cells, DGCR8 stability was much higher in the radioresistant sublines derived from X-ray irradiation (Fig. 4b and Supplementary Fig. 4c, d). We then examined proteolytic (K48-linked) and non-proteolytic (K63-linked) ubiquitination levels of DGCR8. By using lysine mutants of ubiquitin (K48, K63, K48R, and K63R), we observed a substantial decrease in total and K48-linked ubiquitination of DGCR8 24 h after X-ray IR (Fig. 4c). These results suggest that radiation may stabilize DGCR8 protein by reducing its proteolytic ubiquitination.

DUBs are proteases that remove mono-ubiquitin or poly-ubiquitin chains from substrate proteins^59^. We screened for DGCR8-interacting DUBs by a pulldown assay using a panel of 68 SFB-tagged human DUBs^60^. We co-transfected MYC-DGCR8 and each SFB-tagged DUB into HEK293T cells, pulled down the DUBs with S-protein beads, and detected interaction of DGCR8 with six DUBs: USP10, USP36, USP39, USP43, USP49, and USP51 (Fig. 4d), which was verified by a confirmatory pulldown assay (Supplementary Fig. 4e). To determine the effect of these DUBs on DGCR8 ubiquitination, we co-expressed each SFB-tagged DUB with MYC-tagged DGCR8 and HA-tagged ubiquitin in HEK293T cells, and immunoprecipitation assays under denaturing conditions revealed that ectopic expression of either USP36 or USP51 reduced the poly-ubiquitination of DGCR8 (Fig. 4e). Through overexpression and knockdown of USP36 or USP51, we observed that both DUBs positively regulated the levels of endogenous DGCR8 protein in LM2 (Fig. 4f) and HEK293T (Supplementary Fig. 4f) cells. Moreover, Co-IP assays confirmed that both USP36 and USP51 could be pulled down by DGCR8 (Fig. 4g), and that both DUBs interacted with DGCR8 at endogenous levels in LM2 cells (Fig. 4h).

We then examined the subcellular localization of USP36, USP51, and DGCR8. Consistent with previous reports^31,48,61,62^, we detected USP36 specifically in the nucleolus, whereas both USP51 and DGCR8 proteins were localized diffusely in the nucleus (Supplementary Fig. 4g, h). To validate the direct interaction between USP51 and DGCR8, we expressed and purified SFB-USP51 and MBP-DGCR8 proteins, and in vitro pulldown assays demonstrated that USP51 bound to DGCR8 (Fig. 4i), indicating that DGCR8 may be directly regulated by USP51. To determine whether USP51 regulates the stability of DGCR8, we performed CHX treatment of the radioresistant LM2 (LM2-R) cells, finding that DGCR8 was less stable upon knockdown of USP51 (Fig. 4j).

To determine the deubiquitinating ability of USP51 towards DGCR8, we generated the catalytically inactive mutant of USP51, C372S^63^. Overexpression of WT USP51, but not the C372S mutant, removed K48-linked but not K63-linked poly-ubiquitin chains of DGCR8 in HEK293T cells (Supplementary Fig. 4i). To determine whether USP51 directly deubiquitinates DGCR8, we incubated purified USP51 and ubiquitinated DGCR8 purified from HEK293T cells in a cell-free system. Purified USP51, but not the C372S mutant, strongly deubiquitinated DGCR8 in vitro (Supplementary Fig. 4j), suggesting that DGCR8 is a substrate of USP51.

**Fig. 5:**
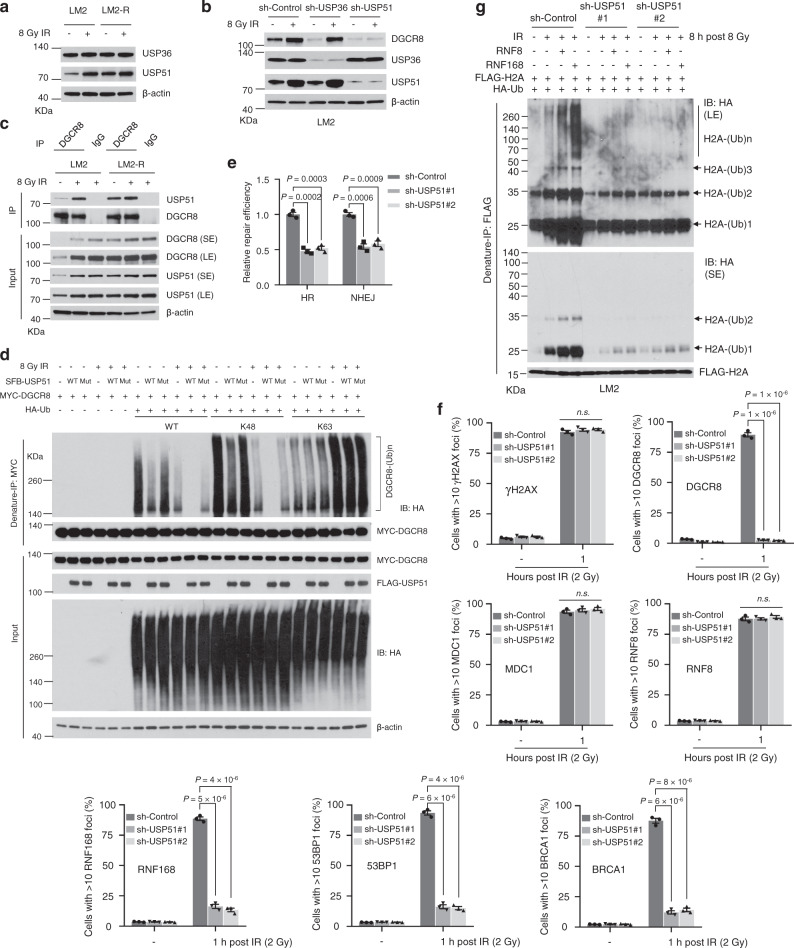
Radiation-induced USP51 mediates deubiquitination of DGCR8, histone ubiquitination, recruitment of DDR proteins to DSBs, and DNA repair. **a** Immunoblotting of USP36, USP51, and β-actin in parental and radioresistant LM2 cells with and without IR treatment (8 Gy followed by 24-h incubation). **b** Immunoblotting of DGCR8, USP36, USP51, and β-actin in USP36-knockdown and USP51-knockdown LM2 cells with or without IR treatment (8 Gy followed by 24-h incubation). **c** Co-IP of endogenous DGCR8 with endogenous USP51. LM2 and LM2-R cells were treated with 8-Gy IR. After 8 h, cells were lysed, immunoprecipitated with a DGCR8-specific antibody, and immunoblotted with antibodies against USP51 and DGCR8. SE short exposure, LE long exposure. **d** HEK293T cells with stable overexpression of MYC-DGCR8 were co-transfected with SFB-USP51 (wild-type or the C372S mutant) and HA-tagged ubiquitin or the lysine-specific mutant (K48 or K63), and then treated with IR (8 Gy). After 8 h, cells were lysed, denatured, and subjected to immunoprecipitation with anti-MYC beads and immunoblotting with antibodies against HA and MYC. **e** Knockdown of USP51 decreased HR and NHEJ efficiency in LM2-DRR cells. Two days after co-transfection of I-SceI endonuclease and an exogenous donor for HR (pCAGGS DRR mCherry Donor EF1a BFP) into the USP51-knockdown LM2-DRR cells, the percentages of GFP-positive and mCherry-positive cells, gated on BFP-positive cells, were determined by flow cytometry. Repair by HR or NHEJ leads to mCherry or GFP expression. Data were normalized to the control cells. *n* = 3 biological replicates. **f** Quantification of γH2AX, DGCR8, MDC1, RNF8, RNF168, 53BP1, and BRCA1 foci in USP51-knockdown LM2 cells. Cells were incubated for 1 h after 2-Gy IR and immunostained with antibodies against γH2AX, DGCR8, MDC1, RNF8, RNF168, 53BP1, and BRCA1 (see representative images in Supplementary Fig. 6). *n* = 3 biological replicates. **g** Control and USP51-knockdown LM2 cells with stable overexpression of FLAG-H2A and RNF8 or RNF168 were transfected with HA-ubiquitin (Ub), treated with IR (8 Gy), and cultured for 8 h, followed by immunoprecipitation with anti-FLAG beads and immunoblotting with antibodies against HA and FLAG. Before immunoprecipitation, lysates were heated at 95 °C for 5 min in the presence of 1% SDS (for denaturing), followed by a 10-fold dilution with lysis buffer and sonication. Statistical significance in **e** and **f** was determined by a two-tailed unpaired *t*-test. Error bars are mean ± SEM. Source data are provided as a Source Data file.

Interestingly, USP51, but not USP36, was upregulated upon X-ray radiation (Fig. 5a). In both LM2 and MCF-7 cells, IR-induced upregulation of DGCR8 was reversed by shRNA-mediated knockdown of USP51, but not by knockdown of USP36 (Fig. 5b and Supplementary Fig. 5a). Moreover, the interaction between DGCR8 and USP51 was enhanced in a time-dependent manner after irradiation of LM2 cells (Supplementary Fig. 5b), and this increased interaction was observed with endogenous proteins (Fig. 5c). In addition, the K48 linkage-specific deubiquitination of DGCR8 by USP51 was enhanced by IR treatment (Fig. 5d). Altogether, these data suggest that radiation-induced USP51 mediates deubiquitination and stabilization of DGCR8.

Similar to the DGCR8-knockdown effects (Fig. 3a, b, f), knockdown of USP51 not only decreased HR and NHEJ repair efficiency in LM2-DRR cells (Fig. 5e and Supplementary Fig. 5c, d), but also abrogated the recruitment of RNF168, 53BP1, and BRCA1 to DSBs after radiation treatment, without affecting the recruitment of MDC1 and RNF8 (Fig. 5f and Supplementary Fig. 6). Moreover, the mono-, di-, and poly-ubiquitination of H2A induced by RNF8 and RNF168 upon IR was abolished by USP51 knockdown (Fig. 5g). Therefore, USP51 is required for the recruitment of the DGCR8-RNF168 complex to DSBs, histone ubiquitination, and DNA repair after irradiation.

To validate the association between DGCR8 and USP51 in patients with breast cancer, we immunohistochemically stained these two proteins in breast tumors (*n* = 139) from patients with a long-term (~12 years) follow-up record (Supplementary Fig. 7a). A positive correlation between USP51 and DGCR8 was observed in these breast carcinomas, in which 73% (61 of 83) of the tumors with moderate or weak DGCR8 expression exhibited moderate or weak USP51 expression, and 75% (42 of 56) of the tumors with strong DGCR8 expression showed strong USP51 expression (Spearman correlation *R* = 0.48, *P* = 3 × 10^−9^; Supplementary Fig. 7a, b). We also plotted the USP51 protein score versus the DGCR8 protein score for individual patients, which revealed a highly significant correlation (Pearson *R*^2^ = 0.52, *P* < 1 × 10^−15^; Supplementary Fig. 7c), indicating the relevance of the identified regulatory mechanism in human cancer. Furthermore, Kaplan–Meier analysis showed that high levels of DGCR8 (log-rank *P* = 2 × 10^−10^) and USP51 (log-rank *P* = 0.0001) were significantly associated with poor overall survival after surgery in patients with breast cancer (Supplementary Fig. 7d, e).

### ATM-mediated phosphorylation of DGCR8 promotes its upregulation and function upon irradiation

**Fig. 6:**
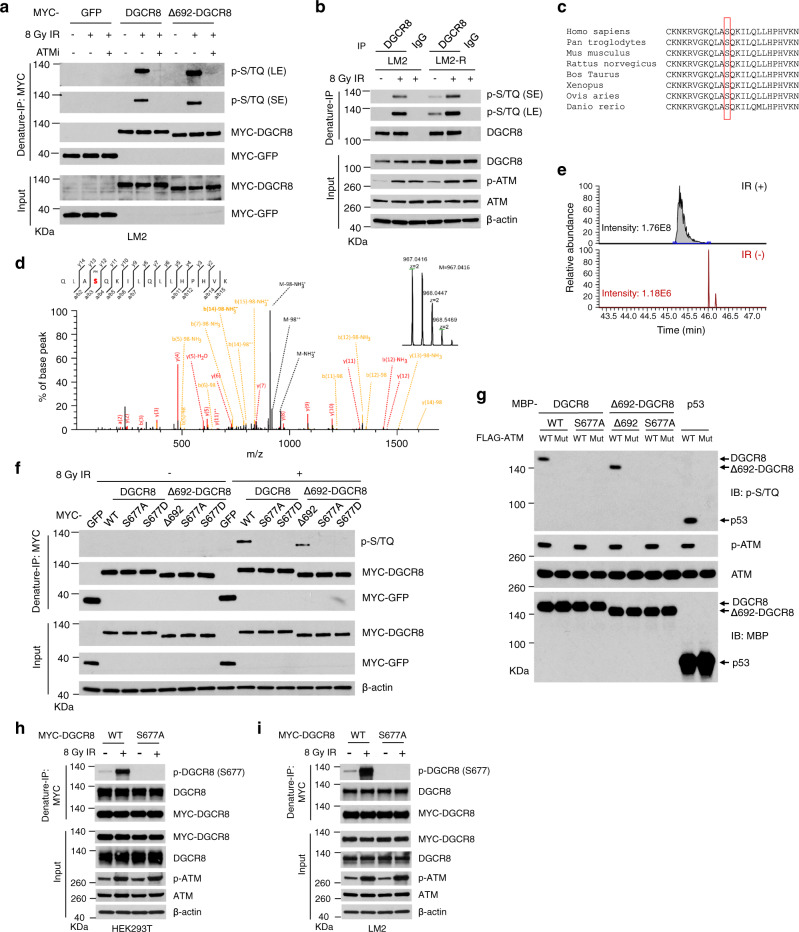
DGCR8 is phosphorylated by ATM at S677 upon radiation. **a** MYC-GFP-, DGCR8-, and Δ692-DGCR8-overexpressing LM2 cells with or without ATM inhibitor (ATMi) Ku55933 pretreatment (10 μM, 1 h) were treated with IR (8 Gy) and cultured for 30 min, followed by pulldown with anti-MYC beads and immunoblotting with antibodies against p-S/TQ and MYC. LE long exposure, SE short exposure. **b** LM2 and LM2-R cells were treated with IR (8 Gy) and cultured for 30 min, followed by immunoprecipitation with a DGCR8-specific antibody and immunoblotting with antibodies against p-S/TQ and DGCR8. **c** Consensus ATM phosphorylation site on human DGCR8 (S677) and alignment with the conserved site on Dgcr8 from other species. **d** Annotated tandem mass spectrometry (MS/MS) spectrum of the peptide encompassing phosphorylated serine 677 (QLASphosQKILQLLHPHVK) of DGCR8. MYC-DGCR8-overexpressing LM2 cells with or without IR (8 Gy followed by 1-h incubation) were lysed, denatured, and subjected to immunoprecipitation with anti-MYC beads, followed by MS analysis. PH indicates phosphorylation. NH_3_, H_2_O, and ++ indicate ammonia, water, and double charges, respectively. The graph shows the mass-to-charge ratios (m/z) of the doubly charged precursor peptide ions. The *x* and *y* axes represent m/z and relative ion intensity, respectively. **e** The extracted ion chromatograms for S677phos (peptide QLAS(phos)QKILQLLHPHVK) in non-irradiated and irradiated LM2 cells based on high-performance liquid chromatography (HPLC)-MS/MS analysis. The *x* and *y* axes represent the retention time of HPLC/MS analysis and the MS intensity, respectively. The area under the curve is used to indicate the relative abundance of S677phos with or without IR treatment. **f** HEK293T cells were transfected with DGCR8 (full-length or Δ692), S677A, or S677D mutant, treated with IR (8 Gy), and cultured for 30 min, followed by immunoprecipitation with anti-MYC beads and immunoblotting with antibodies against p-S/TQ and MYC. **g** In vitro kinase assay. Purified WT DGCR8 and Δ692-DGCR8 or their S677A mutants were incubated with purified wild-type ATM or the kinase-dead mutant in kinase buffer. After the reaction, proteins were resolved by SDS-PAGE and subjected to immunoblotting with the indicated antibodies. Purified MBP-p53 was used as a positive control for ATM kinase activity. **h**, **i** HEK293T (**h**) and LM2 (**i**) cells were transfected with MYC-tagged WT DGCR8 or the S677A mutant, treated with IR (8 Gy), and cultured for 30 min, followed by immunoprecipitation with anti-MYC beads and immunoblotting with antibodies against p-DGCR8 (S677), DGCR8, and MYC.

We investigated whether and how DGCR8 protein is regulated by upstream signaling in DDR. By using a phospho-S/TQ (p-S/TQ) antibody, we found that either WT DGCR8 or Δ692-DGCR8 was phosphorylated at ATM/ATR consensus motifs in LM2 and HEK293T cells upon IR treatment (Fig. 6a and Supplementary Fig. 8a), and that an ATM kinase inhibitor, Ku55933, abolished radiation-induced S/TQ phosphorylation of DGCR8 (Fig. 6a and Supplementary Fig. 8a), suggesting that DGCR8 is phosphorylated in an ATM-dependent manner after radiation. We also detected the phospho-S/TQ signal of endogenous DGCR8 protein in LM2-R cells, which exhibited higher basal levels of ATM phosphorylation than parental LM2 cells, and the S/T-Q phosphorylation of endogenous DGCR8 was upregulated after IR treatment of both LM2 and LM2-R cells (Fig. 6b). Analysis of the DGCR8 protein sequence revealed one evolutionarily conserved ATM phosphorylation site, serine 677 (S677; Fig. 6c and Supplementary Fig. 8b). Importantly, mass spectrometric analysis provided direct evidence that radiation treatment induced the phosphorylation of DGCR8 at S677 (Fig. 6d, e). Consistently, substitution of S677 with alanine or aspartic acid (S677A or S677D) abrogated the S/T-Q phosphorylation of either WT DGCR8 or Δ692-DGCR8 in irradiated HEK293T cells, further validating that S677 is indeed the site of radiation-induced ATM-dependent phosphorylation (Fig. 6f). To determine whether DGCR8 is a substrate of ATM, we performed in vitro kinase assays with purified proteins, finding that ATM phosphorylated either full-length DGCR8 protein or Δ692-DGCR8, but not their S677A mutants (Fig. 6g). In addition, radiation-induced S677 phosphorylation of DGCR8 was further validated by using an antibody against the phosphorylated S677 site of DGCR8 in both HEK293T (Fig. 6h) and LM2 (Fig. 6i) cells.

**Fig. 7:**
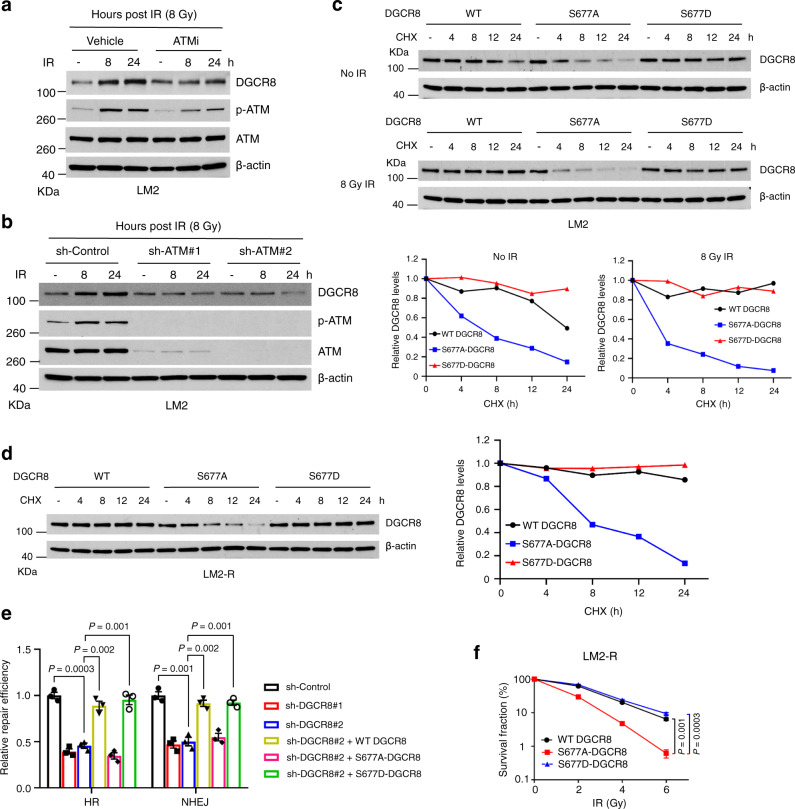
ATM-mediated S677 phosphorylation of DGCR8 is crucial for DGCR8 stability, DSB repair, and radiation response. **a** LM2 cells were pretreated with ATMi (Ku55933, 10 μM for 1 h), followed by IR treatment (8 Gy). Lysates were collected at the indicated times and immunoblotted with antibodies against DGCR8, p-ATM, ATM, and β-actin. **b** Immunoblotting of DGCR8, p-ATM, ATM, and β-actin in control and ATM-knockdown LM2 cells collected at the indicated times after IR. **c** Upper panels: DRCR8-knockdown LM2 cells with ectopic expression of WT DGCR8, the S677A mutant, or the S677D mutant were treated with CHX with or without IR, collected at different time points, and immunoblotted with antibodies against DGCR8 and β-actin. Lower panels: DGCR8 protein levels were quantitated and normalized to β-actin. **d** Left panel: DRCR8-knockdown LM2-R cells with ectopic expression of WT DGCR8, the S677A mutant, or the S677D mutant were treated with CHX, collected at different time points, and immunoblotted with antibodies against DGCR8 and β-actin. Right panel: DGCR8 protein levels were quantitated and normalized to β-actin. **e** The effect of S677 DGCR8 phosphorylation on HR and NHEJ efficiency in LM2-DRR cells. Two days after co-transfection of I-SceI endonuclease and an exogenous donor for HR (pCAGGS DRR mCherry Donor EF1a BFP) into DGCR8-knockdown and DGCR8-, S677A-DGCR8-, and S677D-DGCR8-rescued LM2-DRR cells, the percentages of GFP-positive and mCherry-positive cells, gated on BFP-positive cells, were determined by flow cytometry. Repair by HR or NHEJ leads to mCherry or GFP expression. Data were normalized to the control cells. *n* = 3 biological replicates. **f** Clonogenic survival assays of DRCR8-knockdown LM2-R cells with ectopic expression of WT DGCR8, the S677A mutant, or the S677D mutant after X-ray IR treatment. *n* = 3 wells per group. Statistical significance in **e** and **f** was determined by a two-tailed unpaired *t*-test. Error bars are mean ± SEM. Source data are provided as a Source Data file.

We asked whether ATM regulates DGCR8 by phosphorylating it at S677. Indeed, IR-induced upregulation of DGCR8 in LM2 cells was blocked by either the ATM inhibitor Ku55933 (Fig. 7a) or ATM shRNA (Fig. 7b), and similar effects were also observed in BT549 cells (Supplementary Fig. 8c, d). In contrast, neither treatment with the ATR inhibitor AZD6788 nor knockdown of ATR affected DGCR8 protein levels upon IR treatment (Supplementary Fig. 8e, f). Next, we stably expressed WT DGCR8, the phosphodeficient mutant (S677A), or the phosphomimetic mutant (S677D) in DGCR8-depleted LM2 and LM2-R cells (Supplementary Fig. 8g, h). Without irradiation of LM2 cells, the stability of WT DGCR8 was higher than that of the S677A mutant but lower than that of the S677D mutant (Fig. 7c); after irradiation of LM2 cells, the stability of WT DGCR8 was increased to a level as high as that of the S677D mutant, whereas the S677A mutant was much less stable (Fig. 7c). It should be noted that the S677A mutant of DGCR8 exhibited a shorter half-life than WT DGCR8 even without radiation treatment (Fig. 7c), which was likely due to the basal S677 phosphorylation of DGCR8 (Fig. 6h, i). In the radioresistant LM2-R cells, the stability of WT DGCR8 was much higher than that of the S677A mutant but was comparable to that of the S677D mutant (Fig. 7d). Therefore, ATM-dependent phosphorylation of DGCR8 at S677 is important for the IR-induced stabilization of DGCR8. Furthermore, we determined the HR and NHEJ repair efficiency in the DGCR8-depleted LM2-DRR reporter cell line with re-expression of WT DGCR8, the phosphodeficient mutant (S677A), or the phosphomimetic mutant (S677D), finding that the inhibited HR and NHEJ repair efficiency caused by DGCR8 knockdown was completely rescued by WT DGCR8 or the phosphomimetic mutant S677D, but not by the phosphodeficient mutant S677A (Fig. 7e and Supplementary Fig. 8i, j). In DGCR8-depleted LM2, LM2-R, and BT549 cells, the phosphodeficient mutant S677A was less able to promote radioresistance than WT DGCR8 or the phosphomimetic mutant S677D (Fig. 7f and Supplementary Fig. 8k, l), suggesting that ATM-mediated phosphorylation of DGCR8 is critical for its regulation of radiation response.

**Fig. 8:**
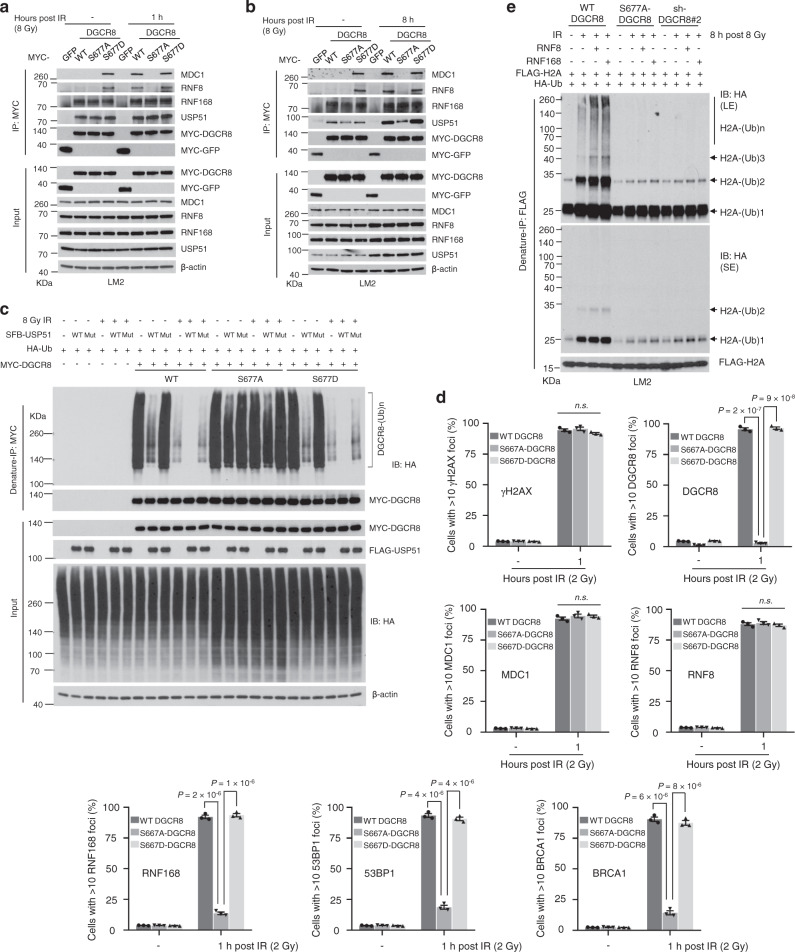
S677 DGCR8 phosphorylation is critical for the recruitment of RNF168 to RNF8 and MDC1, DGCR8-USP51 interaction, DGCR8 deubiquitination, and histone ubiquitination upon irradiation. **a**, **b** MYC-GFP-, WT DGCR8-, S677A-DGCR8-, and S677D-DGCR8-overexpressing LM2 cells with or without IR treatment (**a**, 8 Gy followed by 1-h incubation; **b**, 8 Gy followed by 8-h incubation) were subjected to pulldown with MYC beads and immunoblotting with the indicated antibodies. **c** HEK293T cells with stable overexpression of MYC-tagged WT DGCR8, S677A-DGCR8, or S677D-DGCR8 were co-transfected with SFB-USP51 (WT or the C372S mutant) and HA-tagged ubiquitin, and then treated with IR (8 Gy). After 8 h, cells were lysed, denatured, and subjected to immunoprecipitation with anti-MYC beads and immunoblotting with antibodies against HA and MYC. **d** Quantification of γH2AX, DGCR8, MDC1, RNF8, RNF168, 53BP1, and BRCA1 foci in DRCR8-knockdown LM2 cells with ectopic expression of WT DGCR8, S677A-DGCR8, or S677D-DGCR8. Cells were incubated for 1 h after 2-Gy IR and immunostained with antibodies against γH2AX, DGCR8, MDC1, RNF8, RNF168, 53BP1, and BRCA1 (see representative images in Supplementary Fig. 9). *n* = 3 biological replicates. Statistical significance was determined by a two-tailed unpaired *t*-test. Error bars are mean ± SEM. **e** DRCR8-knockdown LM2 cells with ectopic expression of WT DGCR8 or the S677A mutant were transduced with FLAG-H2A and RNF8 or RNF168. The cells were then transfected with HA-ubiquitin (Ub), treated with IR (8 Gy), and cultured for 8 h, followed by immunoprecipitation with anti-FLAG beads and immunoblotting with antibodies against HA and FLAG. Before immunoprecipitation, lysates were heated at 95 °C for 5 min in the presence of 1% SDS (for denaturing), followed by a 10-fold dilution with lysis buffer and sonication. LE long exposure, SE short exposure. Source data are provided as a Source Data file.

To further explore the mechanism of S677 phosphorylation of DGCR8 in DDR, we compared the interaction of WT DGCR8, the phosphodeficient mutant (S677A), or the phosphomimetic mutant (S677D) with MDC1, RNF8, RNF168, and USP51 at 1 or 8 h after IR. At both time points, radiation-induced interaction of DGCR8 with MDC1 and RNF8 was abolished by the phosphodeficient mutation S677A, whereas a phosphomimetic mutant of DGCR8 (S677D) showed constitutive interaction with MDC1 and RNF8, either with or without IR treatment (Fig. 8a, b). Consistent with the results in Supplementary Fig. 5b, the interaction between WT DGCR8 and USP51 was enhanced at 8 h, but not at 1 h, after IR, and this increased interaction was abolished by the phosphodeficient mutation (S677A), but not by the phosphomimetic mutation (S677D) (Fig. 8a, b). Moreover, USP51 markedly reduced the ubiquitination of either WT DGCR8 or the S677D mutant, but had little effect on the ubiquitination of the S677A mutant (Fig. 8c). In addition, immunofluorescent staining showed that the S677A mutation of DGCR8 did not affect the recruitment of MDC1 or RNF8 to DSBs, but abolished the localization of DGCR8, RNF168, 53BP1, and BRCA1 to damage sites, whereas the S677D mutant of DGCR8 exhibited similar effects to WT DGCR8 in irradiated cells (Fig. 8d and Supplementary Fig. 9). Finally, we found that the enhanced mono-, di-, and poly-ubiquitination of H2A by RNF8 and RNF168 upon IR was blocked by the S677A mutation (Fig. 8e). Collectively, these results suggest that after irradiation, ATM-dependent phosphorylation of DGCR8 at S677 is crucial for the recruitment of the DGCR8-RNF168 complex to MDC1 and RNF8 at DSBs, as well as the subsequent interaction of DGCR8 with USP51, DGCR8 deubiquitination, histone ubiquitination, and DSB repair.

## Discussion

DGCR8, a component of the microprocessor complex, is essential for miRNA biogenesis and plays important roles in development, oncogenesis, the exit of mouse embryonic stem cells from pluripotency, and the maintenance of heterochromatin organization^64–68^. Moreover, DGCR8 has been reported to regulate nucleotide excision repair of ultraviolet-induced lesions (pyrimidine dimers and base modifications)^46^ as well as degradation of double-stranded, structured RNAs^69^. In this study, we discovered that DGCR8 acts as a regulator of DSB repair and X-ray radiosensitivity independently of its Drosha-binding ability, revealing a new non-canonical role of DGCR8.

Aberrations in DSB repair underlie resistance to radiotherapy in patients with cancer. The RNF8/RNF168-mediated ubiquitination pathway governs the recruitment of DDR proteins to the chromatin surrounding DNA damage sites, which has critical roles in the repair of radiation-induced DSBs^7^. How RNF168 is recruited to genomic lesions remains elusive, and an X factor is hypothesized to be a missing link between RNF8 and RNF168^18^. Recently, histone H1^70,71^ and L3MBTL2^72^ have been reported as signaling intermediates between RNF8 and RNF168. Here we identify DGCR8 as a previously undescribed molecular link between RNF8 and RNF168. Upon IR, phosphorylation by ATM and subsequent stabilization by USP51 promote the recruitment of DGCR8 and its constitutive binding partner RNF168 to MDC1 and RNF8 at DSBs, resulting in amplified histone ubiquitination and enhanced DSB repair (Supplementary Fig. 10). These findings suggest that radiation treatment may lead to therapy-induced radioresistance through DGCR8. It is worth mentioning that in our study, depletion of RNF168 inhibited both HR and NHEJ repair in LM2 cells; however, the effect of RNF168 on HR repair seems to be complex and tissue type-dependent, since both HR-promoting and HR-inhibiting effects were reported^73–76^. Thus, future studies of the role of DGCR8 in HR repair in other tissue types are warranted.

Posttranslational modifications of DGCR8, including phosphorylation and SUMOylation, have been shown to regulate DGCR8’s stability and function^77,78^. However, the regulations and regulators of DGCR8 ubiquitination were largely unexplored. Our study revealed the impact of phosphorylation and ubiquitination on DGCR8, and both modifications are regulated by radiation. Importantly, we found that DGCR8 is phosphorylated by ATM at serine 677, which is critical for DGCR8 stabilization and the recruitment of DGCR8 to MDC1 and RNF8 at DSBs. Moreover, by screening a DUB library, we identified USP51 and USP36 as two DUBs that interact with DGCR8 and reduce its poly-ubiquitination in cells. Although both DUBs positively regulate DGCR8 protein levels, only USP51, but not USP36, is upregulated upon radiation and is required for IR-induced expression of DGCR8. The mechanism by which USP51 is upregulated by radiation remains to be determined. Furthermore, our results demonstrate that USP51 directly binds and deubiquitinates DGCR8 in a K48 linkage-specific manner, and that this deubiquitination is enhanced by IR treatment. In addition, our results demonstrate that USP51 facilitates histone ubiquitination and DSB repair. On the other hand, Wang et al. showed that USP51 can deubiquitinate H2A at later stages of DDR in U2OS cells, and that from 4 h post-IR, the 53BP1 protein recruited to DSBs diminished at a slower rate in USP51-depleted U2OS cells than in control cells^31^. Thus, future studies are needed to dissect the different effects of USP51 on H2A ubiquitination at early and late stages of DDR. In contrast to the reduction of proteolytic, K48-linked ubiquitination of DGCR8 by radiation, IR leads to an increase in non-proteolytic, K63-linked ubiquitination of DGCR8, and this type of ubiquitination is not affected by USP51 (Fig. 5d). It is worth mentioning that our mass spectrometric analysis identified several DGCR8-interacting ubiquitin ligases, including RNF8, RNF168, CHIP, HUWE1, ZFP91, RBBP6, and ZNF598. Future studies are needed to determine which E3 ligase regulates K48-linked ubiquitination of DGCR8, whether non-proteolytic ubiquitination of DGCR8 is involved in DDR, and which E3 ligase and deubiquitinase regulate K63-linked ubiquitination of DGCR8. Interestingly, the removal of K63-linked poly-ubiquitination of certain proteins, such as TXNIP and RIP1, can trigger K48-linked ubiquitination and degradation^79,80^. Thus, the relationship between K63-linked and K48-linked ubiquitination of DGCR8 during DDR is worth further investigation. Finally, our work calls for the development of small-molecule inhibitors that target USP51’s DUB activity or its interaction with DGCR8; such inhibitors have potential as tumor radiosensitizers.

## Methods

### Cell culture

The HEK293T, T47D, MCF-7, BT549, HepG2, HCT116, and HeLa cell lines were from the American Type Culture Collection (ATCC) and were cultured under conditions specified by the manufacturer. The LM2 cell line was from Xiang Zhang (Baylor College of Medicine) and the HEK293A cell line was from Junjie Chen’s lab stock; both cell lines were cultured in DMEM supplemented with 10% fetal bovine serum and 1% penicillin and streptomycin. Short tandem repeat profiling (for cell line authentication) and mycoplasma tests were done by ATCC and MD Anderson’s Characterized Cell Line Core Facility.

### Generation of radioresistant sublines

The radioresistant sublines were generated from parental LM2 and MCF-7 cell lines as described in our previous publication^5^. Briefly, after an 8-Gy dose of X-ray irradiation, surviving cells formed colonies. We pooled the colonies and repeated the dose two more times. The radiosensitivity of the cells derived from this selection, named LM2-R and MCF-7-R, was validated by clonogenic survival assays as described below. All experiments using these cells were performed at early passages.

### Chemicals

The chemicals used to treat cells were MG132 (Santa Cruz Biotechnology, #sc-201270), cycloheximide (Sigma, #C7698), puromycin (ThermoFisher Scientific, #A1113803), hygromycin B (ThermoFisher Scientific., #10687010), blasticidin (ThermoFisher Scientific, #R21001), G418 Sulfate (ThermoFisher Scientific, #MT30234CR), Micrococcal Nuclease (MNase) (ThermoFisher Scientific, #88216), the ATM inhibitor Ku55933 (Sigma, #SML1109), and the ATR inhibitor AZD-6738 (MedKoo, #206114).

### Plasmids and shRNA

The DGCR8 (#10921), Drosha (#10921), Dicer (#10921), Exportin-5 (#10921), HA-ubiquitin (WT: #17608; K48R: #17604; K48: #17605; K63: #17606), RNF8 (#99396), FLAG-H2A (#63560), p53 (#81754), pLCN DSB Repair Reporter (DRR) (#98895), and pCAGGS DRR mCherry Donor EF1a BFP (#98896) constructs were from Addgene. The RNF168 plasmid was from DNASU (clone ID: HsCD00832967). The pCBA-I-SceI plasmid was from Dr Junjie Chen’s lab stock. Full-length DGCR8 and the Drosha-binding domain deletion mutant (Δ692-DGCR8), as well as FLAG-H2A, were amplified by PCR and cloned into the pDONR201 vector through the BP reaction. The S677A (serine to alanine) and S677D (serine to aspartic acid) mutants of full-length DGCR8 and Δ692-DGCR8 in the pDONR201 vector were generated by using the QuikChange^®^ kit from Agilent Technologies and validated by sequencing. Full-length DGCR8, Δ692-DGCR8, and their S677A and S677D mutants, as well as FLAG-H2A, were subcloned into pcDNA3.1-MYC, pCDH-MYC, pLenti-CMV-puromycin, pLenti-CMV-hygromycin, pLX304, or MBP-tagged destination vectors through the LR reaction. The MBP-p53 construct was generated from pDONR-p53 (Addgene, #81754) through the LR reaction. The K63R mutant of HA-ubiquitin and the 68 human DUB ORFs in the pBabe-SFB vector were described in our previous publication^60^. FLAG-ATM and its kinase-dead mutant were described in our previous publication^5^. The GST-USP51 and SFB-USP51-C372S plasmids were described in our previous publication^63^. GFP and RNF168 were cloned into the GST-tagged destination vector through the LR reaction. USP36 and USP51 were subcloned from the pDONR201 vector into pLenti-CMV-puromycin and pLenti-CMV-hygromycin destination vectors through the LR reaction. The pLKO.1- DGCR8 shRNA (clone ID: NM_022720.5-2583s21c1 and NM_022720.4-575s1c1), USP51 shRNA (clone ID: NM_201286.1-669s1c1 and NM_201286.1-1915s1c1), USP36 shRNA (clone ID: NM_025090.3-4844s21c1 and NM_025090.3-1148s21c1), and RNF168 shRNA (clone ID: NM_152617.2-757s1c1 and NM_152617.2-2122s1c1) constructs were from Sigma. The pGIPZ- ATM shRNA (clone ID: V3LHS_350469 and V3LHS_350471) and ATR shRNA (clone ID: V2LHS_94659 and V2LHS_94661) constructs were from Dharmacon. RNAi-resistant DGCR8, Δ692-DGCR8, S677A-DGCR8, and S677D-DGCR8 constructs were generated by using the QuikChange^®^ kit from Agilent Technologies and were then subcloned into the pLenti-CMV-hygromycin destination vector through the LR reaction.

### Lentiviral transduction

Virus-containing supernatant was collected 48 h after co-transfection of the viral vector and packaging plasmids (psPAX2 and pMD2.G) into HEK293T cells, and was then added to the target cells. The infected cells were selected with puromycin, hygromycin B, blasticidin, or G418 for 3–14 days after infection.

### Immunoblotting

Western blot analysis was performed with precast gradient gels (Bio-Rad) using standard methods. Briefly, cultured cells were lysed in RIPA buffer (Millipore, #20-188) containing protease inhibitors (GenDEPOT, #P3100) and phosphatase inhibitors (GenDEPOT, #P3200). Proteins were separated by SDS-PAGE and blotted onto a nitrocellulose membrane (Bio-Rad). Membranes were blocked and then probed with the specific primary antibodies, followed by peroxidase-conjugated secondary antibodies. The bands were visualized by chemiluminescence (Denville Scientific). The following antibodies were used: antibodies against DGCR8 (1:2000, Abcam, #ab191875), Dicer (1:1000, Cell Signaling Technology, #5362S), Drosha (1:1000, Cell Signaling Technology, #3364S), Exportin-5 (1:1000, Cell Signaling Technology, #12565), γH2AX (1:1000, Cell Signaling Technology, #9718S), H2AX (1:1000, Cell Signaling Technology, #2595S), H2A (1:1000, Cell Signaling Technology, #2578S), p-CHK1 (1:1000, Cell Signaling Technology, #12302S), CHK1 (1:1000, Cell Signaling Technology, #2360S), p-CHK2 (1:1000, Cell Signaling Technology, #2661S), CHK2 (1:1000, Cell Signaling Technology, #6334S), p-ATM (1:1000, Cell Signaling Technology, #5883S), ATM (1:1000, Cell Signaling Technology, #2873S), p-ATR (1:1000, Cell Signaling Technology, #2853S), ATR (1:1000, Cell Signaling Technology, #2790S), p-S/TQ (1:1000, Cell Signaling Technology, 2851S), MBP (1:1000, Cell Signaling Technology, 2396S), GST (1:1000, Cell Signaling Technology, #2622S), MDC1 (1:1000, R&D Systems, #MAB6497), RNF8 (1:1000, Millipore, #09-813), RNF168 (1:1000, Millipore, #ABE367), USP36 (1:1000, a gift from Dr Masayuki Komada at Tokyo Institute of Technology), USP51 (1:3000, a gift from Dr Sharon Dent at MD Anderson Cancer Center), β-actin (1:1000, Santa Cruz Biotechnology, #sc-47778), FLAG (1:5000, Sigma, #F3165, clone M2), HA (1:2000, Santa Cruz Biotechnology, #sc-7392), and MYC (1:2000, Santa Cruz Biotechnology, #sc-40, clone 9E10). The antibody against phospho-DGCR8 (pS677; 1:500) was generated at Biomatik. The ImageJ program (version 1.53g) was used for densitometric analysis of western blots, and the quantification results were normalized to an internal control.

### Immunoprecipitation and pulldown assays

Cells were lysed in NETN buffer (20 mM Tris-HCl, pH 8.0, 100 mM NaCl, 1 mM EDTA, 0.5% Nonidet P-40) or CHAPS buffer (25 mM Tris-HCl, pH 7.5, 120 mM NaCl, 1 mM EDTA, 0.33% CHAPS) containing protease inhibitors and then sonicated. The chromatin fraction was isolated using a Chromatin Extraction Kit (Abcam, #ab117152), according to the manufacturer’s protocol, followed by sonication and with or without MNase treatment. For immunoprecipitation of endogenous proteins, cell extracts (lysed in CHAPS buffer) were pre-cleared with protein-A/G PLUS beads (Santa Cruz Biotechnology, #sc-2003) and IgG at 4 °C for 30 min, followed by incubation with an antibody against RNF8 (Proteintech, #14112-1-AP), RNF168 (Proteintech, #21393-1-AP), or DGCR8 (Bethyl Laboratories, #A302-468A) or IgG at 4 °C for 1 h, and were then incubated with protein-A/G PLUS beads at 4 °C overnight. The beads were washed with Tris-buffered saline containing 0.05% Tween-20 and CHAPS buffer. The bound proteins were eluted by incubation with 2× Laemmli buffer at room temperature for 10 min with mixing. For immunoprecipitation of tagged proteins, cell extracts were pre-cleared with protein-A/G PLUS beads and incubated with anti-MYC agarose beads (Millipore, #A7470) at 4 °C for 2 h. For pulldown of SFB-tagged proteins, cell extracts were incubated with S-protein beads (Millipore, #69704) at 4 °C for 2 h.

### In vitro binding assay

For DGCR8-RNF168 binding, bacterially purified MBP-DGCR8 was eluted with maltose and then incubated with glutathione beads (GE Healthcare, 17-0756-01) conjugated with bacterially purified GST-GFP or GST-RNF168 at 4 °C overnight. For DGCR8-USP51 binding, bacterially purified MBP-DGCR8 was incubated with mammalian purified SFB-USP51 or SFB-GFP from transfected HEK293T cells, followed by pulldown with S-protein beads at 4 °C overnight. The beads were washed with NETN buffer four times and the bound proteins were eluted by boiling in 2× Laemmli buffer and subjected to western blot analysis.

### Immunofluorescence

For immunostaining of γH2AX, DGCR8, MDC1, RNF8, RNF168, BRCA1, and 53BP1, cells were cultured in chamber slides (ThermoFisher Scientific) overnight, followed by X-ray irradiation. For immunostaining of DGCR8, USP36, and USP51, HEK293A cells were co-transfected with MYC-DGCR8 and SFB-tagged USP36 or USP51 and were cultured in chamber slides (ThermoFisher Scientific) overnight. The cells were washed with phosphate-buffered saline (PBS), fixed with 4% paraformaldehyde, permeabilized with 0.1% Triton X-100 in PBS, blocked with 3% bovine serum albumin in PBS, and incubated with antibodies against γH2AX (1:100, Cell Signaling Technology, #9718S), γH2AX (1:100, BD Biosciences, #560443), DGCR8 (1:100, Abcam, #ab191875), MDC1 (1:200, Bio-Rad, #AHP799), RNF8 (1:200, Proteintech, #14112-1-AP), RNF168 (1:100, Millipore, #ABE367), BRCA1 (1:20, Santa Cruz Biotechnology, #sc-6954), 53BP1 (1:100, Novus Biologicals, #NB100-304), FLAG (1:500, Sigma, #F7425), and MYC (1:500, Santa Cruz Biotechnology, #sc-40, clone 9E10) at 4 °C overnight, followed by incubation with Alexa Fluor 488 goat anti-rabbit IgG (1:1000, Invitrogen, ThermoFisher Scientific, #A-11008), Alexa Fluor 647 donkey anti-sheep IgG (1:1000, Invitrogen, ThermoFisher Scientific, #A-21448), Alexa Fluor 488 goat anti-mouse IgG (1:1000, Invitrogen, ThermoFisher Scientific, #A-11001), Alexa Fluor 594 goat anti-rabbit IgG (1:1000, Invitrogen, ThermoFisher Scientific, #A-11012), and/or Alexa Fluor 594 goat anti-mouse IgG (1:1000, Invitrogen, ThermoFisher Scientific, #A-11005) at room temperature for 1 h. Coverslips were mounted on slides by using anti-fade mounting medium with 4′,6-diamidino-2-phenylindole (DAPI, Vector Laboratories, #H-1200). Immunofluorescence images were acquired on a Zeiss LSM880 confocal microscope or Olympus FV1000 confocal microscope. Zen 2.6 (Zeiss) software was used for confocal image processing.

### RNA isolation and qPCR

Total RNA was isolated using TRIzol reagent (Invitrogen) and was then reverse transcribed with an iScript complementary DNA (cDNA) Synthesis Kit (Bio-Rad, #1708891). The resulting cDNA was used for real-time PCR with the iTaq Universal SYBR Green Supermix (Bio-Rad, #1725124). *GAPDH* was used as an internal control. Real-time PCR and data collection were performed on a CFX96 instrument (Bio-Rad). The primer sequences are listed in Supplementary Table 1.

### Mass spectrometry

To identify DGCR8-interacting proteins in LM2 cells with or without IR treatment, we performed the tandem-affinity purification and mass spectrometry as follows. LM2 cells were transfected with SFB-tagged DGCR8. Forty-eight hours after transfection, the cells were cultured for 1 h after release from 8 Gy X-rays. Expression of the transfected protein was confirmed by immunoblotting. For affinity purification, a total of ten 15-cm dishes of LM2 cells expressing SFB-DGCR8 were lysed in NETN buffer (200 mM Tris-HCl, pH 8.0, 100 mM NaCl, 0.05% Nonidet P-40, 1 mM EDTA) containing protease inhibitors at 4 °C for 20 min and then sonicated. Crude lysates were cleared by centrifugation, and the supernatants were incubated with 300 µl streptavidin-sepharose beads (GE Healthcare, #17511301) at 4 °C for 2 h. The beads were washed three times with NETN buffer, and the bound proteins were eluted with NETN buffer containing 2 mg/ml biotin (Sigma, #B4501) at 4 °C for 2 h. The eluates were incubated with 100 µl S-protein agarose beads (Millipore, #69704) at 4 °C for 2 h, and the beads were washed three times with NETN buffer. The bound proteins were eluted by boiling in 2× Laemmli buffer, resolved by SDS-PAGE, visualized by Coomassie blue staining, and subjected to in-gel digestion^81^.

For identifying the phosphorylation site(s) on DGCR8 upon IR, MYC-tagged DGCR8 protein immunoprecipitated from non-irradiated or irradiated LM2 cells was resolved by SDS-PAGE and visualized by Coomassie Brilliant Blue staining. DGCR8 bands were excised, destained with 50% ethanol, and dehydrated with acetonitrile. After reduction and alkylation with dithiothreitol and iodoacetamide, bands were then subjected to in-gel digestion with trypsin (enzyme:protein = 1:50) at 37 °C for 8 h. The tryptic peptides were extracted with water and acetonitrile from the gel after digestion. The extracted peptides were dried by Speed-Vac and then subjected to high-performance liquid chromatography-tandem mass spectrometry analysis as follows. Tryptic peptides from in-gel digestion were dissolved in HPLC solvent A (0.1% formic acid in 2% acetonitrile and 98% H_2_O), injected onto a manually packed reversed-phase C18 column (170 mm × 79 μm, 3-μm particle size, Dikma, China) coupled to an Easy-nLC 1200 chromatography system (ThermoFisher Scientific). Peptides were eluted using a 1-h gradient of 8–80% solvent B (0.1% formic acid in 90% acetonitrile and 10% H_2_O) in solvent A at a flow rate of 300 nl/min. Peptides eluted from HPLC were ionized with a nanospray source and analyzed in an Orbitrap HF-X mass spectrometer (ThermoFisher Scientific).

For the identification of the DGCR8 phosphorylation site, acquired MS raw files were transformed into MGF format using the Proteome Discoverer software (version 2.2, ThermoFisher Scientific). Proteins were identified by comparing the fragment spectra against those in the UniProt database using Mascot v.2.6.01 (Matrix Science Ltd., London, UK). For the identification of DGCR8-interacting proteins, acquired MS raw files were searched against the human proteome database from UniProt (January 26, 2019, updated, 93,798 sequences) using MaxQuant software (version 1.6.7.0), with a reversed decoy database by Andromeda search engine^82^. The default parameters for label-free quantification (LFQ) were applied^82^. Matching between runs was performed, and LFQ was performed with a minimum ratio count of 2^83^. The iBAQ values were used to calculate the absolute protein abundance^84,85^.

### In vivo and in vitro deubiquitination assays

For in vivo deubiquitination, HEK293T cells were harvested 48 h after transfection with the indicated plasmids. For denaturing, lysates were heated at 95 °C for 5 min in the presence of 1% SDS, followed by a 10-fold dilution with lysis buffer (to 0.1% SDS) and sonication as described in our previous publication^60^. The cell lysates were incubated with S-protein or anti-MYC beads for 2 h, and then the beads were washed with lysis buffer three times and subjected to western blot analysis with the indicated antibodies. For in vitro deubiquitination, HEK293T cells were transfected with HA-ubiquitin and MYC-DGCR8 plasmids; 48 h after transfection, MYC-DGCR8 was immunoprecipitated with anti-MYC beads and incubated with purified SFB-USP51 protein (WT or the C372S mutant) at 37 °C for 3 h in deubiquitination buffer (50 mM Tris-HCl, pH 8.0, 50 mM NaCl, 10 mM dithiothreitol, 1 mM EDTA, 5% glycerol). After the reaction, the beads were washed with deubiquitination buffer, and the bound proteins were eluted by boiling in 1× Laemmli buffer and subjected to western blot analysis with the indicated antibodies.

### HR and NHEJ repair assays

HR and NHEJ repair assays were performed as described previously^55^. Briefly, LM2 cells were infected with pLCN DRR lentivirus. After G418 selection for 14 days, a single clone was established as the DRR cell line (named LM2-DRR). The LM2-DRR cells were transduced with lentivirus expressing control shRNA or shRNA targeting DGCR8, RNF168, or USP51. After puromycin selection for 2 days, the DGCR8-knockdown cells were transduced with RNAi-resistant DGCR8, S677A-DGCR8, or S677D-DGCR8, and then selected for another 7 days. The cells were co-transfected with I-SceI endonuclease (pCBA-I-SceI, to induce DSBs) and pCAGGS DRR mCherry Donor EF1a BFP (an exogenous donor for HR). The cells were harvested 2 days after transfection and subjected to flow cytometric analysis (Attune NxT Flow Cytometer, Invitrogen, ThermoFisher Scientific) to determine the percentage of mCherry-positive and GFP-positive cells resulting from HR- and NHEJ-based repair, respectively. The ratio of mCherry-positive or GFP-positive cells to BFP-positive cells was used as a measure of HR or NHEJ repair efficiency. Attune™ NxT software (version H.0) was used for flow cytometry data collection and analysis. A representative gating strategy for flow cytometric analysis is shown in Supplementary Fig. 11.

### Clonogenic survival assay

Equal numbers of cells were plated in 6-well plates or 10-cm tissue-culture dishes in triplicates at a clonogenic density and irradiated by using an X-RAD 320 irradiator with the indicated doses. The medium was replaced 24 h later and the cells were then incubated for 12–18 days. The resulting colonies were fixed and stained with crystal violet. Colonies containing more than 50 cells were counted. The survival fraction was calculated as: (number of colonies/number of cells plated)_irradiated_/(number of colonies/number of cells plated)_non-irradiated_.

### In vitro kinase assay

HEK293T cells were transfected with 12 µg of WT FLAG-ATM or the kinase-dead mutant and then irradiated. Activated or kinase-dead ATM was immunopurified from the cell extracts with anti-FLAG beads (ThermoFisher Scientific, #A36797). Recombinant MBP-tagged full-length DGCR8 or Δ692- DGCR8, their S677A mutants, and p53 were expressed in bacteria and purified with maltose. The recombinant proteins were mixed with purified ATM protein and ATP (Cell Signaling Technology, #9804) in kinase buffer (Cell Signaling Technology, #9802). The samples were incubated at 30 °C for 15 min in a hybridization oven-shaker (ThermoFisher Scientific). The reactions were terminated by boiling, and the proteins were subjected to western blot analysis with antibodies against p-S/TQ, p-ATM, ATM, and MBP.

### Tissue microarray and immunohistochemical analysis

Human breast tumor tissue microarrays containing 139 analyzable cases of breast carcinoma were purchased from US Biolab (#HBreD139Su01). Tissue specimens were processed as described in our previous publication^5^. The primary antibodies used for immunohistochemical analysis were antibodies against DGCR8 (1:250, Abcam, #ab90579) and USP51 (1:250, Abcam, #ab121147). Whole-slide images were captured using an Aperio CS2 slide scanner system (Leica Biosystems, Wetzlar, Germany) at a 40× magnification. Immunohistochemical staining was analyzed by using the immunoreactive score (IRS) system. The percentage of positive cells was scored on a scale of 0–4: 0 if 0% of tumor cells were positive, 1 if 1–10% of cells were positive, 2 if 11–50% were positive, 3 if 51–80% were positive, and 4 if 81–100% were positive. Staining intensity was scored on a scale of 0–3 (3 is the strongest). Final IRS score = (score of the staining intensity) × (score of the percentage of positive cells). Score = 0–6 was considered moderate or weak expression and score = 8–12 was considered strong expression.

### Tumor radiosensitivity study

Animal experiments were performed as described previously^86^, in accordance with a protocol approved by the Institutional Animal Care and Use Committee of MD Anderson Cancer Center. Mice were housed at 70–74 °F (set point: 72 °F) with 40–55% humidity (set point: 45%). The light cycle of animal rooms is 12 h of light and 12 h of dark. Mice were euthanized when they met the institutional euthanasia criteria for tumor size or overall health condition. Solitary tumor xenografts were produced in the muscle of the right hind limbs of 10-week-old female nude mice (from MD Anderson Cancer Center’s internal supply) by inoculation of 3 × 10^6^ control, DGCR8-knockdown, or DGCR8-restored (with either WT DGCR8 or Δ692-DGCR8) LM2-R cells. Mice were randomly assigned to no treatment or treatment groups consisting of ten mice per group. Radiation treatment was initiated when tumors grew to approximately 8.0 mm in diameter. Fractionated doses of X-rays (2 Gy per fraction, once daily for 5 consecutive days a week × 2 weeks) were delivered to the tumor-bearing limbs by using an X-RAD 320 irradiator. During irradiation, unanesthetized mice were mechanically immobilized in a jig such that the tumor was exposed in the radiation field and the animal’s body was shielded from radiation exposure. Three mutually orthogonal diameters of the tumor were measured every other day with a Vernier caliper, and the mean value was calculated and used as the tumor diameter. The investigator (L. W.) who measured tumor size was blinded to the group allocation during animal experiments and outcome assessments.

### Statistics and reproducibility

Each experiment was independently repeated three times, except for the animal study (one time), DUB screening (one time), tissue microarray and immunohistochemical analysis (one time), and mass spectrometric analysis (two times); the representative data are shown. Unless otherwise noted, data are presented as mean ± SEM of three biologically independent samples. Statistical significance was determined by Student’s *t-*test (two-tailed, unpaired), *χ*^2^ test, Pearson analysis (two-sided), or log-rank test as indicated, using GraphPad Prism 8.0 or SPSS 25.0. The statistical analysis for each plot was described in figure legends. *P* < 0.05 was considered statistically significant.

### Reporting summary

Further information on research design is available in the Nature Research Reporting Summary linked to this article.

## Supplementary information

Supplementary Information

Reporting Summary

## Data Availability

The raw data and processed data for mass spectrometric analysis of DGCR8-interacting proteins and DGCR8 phosphorylation sites have been deposited to the MassIVE database with the identifier MSV000087301. UniProt is a free public resource of protein sequence and functional information (https://www.uniprot.org/). The uncropped gels and blots are shown in Supplementary Fig. 12. Source Data are provided with this paper.

## Source data

Source Data
