# Supplementary material for: Non-canonical function of DGCR8 in DNA double-strand break repair signaling and tumor radioresistance
Source: Nat Commun. 2021 Jun 29;12:4033. doi: 10.1038/s41467-021-24298-z (PMC8242032; doi:10.1038/s41467-021-24298-z)
Supplement: Supplementary file 1 — Supplementary Information [file 41467_2021_24298_MOESM1_ESM.pdf]

Supplementary Information

Non-canonical function of DGCR8 in DNA double-strand break repair signaling and tumor radioresistance

Qinglei Hang[1], Liyong Zeng[1], Li Wang[2], Litong Nie[1], Fan Yao[1,5], Hongqi Teng[1], Yalan Deng[1], Shannon Yap[1], Yutong Sun[3], Steven J. Frank[2], Junjie Chen[1,4], Li Ma[1,4,*]

[1]Department of Experimental Radiation Oncology, The University of Texas MD Anderson Cancer Center, Houston, TX 77030, USA

[2]Department of Radiation Oncology, The University of Texas MD Anderson Cancer Center, Houston, TX 77030, USA

[3]Department of Molecular and Cellular Oncology, The University of Texas MD Anderson Cancer Center, Houston, TX 77030, USA

[4]The University of Texas MD Anderson UTHealth Graduate School of Biomedical Sciences, Houston, TX 77030, USA

[5]Present address: College of Biomedicine and Health, College of Life Science and Technology, Huazhong Agricultural University, Wuhan, Hubei 430070, China

*Correspondence: lma4@mdanderson.org (L.M.)

Supplementary Figure 1

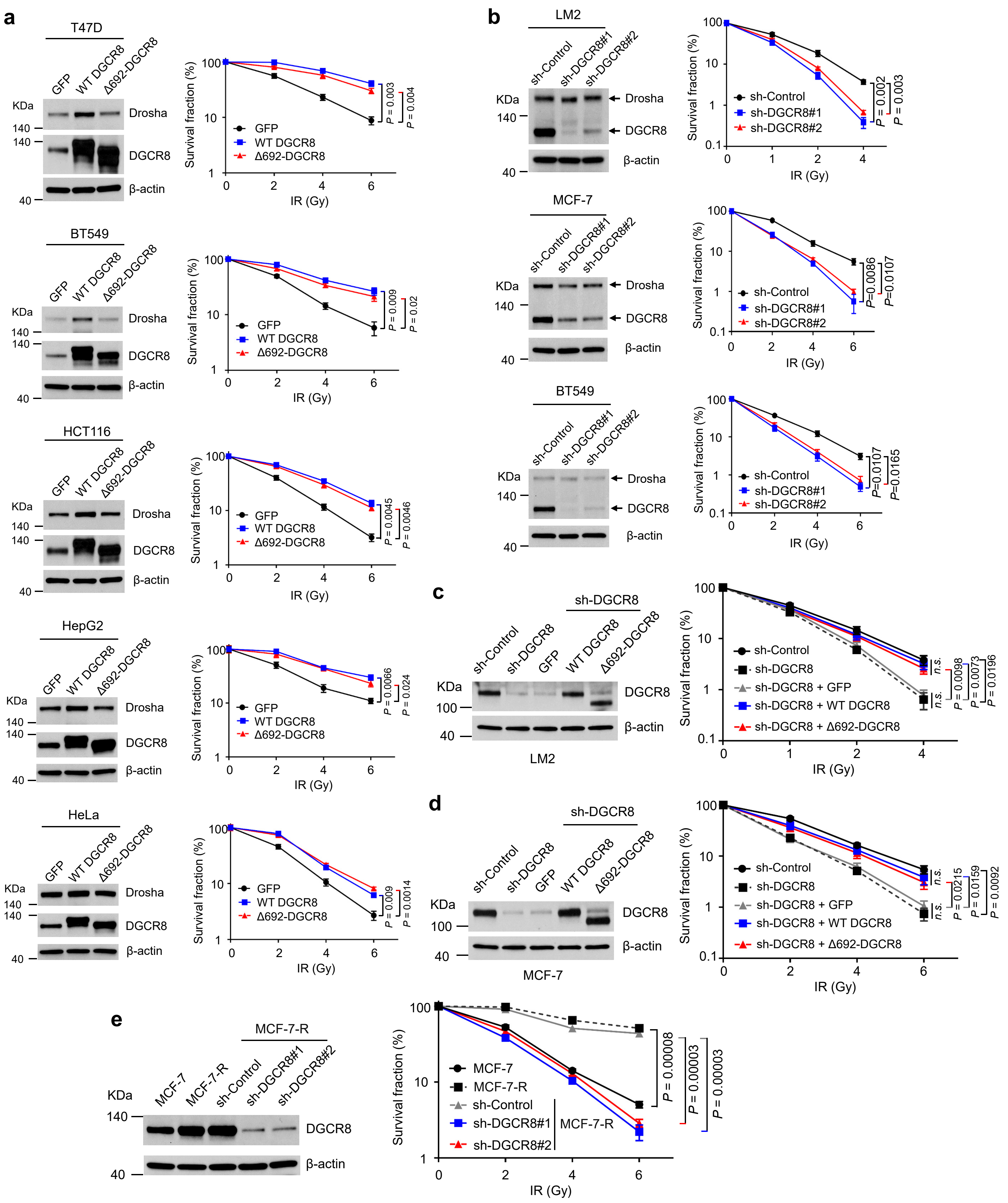

Supplementary Figure 1. DGCR8 is a positive regulator of X-ray radioresistance.

(**a**) Immunoblotting of Drosha, DGCR8, and β-actin (left panels) and clonogenic survival assays (right panels) of T47D, BT549, HCT116, HepG2, and HeLa stable cell lines overexpressing GFP, wild-type DGCR8, or Δ692-DGCR8. $n = 3$ wells per group.

(**b**) Immunoblotting of Drosha, DGCR8, and β-actin (left panels) and clonogenic survival assays (right panels) of LM2, MCF-7, and BT549 stable cell lines with knockdown of DGCR8. Two independent shRNAs (#1 and #2) were used. $n = 3$ wells per group.

(**c** and **d**) Immunoblotting of DGCR8 and β-actin (left panel) and clonogenic survival assays (right panel) of DGCR8-knockdown LM2 (**c**) and MCF-7 (**d**) cells with ectopic expression of wild-type DGCR8 or Δ692-DGCR8. $n = 3$ wells per group.

(**e**) Immunoblotting of DGCR8 and β-actin (left panel) and clonogenic survival assays (right panel) of MCF-7 and MCF-7-R cells transduced with DGCR8 shRNA. $n = 3$ wells per group.

Statistical significance in **a-e** was determined by a two-tailed unpaired *t*-test. Error bars are mean ± s.e.m. *n.s.*: not statistically significant. Source data are provided as a Source Data file.

Supplementary Figure 2

a

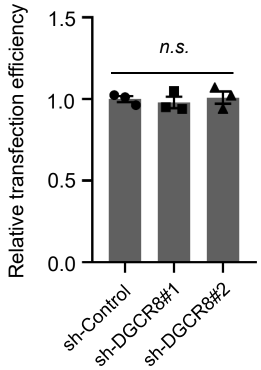

b

Protein name	Average iBAQ value (log10)	
	IR (–)	IR (+)
DGCR8	10.04294	10.04925
MDC1	N/A	8.28422
RNF8	N/A	7.82521
RNF168	8.820458	8.817493
XRCC6	8.343402	8.284378
XRCC5	8.281026	8.171007
PARP1	7.969057	7.866407
PRPF19	7.767542	7.763358
FEN1	7.647337	7.665059
PCNA	7.500721	7.507456
RPA1	7.289626	7.328264
PRKDC	7.291864	7.273087
TP53	7.117106	7.211085
MSH2	7.15488	7.002437
MSH6	7.042795	6.956237
DDB1	6.72611	6.942646
CHEK1	6.395338	6.460256

c

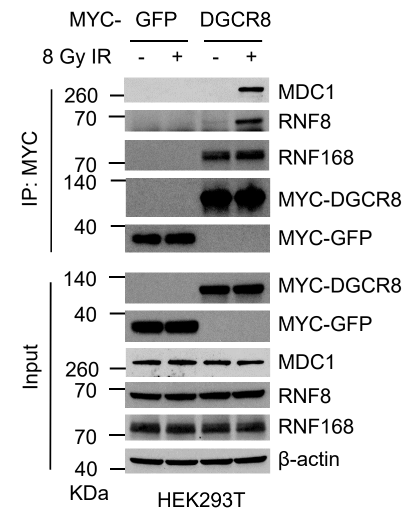

d

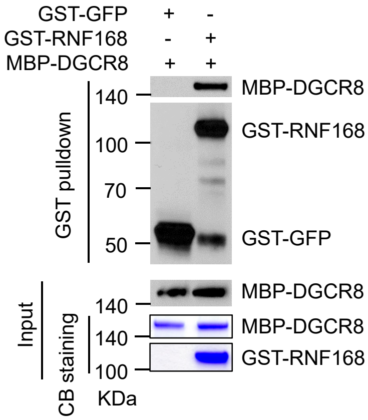

e

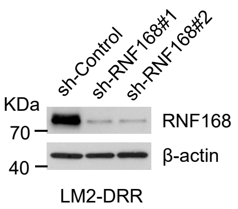

f

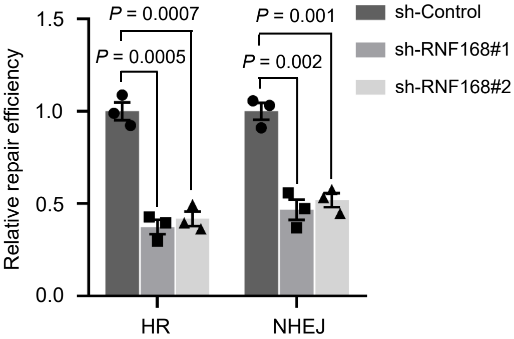

g

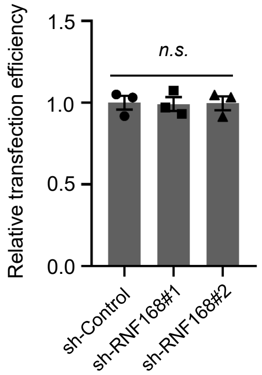

Supplementary Figure 2. DGCR8 interacts with RNF168, RNF8, and MDC1 in irradiated cells.
(**a**) Relative transfection efficiency of the cells used in **Fig. 3b**, gauged by an exogenous donor (pCAGGS DRR mCherry Donor EF1a BFP) that expresses BFP. $n = 3$ biological replicates.
(**b**) List of DGCR8-associated DNA damage response proteins in LM2 cells with or without IR treatment, identified by mass spectrometric analysis. The average iBAQ value was from 3 biological replicates.
(**c**) MYC-DGCR8-overexpressing HEK293T cells were treated with IR (8 Gy) and cultured for 1 hour, followed by pulldown with MYC-beads and immunoblotting with the indicated antibodies.
(**d**) DGCR8 binds RNF168 *in vitro*. Upper panel: purified GST-GFP or GST-RNF168 was incubated with purified MBP-DGCR8, followed by pulldown with glutathione-agarose beads and immunoblotting with antibodies against MBP and GST. Lower panel: purified recombinant proteins were analyzed by SDS-PAGE, Coomassie blue (CB) staining, and immunoblotting with an antibody against MBP.
(**e-g**) Knockdown of RNF168 decreased HR and NHEJ efficiency in LM2-DRR cells. **e**, Immunoblotting of RNF168 and β-actin in the LM2-DRR (pLCN DSB Repair Reporter) cell line transduced with RNF168 shRNA. **f**, Two days after co-transfection of I-SceI endonuclease and an exogenous donor for HR (pCAGGS DRR mCherry Donor EF1a BFP) into the RNF168-knockdown LM2-DRR cells, the percentages of GFP-positive and mCherry-positive cells, gated on BFP-positive cells, were determined by flow cytometry. Repair by HR or NHEJ leads to mCherry or GFP expression. Data were normalized to the control cells. $n = 3$ biological replicates. **g**, Relative transfection efficiency was gauged by the percentage of BFP-positive cells. Data were normalized to the control cells. $n = 3$ biological replicates.
Statistical significance in **a**, **f**, and **g** was determined by a two-tailed unpaired *t*-test. Error bars are mean ± s.e.m. *n.s.*: not statistically significant. Source data are provided as a Source Data file.

Supplementary Figure 3

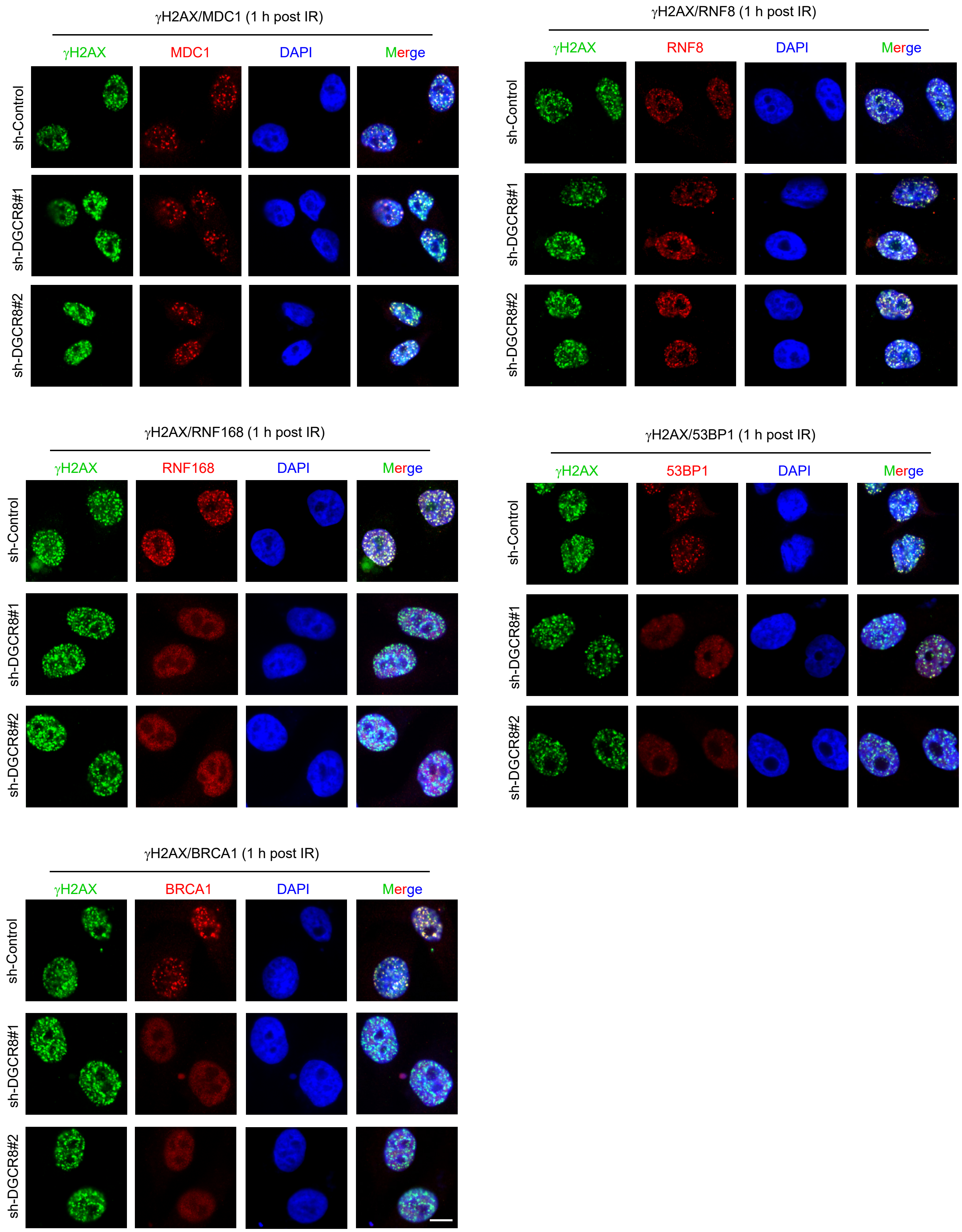

Supplementary Figure 3. Representative images of γH2AX, MDC1, RNF8, RNF168, 53BP1, and BRCA1 foci in DGCR8-knockdown LM2 cells.

Cells were incubated for 1 hour after 2-Gy IR and immunostained with antibodies against γH2AX, MDC1, RNF8, RNF168, 53BP1, and BRCA1 (see data quantification in **Fig. 3f**). Scale bars, 10 μm.

Supplementary Figure 4

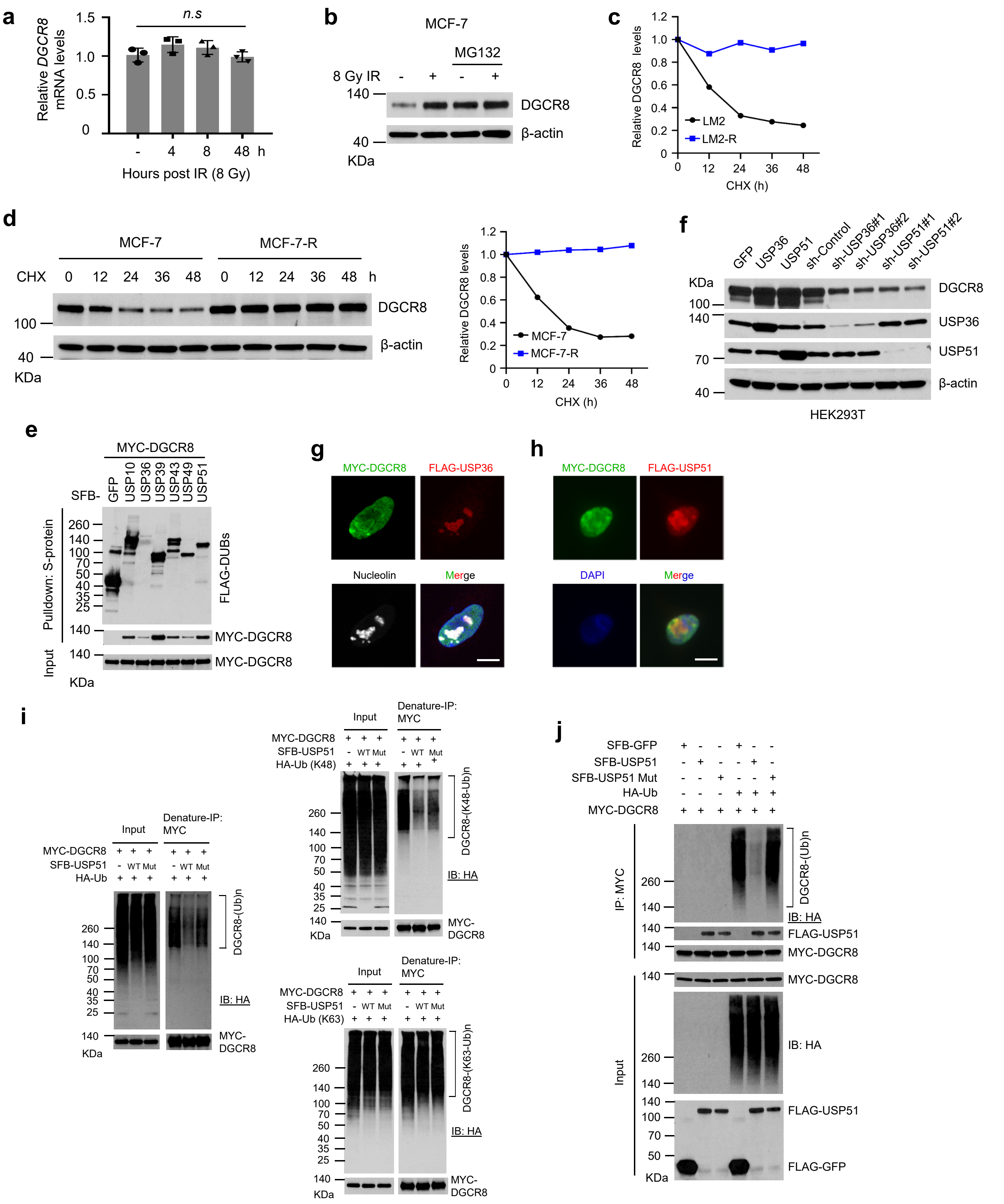

Supplementary Figure 4. Radiation upregulates DGCR8 and USP51 proteins, and USP51 deubiquitinates DGCR8 *in vivo* and *in vitro*.

(**a**) qPCR of *DGCR8* in LM2 cells at the indicated times after X-ray IR treatment. n = 3 biological replicates. *n.s.*: not statistically significant based on a two-tailed unpaired t-test. Error bars are mean ± s.e.m.

(**b**) MCF-7 cells were treated with 10 μM MG132, irradiated with 8 Gy IR, and collected 6 hours later. Lysates were immunoblotted with antibodies against DGCR8 and β-actin.

(**c**) Quantification of DGCR8 protein levels (normalized to β-actin) in **Fig. 4b**.

(**d**) Left panel: parental and radioresistant MCF-7 cells were treated with 50 μg/ml cycloheximide (CHX). Cells were collected at the indicated times and immunoblotted with antibodies against DGCR8 and β-actin. Right panel: DGCR8 levels were quantitated and normalized to β-actin.

(**e**) Six candidate DUBs were individually co-transfected with MYC-DGCR8 into HEK293T cells, followed by pulldown with S-protein beads and immunoblotting with antibodies against FLAG and MYC.

(**f**) Immunoblotting of DGCR8, USP36, USP51, and β-actin in HEK293T cells with overexpression or knockdown of USP36 or USP51.

(**g**) Immunofluorescent staining of MYC-DGCR8, FLAG-USP36, and nucleolin (a marker of the nucleolus) in HEK293A cells. Scale bar, 20 μm.

(**h**) Immunofluorescent staining of MYC-DGCR8, FLAG-USP51, and DAPI in HEK293A cells. Scale bar, 20 μm.

(**i**) *In vivo* deubiquitination assay. HEK293T cells were co-transfected with MYC-DGCR8, HA-tagged ubiquitin (left panel) or the lysine-specific mutant (K48 or K63, right panels), and SFB-USP51 (wild-type or the C372S mutant). After treatment with MG132 for 6 hours, cells were lysed, denatured, and subjected to pulldown with anti-MYC beads and immunoblotting with antibodies against HA and MYC.

(**j**) *In vitro* deubiquitination assay. Non-ubiquitinated or ubiquitinated MYC-DGCR8 was incubated with SFB-USP51 (wild-type or the C372S mutant) purified from HEK293T cells with streptavidin-Sepharose beads. After the reaction, MYC-DGCR8 was immunoprecipitated with anti-MYC beads and immunoblotted with antibodies against HA, FLAG, and MYC.

Source data are provided as a Source Data file.

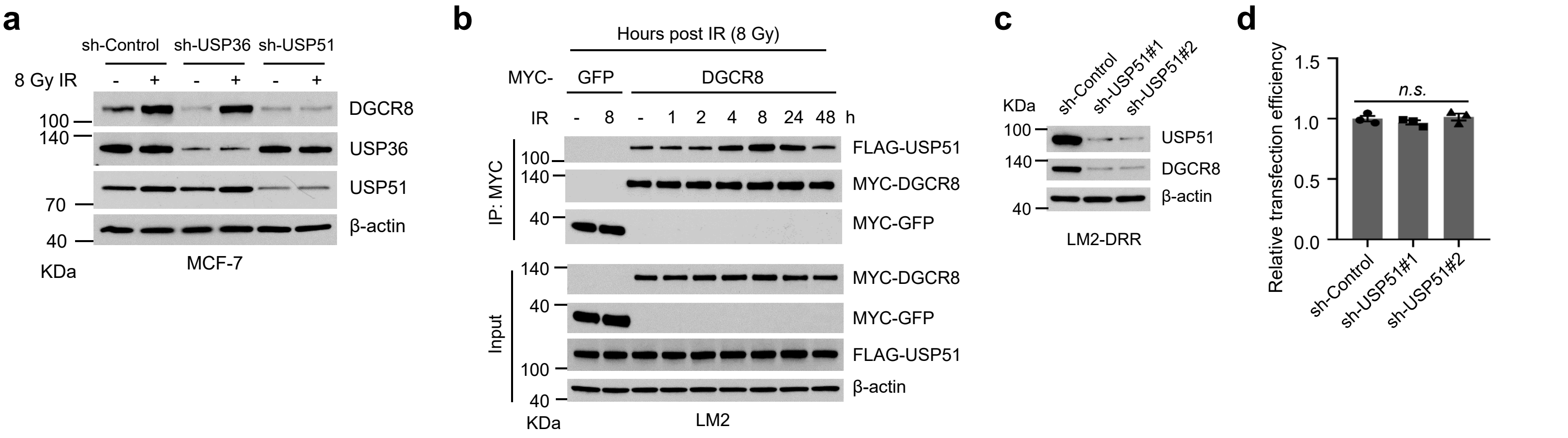

Supplementary Figure 5. Knockdown of USP51 downregulates DGCR8 protein.

(**a**) Immunoblotting of DGCR8, USP36, USP51, and β-actin in USP36-knockdown and USP51-knockdown MCF-7 cells with or without IR treatment (8 Gy followed by 24-hour incubation).

(**b**) LM2 cells with ectopic expression of MYC-DGCR8 and SFB-USP51 were treated with IR (8 Gy). Cells were collected at the indicated times, followed by immunoprecipitation with anti-MYC beads and immunoblotting with antibodies against FLAG and MYC.

(**c**) Immunoblotting of USP51, DGCR8, and β-actin in the LM2-DRR (pLCN DSB Repair Reporter) cell line transduced with USP51 shRNA.

(**d**) Relative transfection efficiency of the cells used in **Fig. 5e**, gauged by an exogenous donor (pCAGGS DRR mCherry Donor EF1a BFP) that expresses BFP. n = 3 biological replicates. Error bars are mean ± s.e.m. *n.s.*: not statistically significant based on a two-tailed unpaired t-test. Source data are provided as a Source Data file.

Supplementary Figure 6

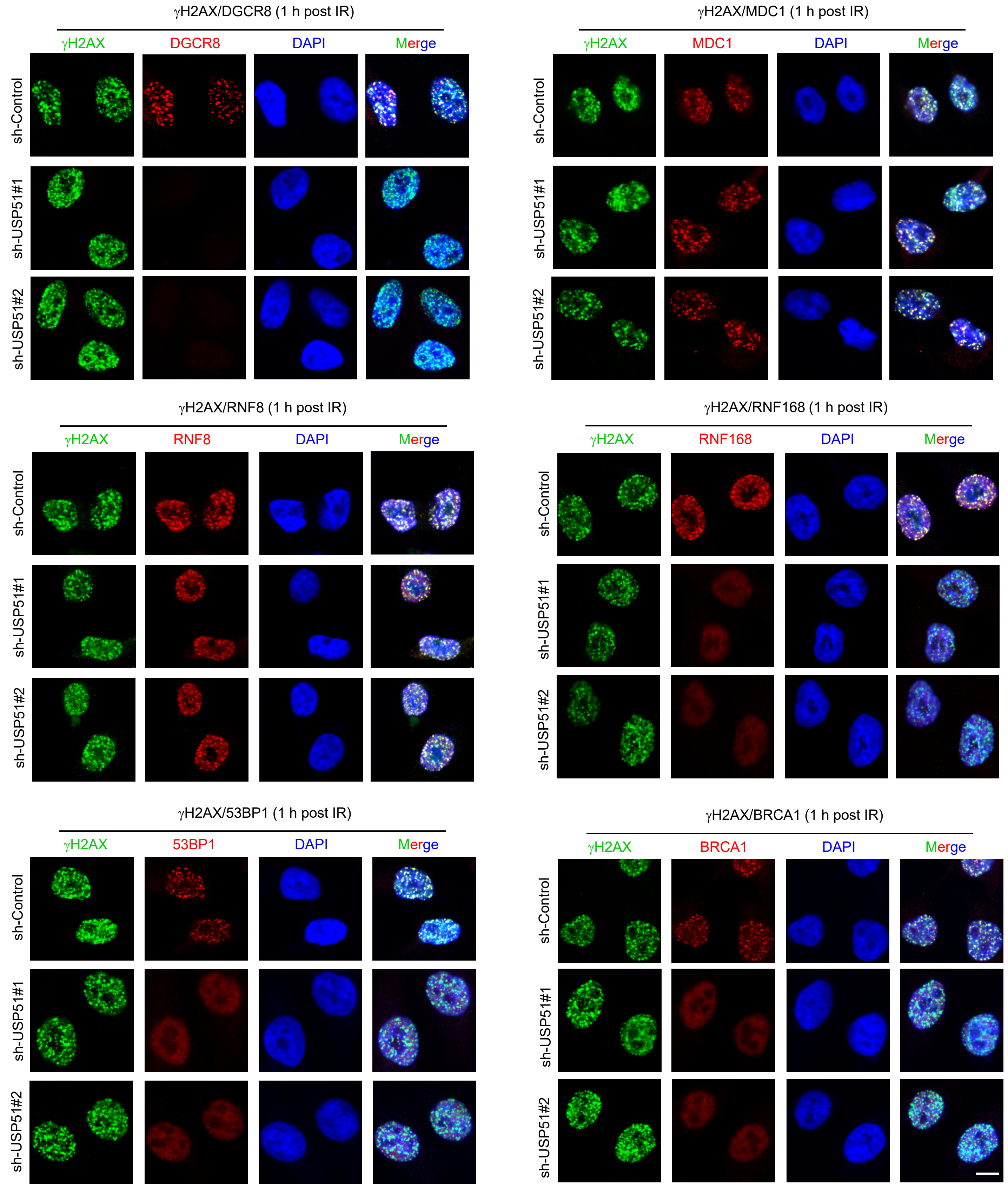

Supplementary Figure 6. Representative images of γH2AX, DGCR8, MDC1, RNF8, RNF168, 53BP1, and BRCA1 foci in USP51-knockdown LM2 cells.

Cells were incubated for 1 hour after 2-Gy IR and immunostained with antibodies against γH2AX, DGCR8, MDC1, RNF8, RNF168, 53BP1, and BRCA1 (see data quantification in **Fig. 5f**). Scale bars, 10 μm.

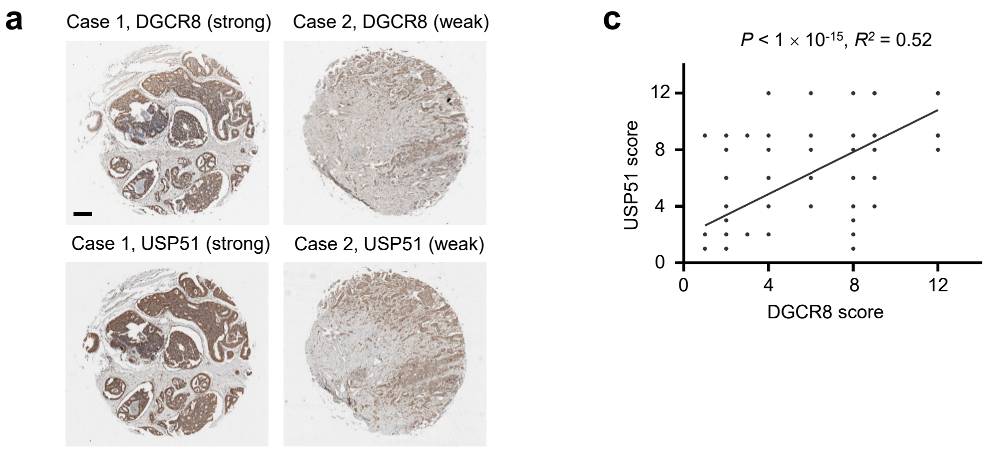

b

$P = 3 \times 10^{-9}$, $R = 0.48$

	DGCR8 Moderate/weak	DGCR8 Strong	Total
USP51 Moderate/weak	61	14	75
USP51 Strong	22	42	64
Total	83	56	139

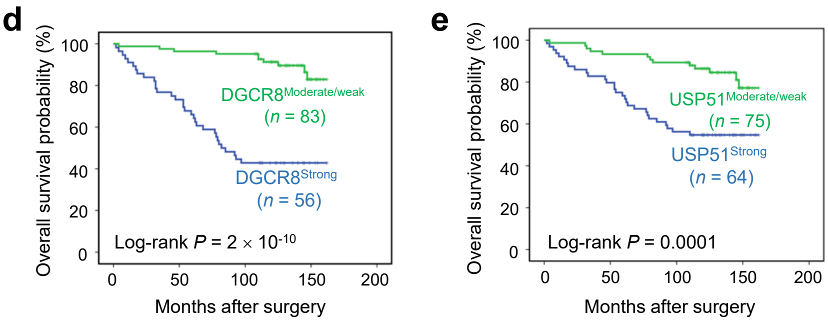

Supplementary Figure 7. DGCR8 correlates with USP51 expression and poor survival in patients with breast cancer.

(**a**) Immunohistochemical staining of DGCR8 and USP51 in representative breast tumor specimens. Scale bar, 500 µm.

(**b**) Correlation between DGCR8 and USP51 protein levels in human breast tumors. The *P* value was calculated from a χ^2 test (two-sided). *R* is the Spearman correlation coefficient.

(**c**) DGCR8 protein scores (x-axis) in primary breast tumors positively correlate with USP51 protein scores (y-axis) in individual patients. The *P* value was calculated from Pearson analysis (two-sided). *R* is the correlation coefficient. Protein score = (score of the staining intensity) × (score of the percentage of positive cells).

(**d** and **e**) Kaplan-Meier analysis of overall survival in a cohort of breast cancer patients (n = 139), stratified by the protein level of DGCR8 (**d**) or USP51 (**e**). Statistical significance was determined by a log-rank test. Source data are provided as a Source Data file.

Supplementary Figure 8

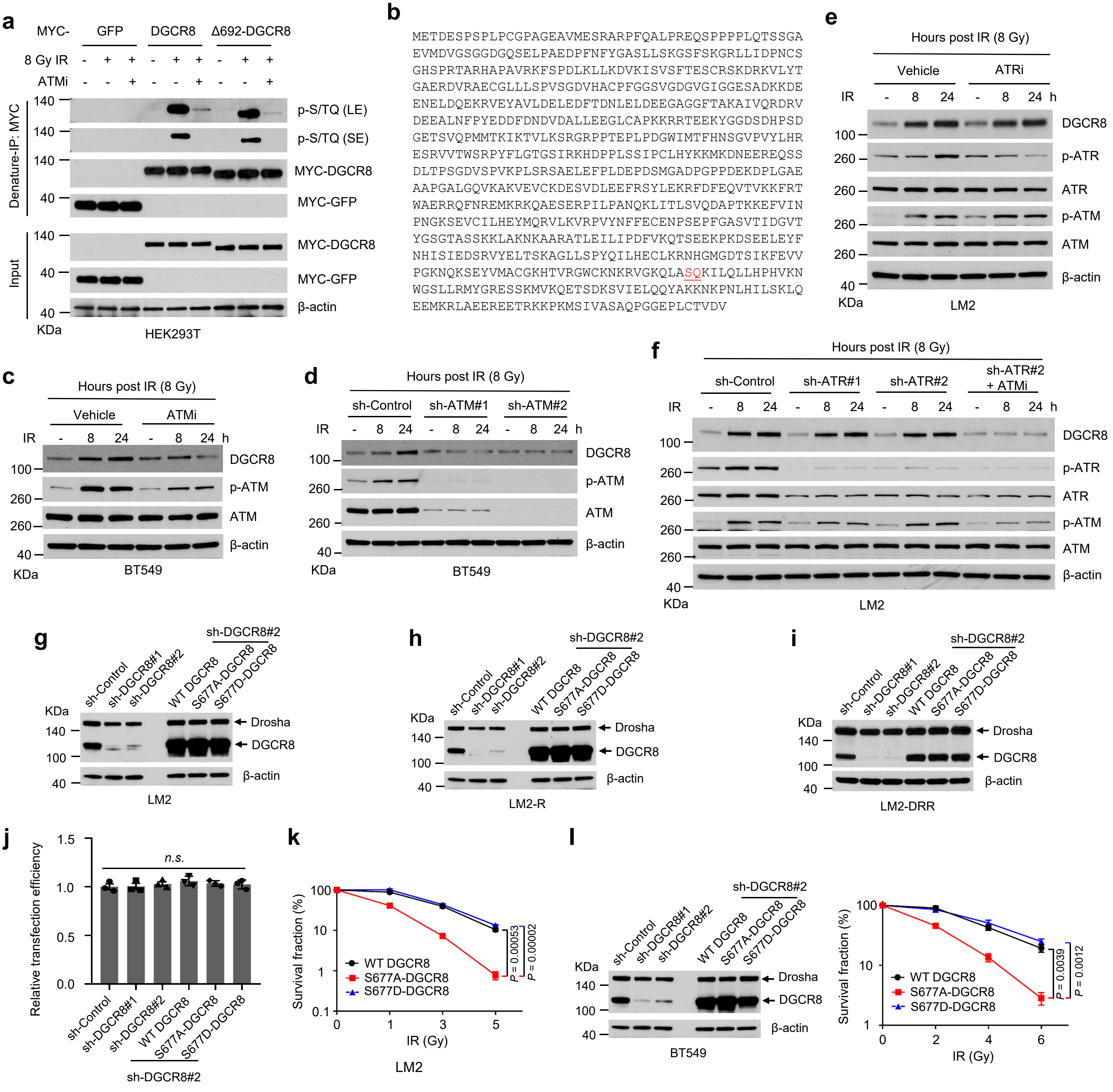

Supplementary Figure 8. DGCR8 is regulated by ATM but not ATR.

(**a**) MYC-GFP-, DGCR8-, and Δ692-DGCR8-overexpressing HEK293T cells with or without ATM inhibitor (ATMi) Ku55933 pretreatment (10 μM, 1 hour) were treated with IR (8 Gy) and cultured for 30 minutes, followed by pulldown with anti-MYC beads and immunoblotting with antibodies against p-S/TQ and MYC. LE: long exposure; SE: short exposure.

(**b**) The amino acid sequence of human DGCR8 with the putative ATM phosphorylation site underlined and in red.

(**c**) BT549 cells were pretreated with ATMi (Ku55933, 10 μM for 1 hour), followed by IR treatment (8 Gy). Lysates were immunoblotted with antibodies against DGCR8, p-ATM, ATM, and β-actin.

(**d**) Immunoblotting of DGCR8, p-ATM, ATM, and β-actin in control and ATM-knockdown BT549 cells collected at the indicated times after IR.

(**e**) LM2 cells were pretreated with the ATR inhibitor (ATRi) AZD6738 (10 μM for 1 hour), followed by treatment with IR (8 Gy). Lysates were immunoblotted with antibodies against DGCR8, p-ATR, ATR, p-ATM, ATM, and β-actin.

(**f**) Immunoblotting of DGCR8, p-ATR, ATR, p-ATM, ATM, and β-actin in control and ATR-knockdown LM2 cells collected at the indicated times after IR (8 Gy). One group was pretreated with the ATM inhibitor (ATMi) Ku55933 (10 μM for 1 hour).

(**g-i**) Immunoblotting of DGCR8, Drosha, and β-actin in DRCR8-knockdown LM2 (**g**), LM2-R (**h**), and LM2-DRR (**i**) cells with ectopic expression of wild-type (WT) DGCR8, the S677A mutant, or the S677D mutant.

(**j**) Relative transfection efficiency of the cells used in **Fig. 7e**, gauged by an exogenous donor (pCAGGS DRR mCherry Donor EF1a BFP) that expresses BFP. *n* = 3 biological replicates. Error bars are mean ± s.e.m.

(**k**) Clonogenic survival assays of DRCR8-knockdown LM2 cells with ectopic expression of wild-type (WT) DGCR8, the S677A mutant, or the S677D mutant after X-ray IR treatment. *n* = 3 wells per group.

(**l**) Left panel: immunoblotting of DGCR8, Drosha, and β-actin in DGCR8-knockdown BT549 cells with ectopic expression of wild-type (WT) DGCR8, the S677A mutant, or the S677D mutant. Right panel: clonogenic survival assays after X-ray IR treatment. *n* = 3 wells per group.

Statistical significance in **j-l** was determined by a two-tailed unpaired *t*-test. Error bars are mean ± s.e.m. *n.s.*: not statistically significant. Source data are provided as a Source Data file.

Supplementary Figure 9

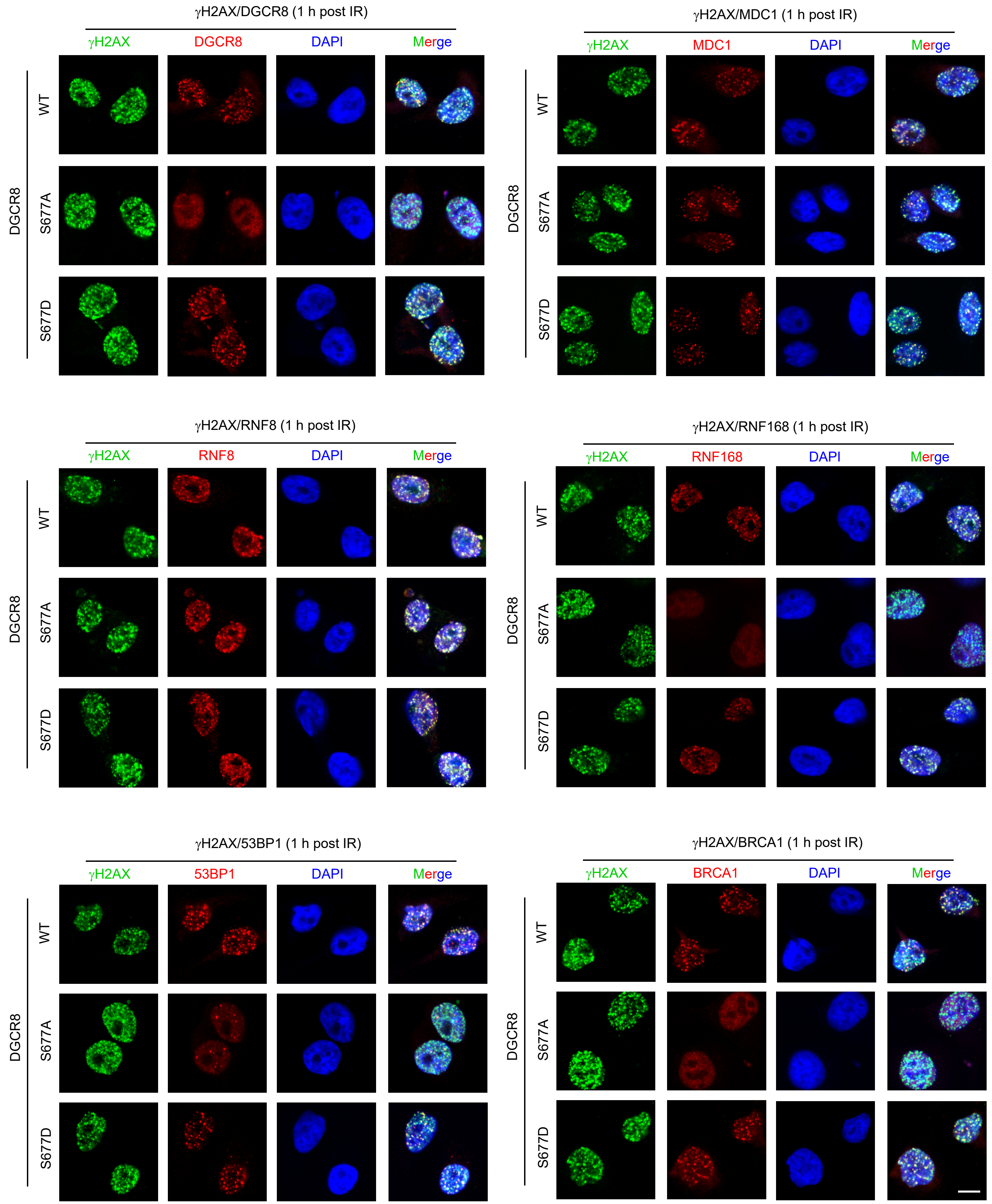

Supplementary Figure 9. Representative images of γH2AX, DGCR8, MDC1, RNF8, RNF168, 53BP1, and BRCA1 foci in DRCR8-knockdown LM2 cells with ectopic expression of wild-type (WT) DGCR8, S677A-DGCR8, or S677D-DGCR8.
Cells were incubated for 1 hour after 2-Gy IR and immunostained with antibodies against γH2AX, DGCR8, MDC1, RNF8, RNF168, 53BP1, and BRCA1 (see data quantification in **Fig. 8d**). Scale bar, 10 μm.

Supplementary Figure 10

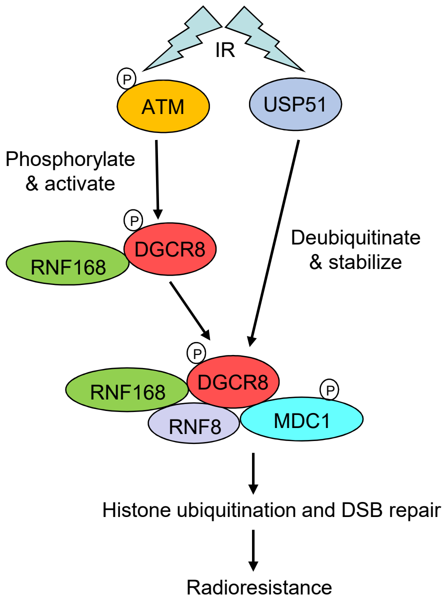

Supplementary Figure 10. Working model for the role of DGCR8 in regulating DSB repair signaling and tumor radioresistance.

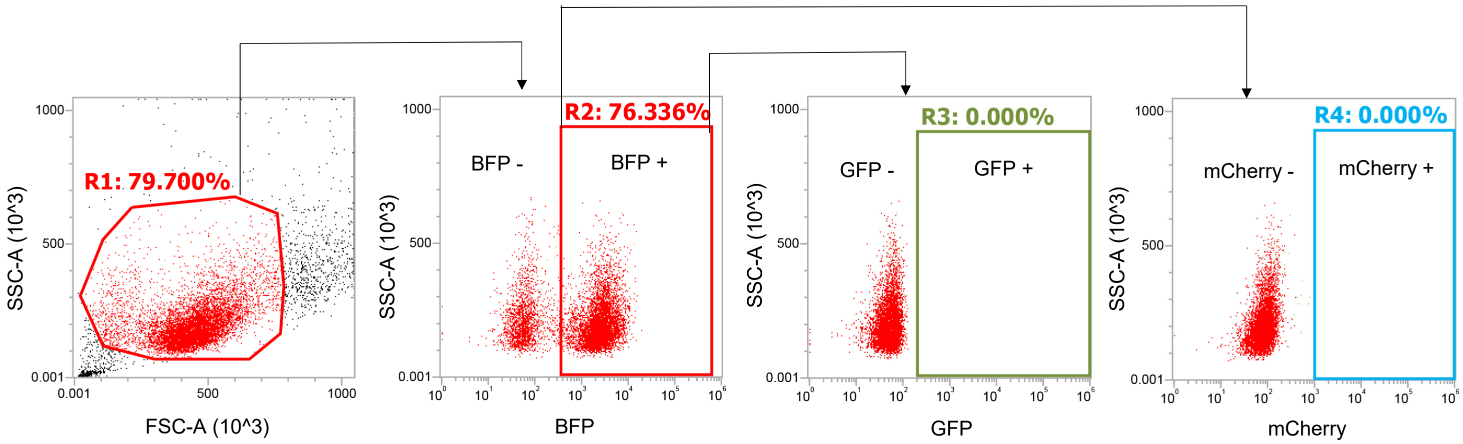

Supplementary Figure 11. An example of the gating strategy for HR and NHEJ repair analysis through flow cytometry.

Initial cell population gating (SSC-Area vs FSC-Area) was adopted to ensure that only single cells were used for analysis.

Supplementary Figure 12

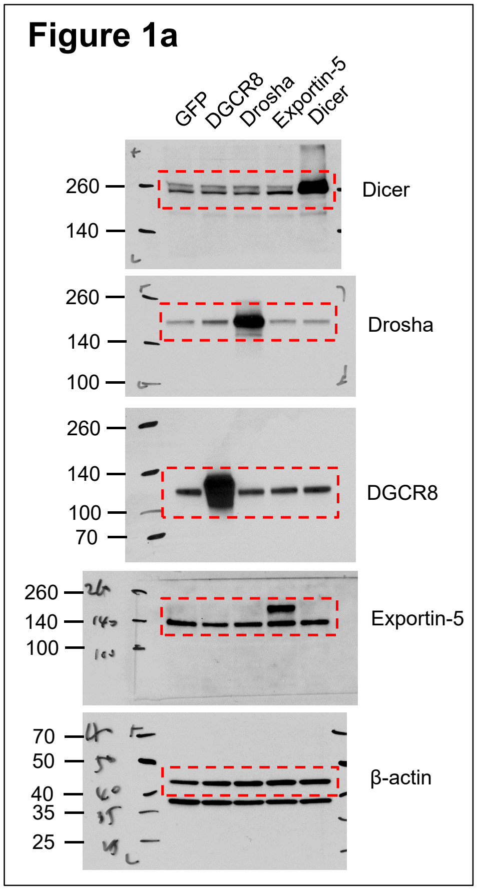

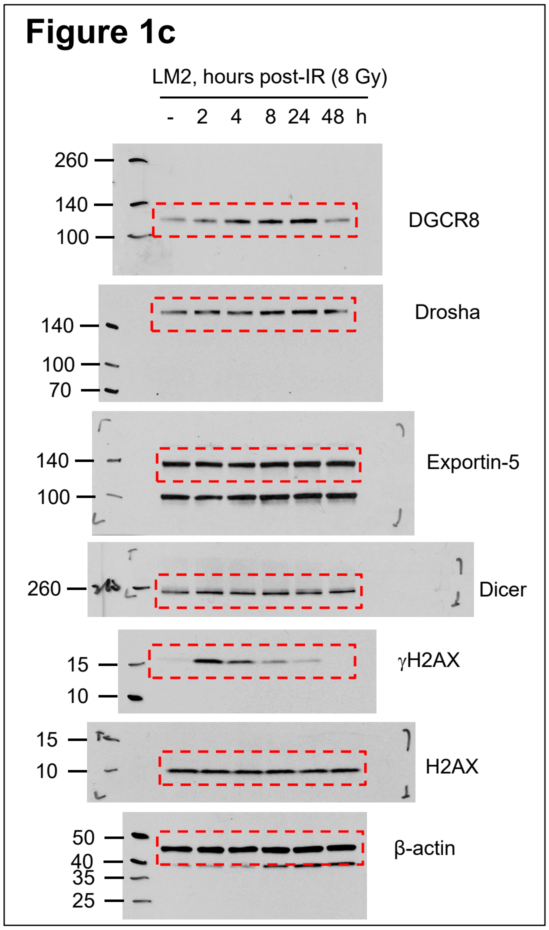

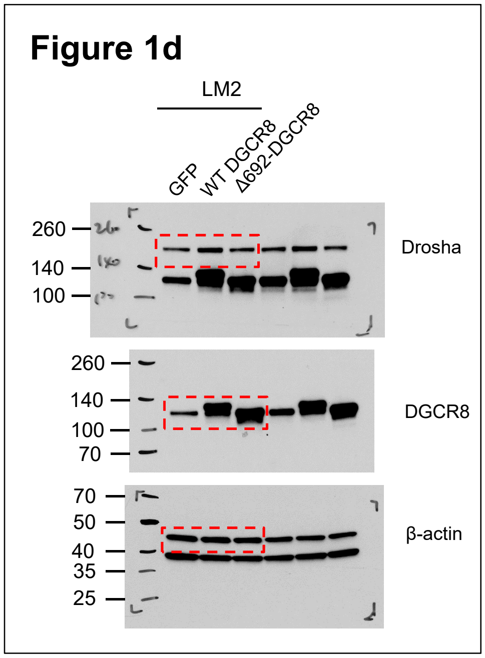

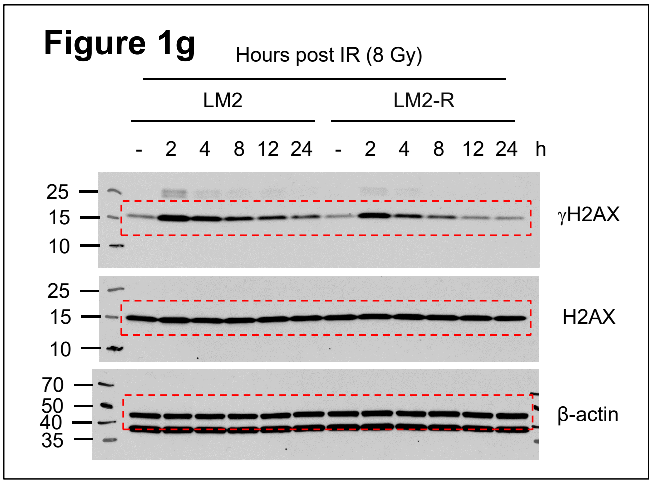

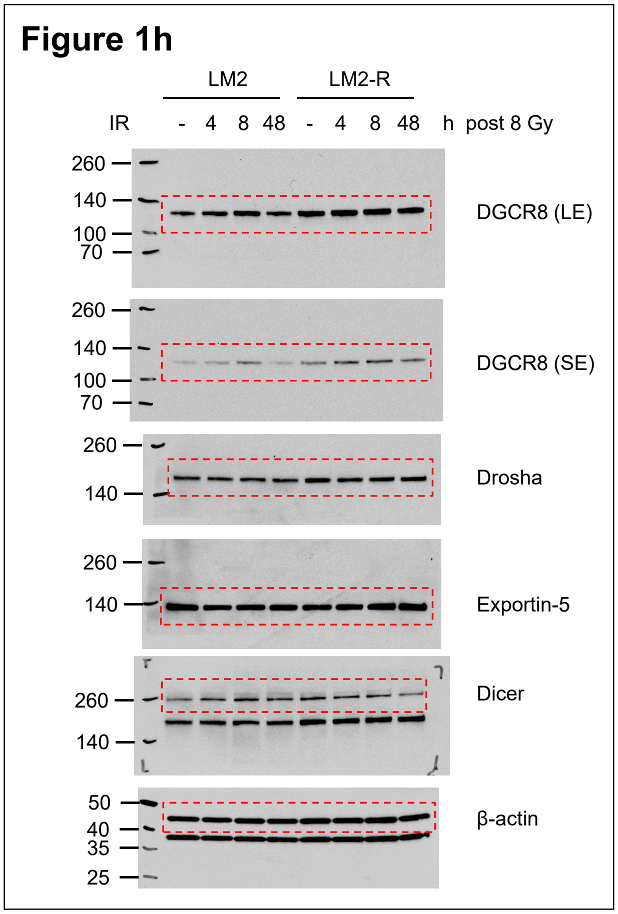

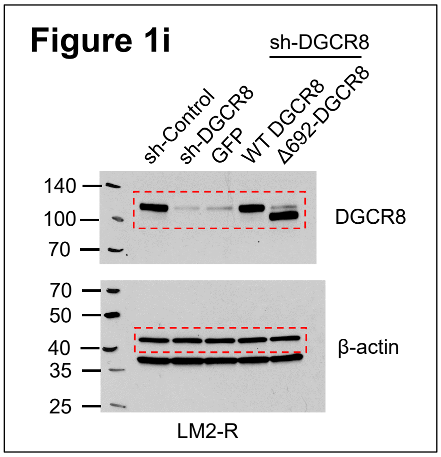

Supplementary Figure 12. All uncropped blots and gels in this manuscript.

Supplementary Figure 12

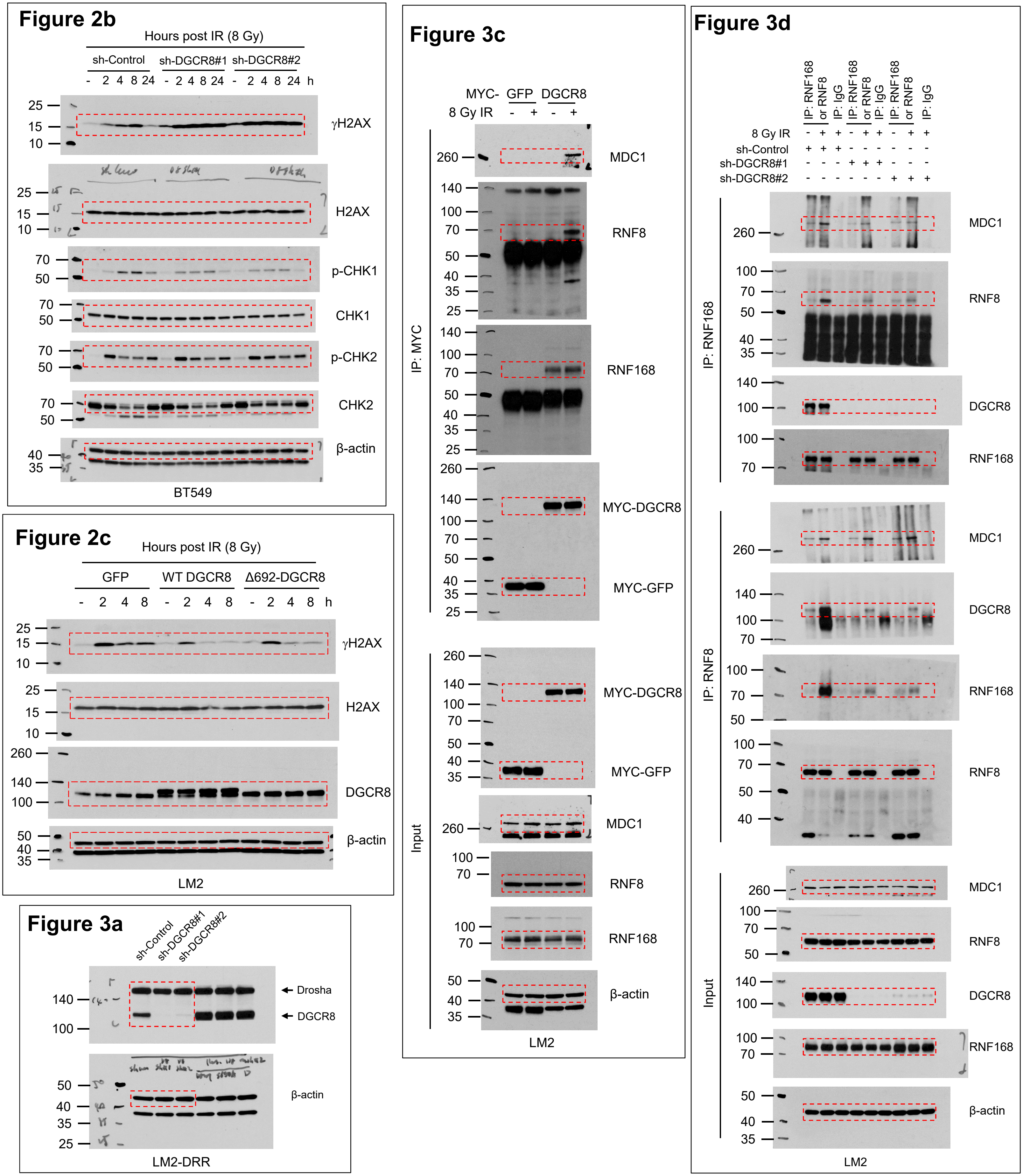

Supplementary Figure 12. All uncropped blots and gels in this manuscript.

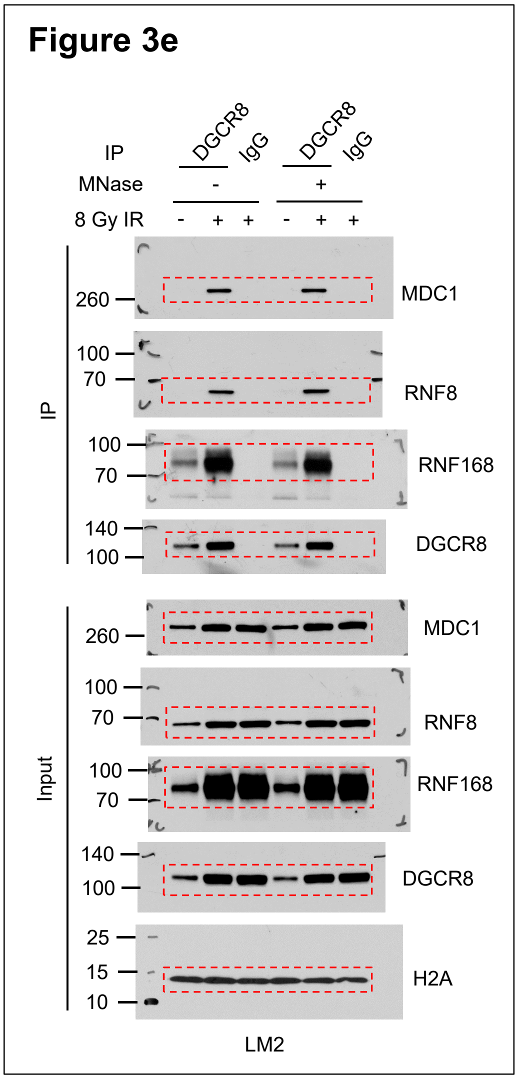

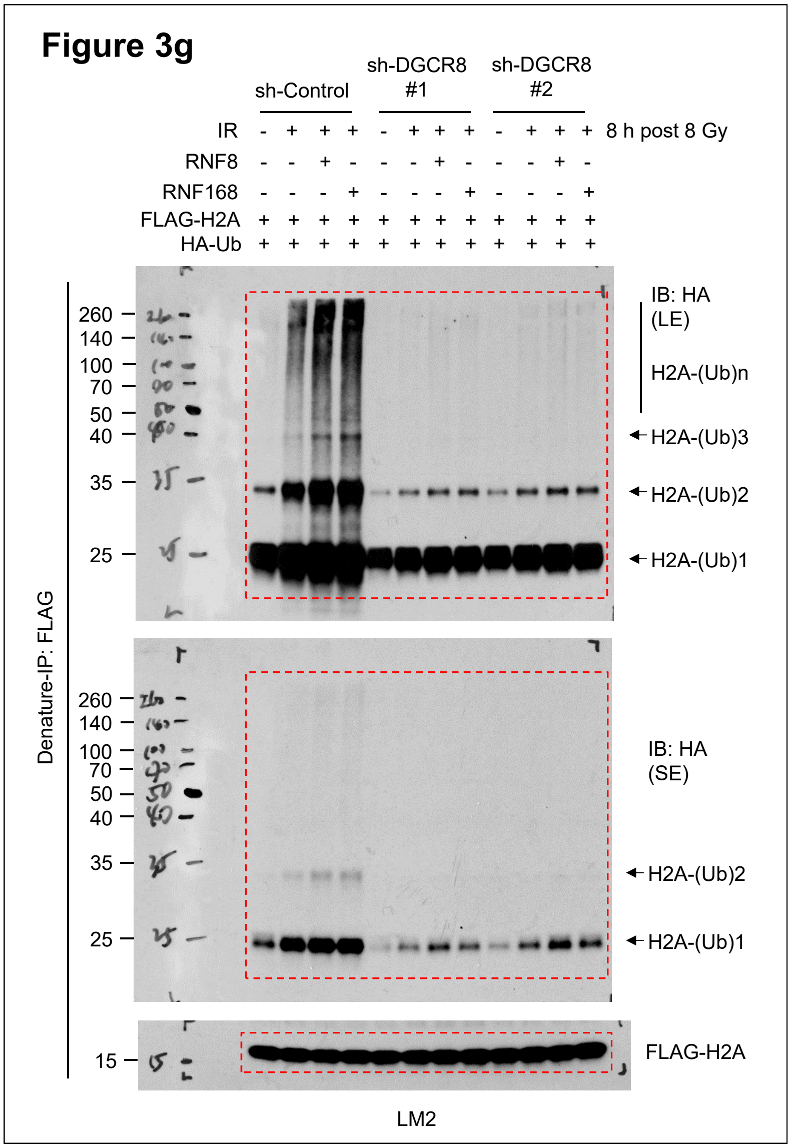

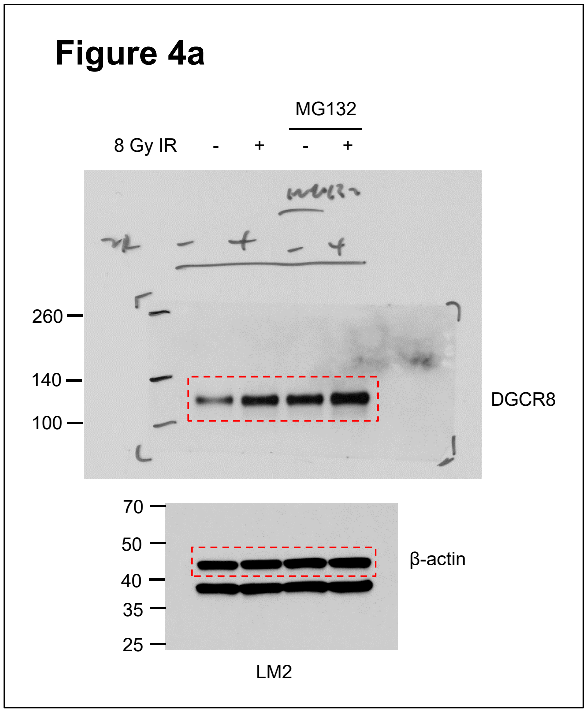

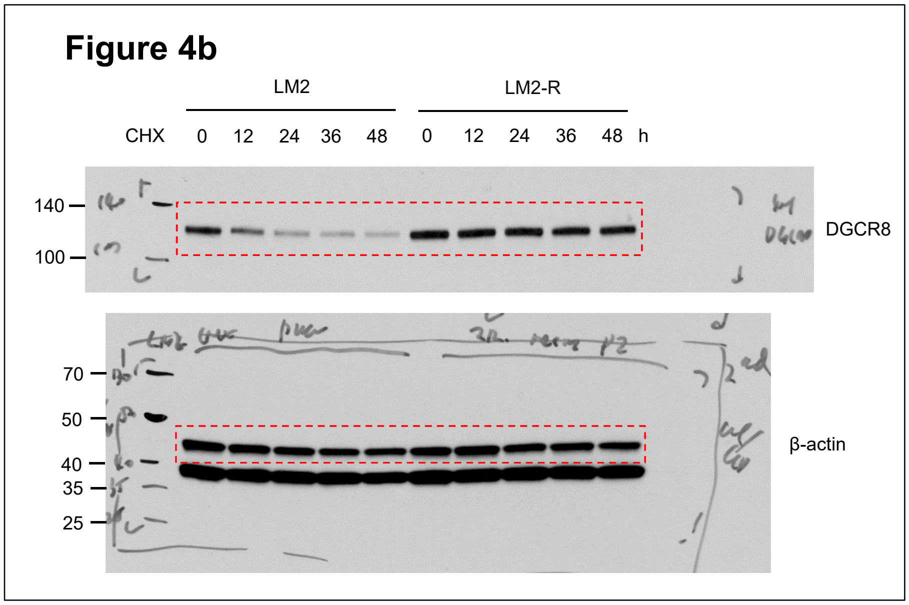

Supplementary Figure 12. All uncropped blots and gels in this manuscript.

Supplementary Figure 12

Figure 4d

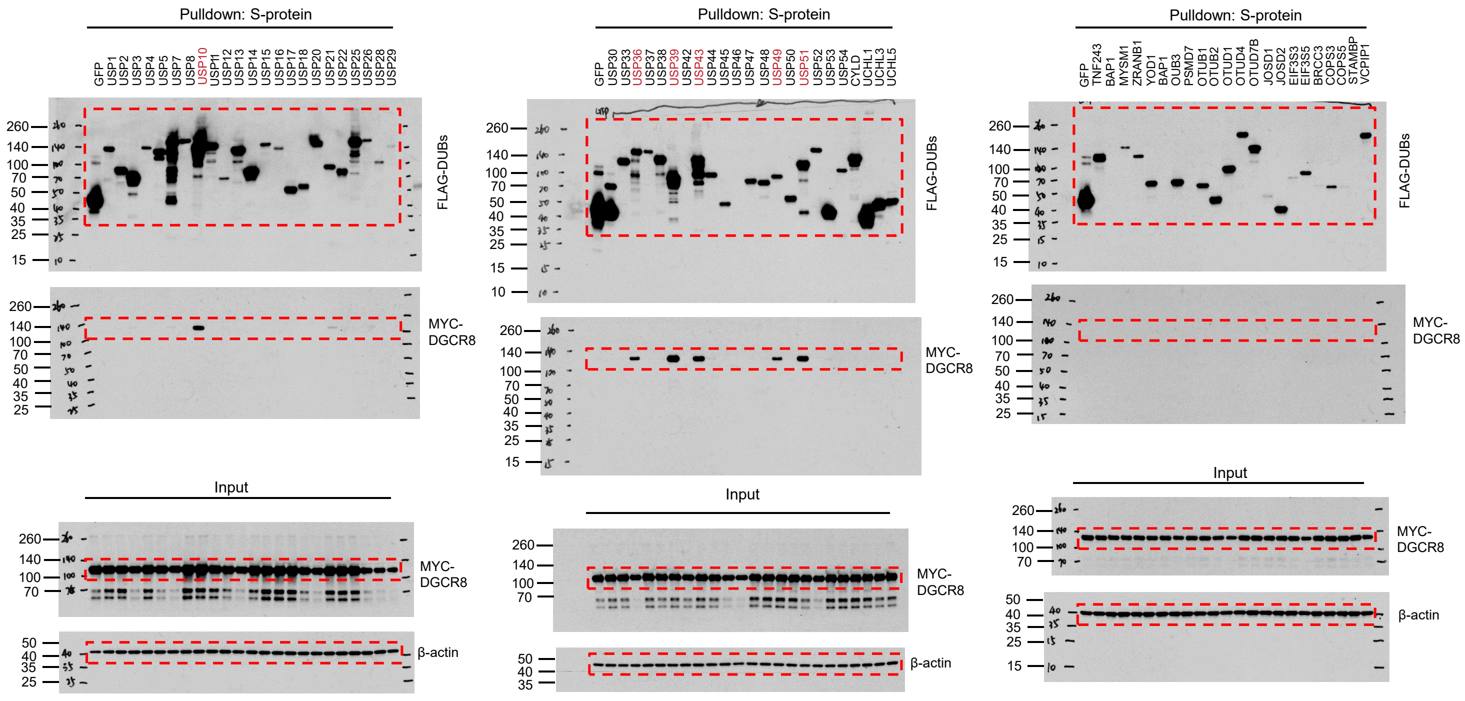

Figure 4e

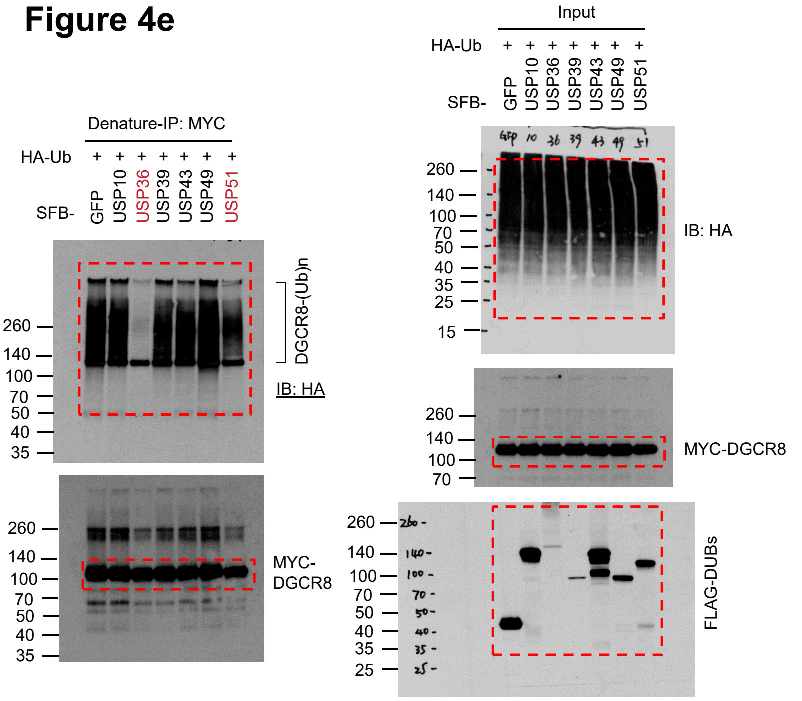

Figure 4f

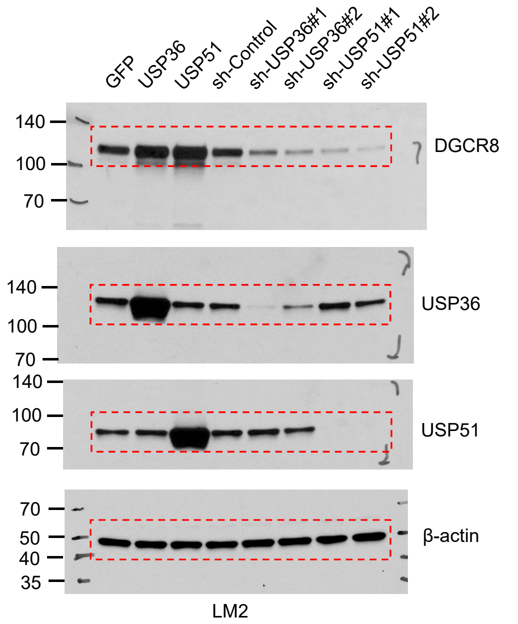

Supplementary Figure 12. All uncropped blots and gels in this manuscript.

Supplementary Figure 12

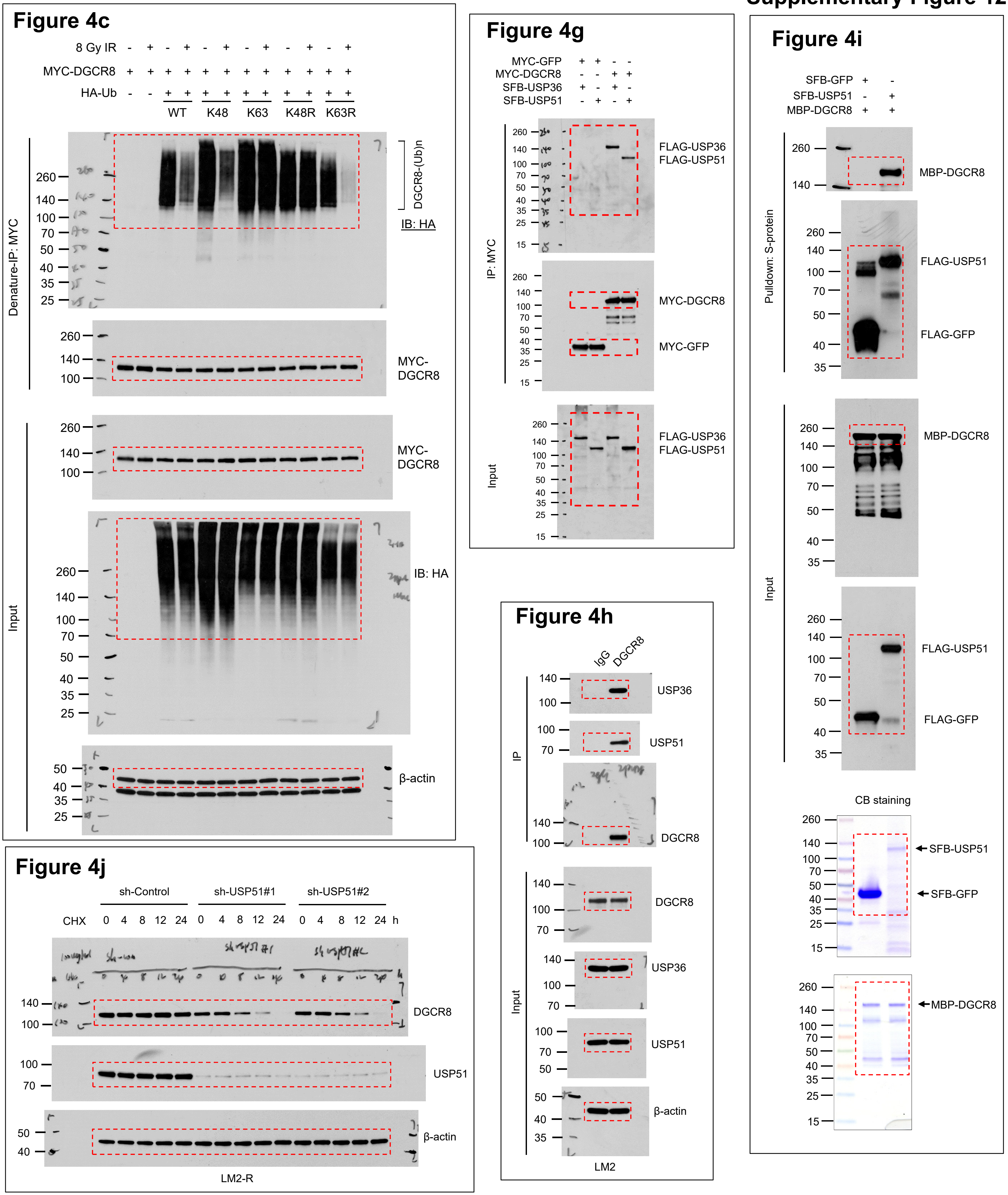

Supplementary Figure 12. All uncropped blots and gels in this manuscript.

Figure 5a

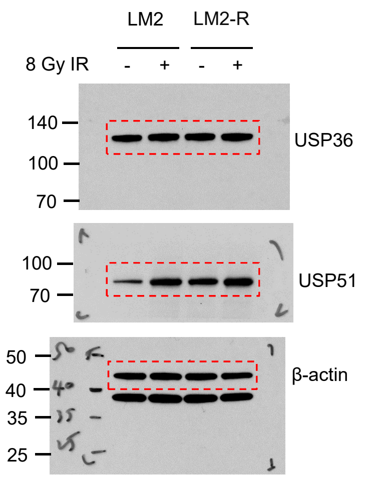

Figure 5b

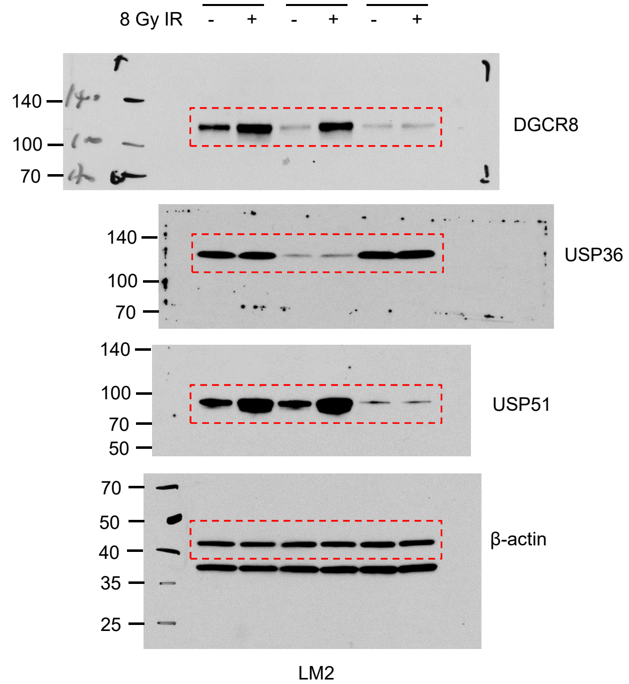

Figure 5g

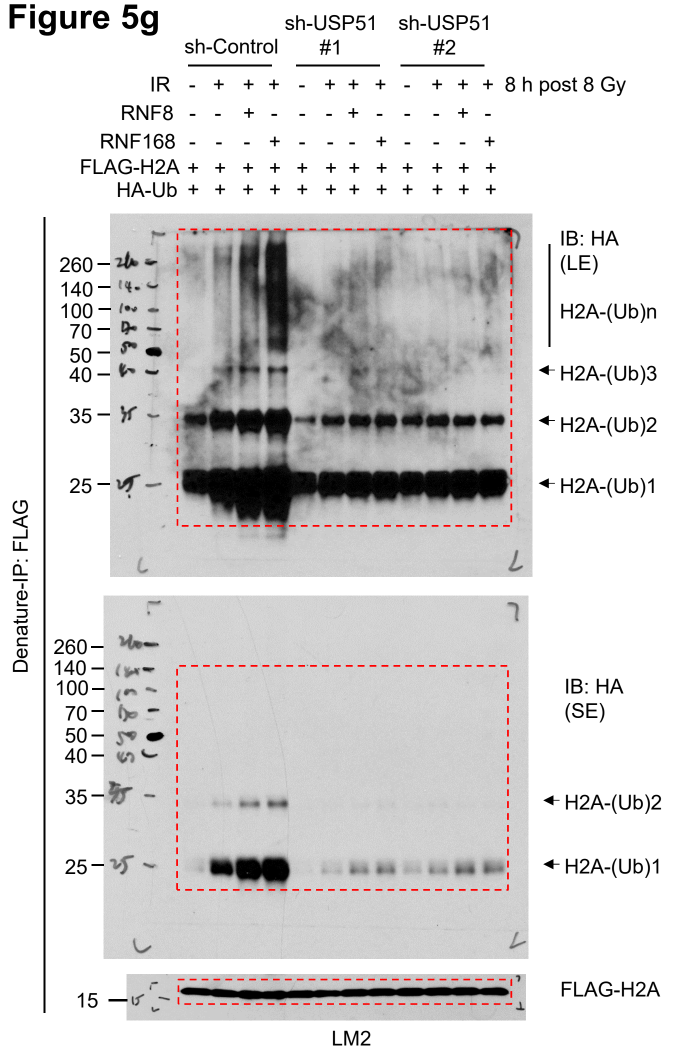

Figure 5c

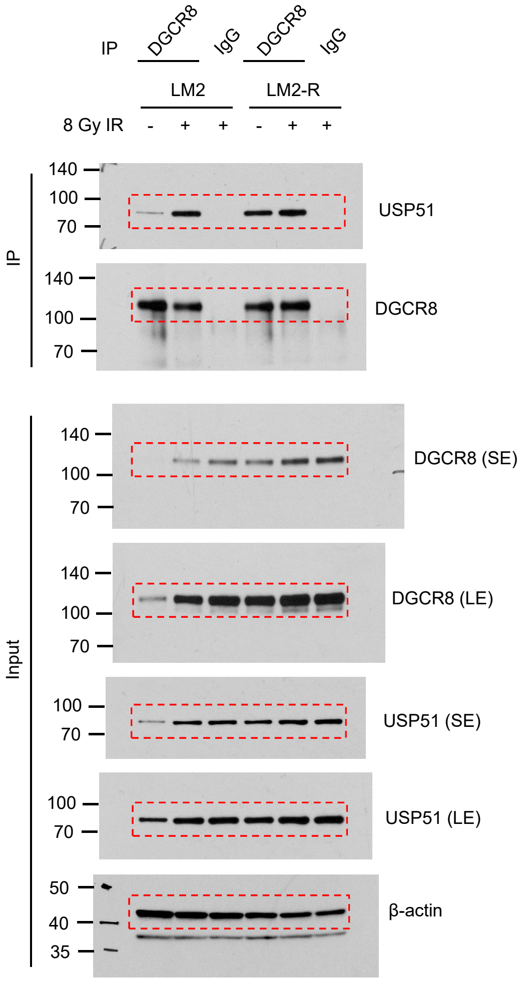

Supplementary Figure 12. All uncropped blots and gels in this manuscript.

Figure 5d

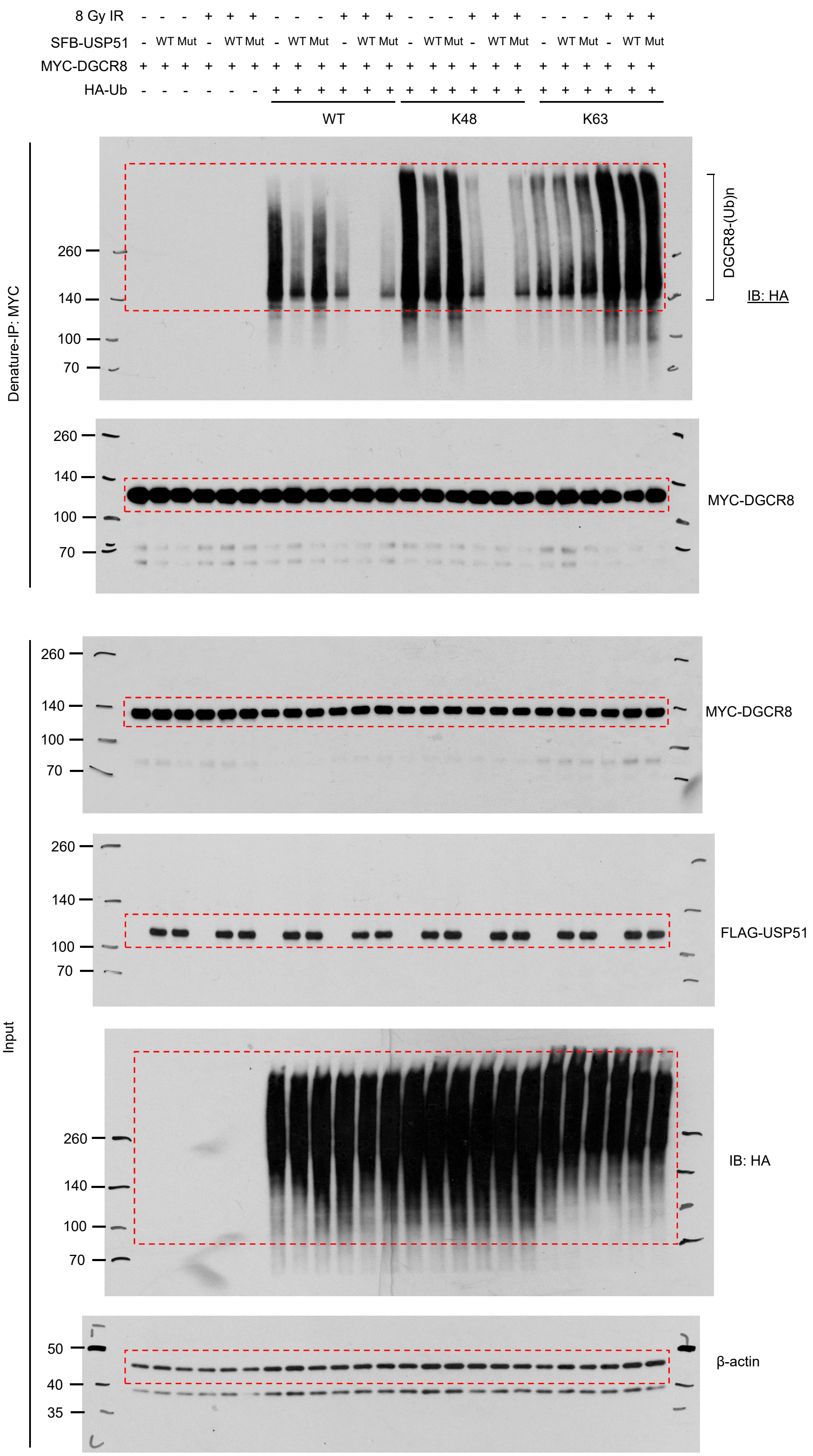

Figure 6b

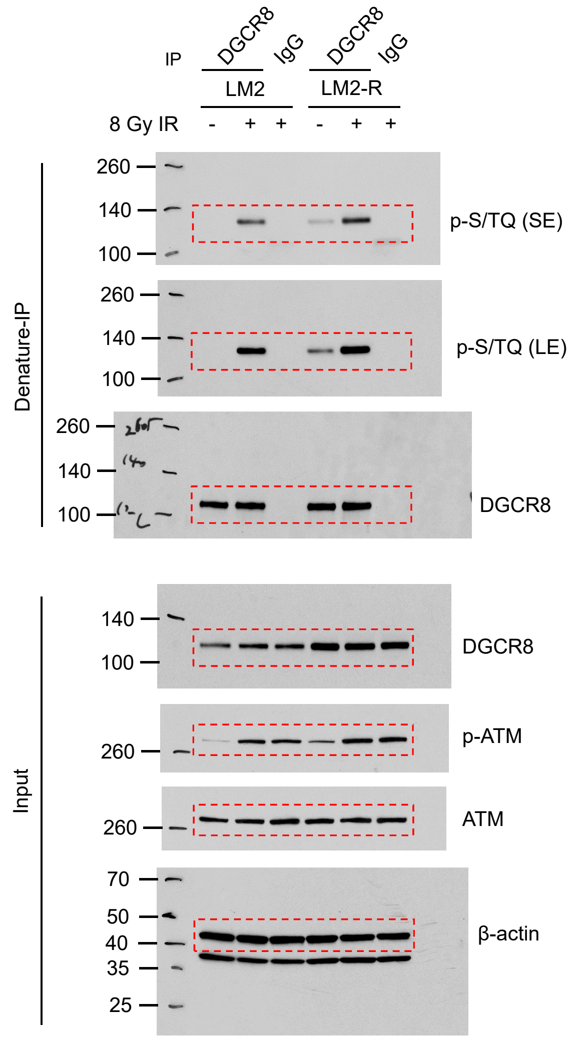

Supplementary Figure 12. All uncropped blots and gels in this manuscript.

Supplementary Figure 12

Figure 6a

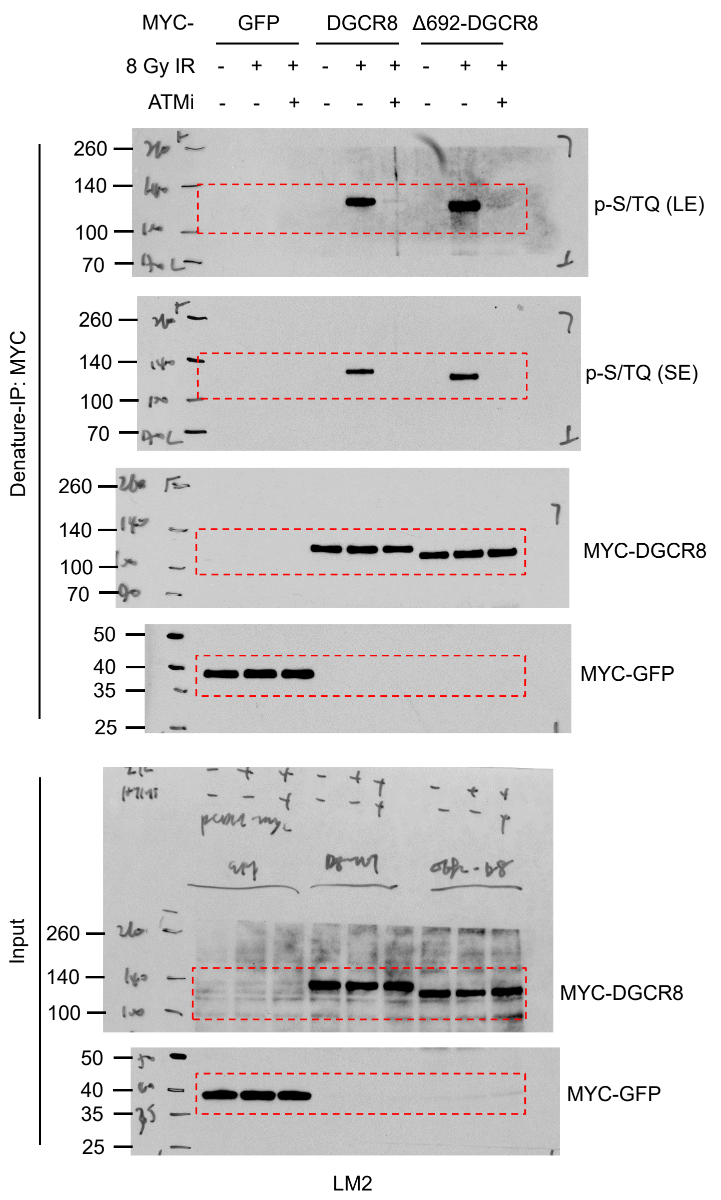

Figure 6g

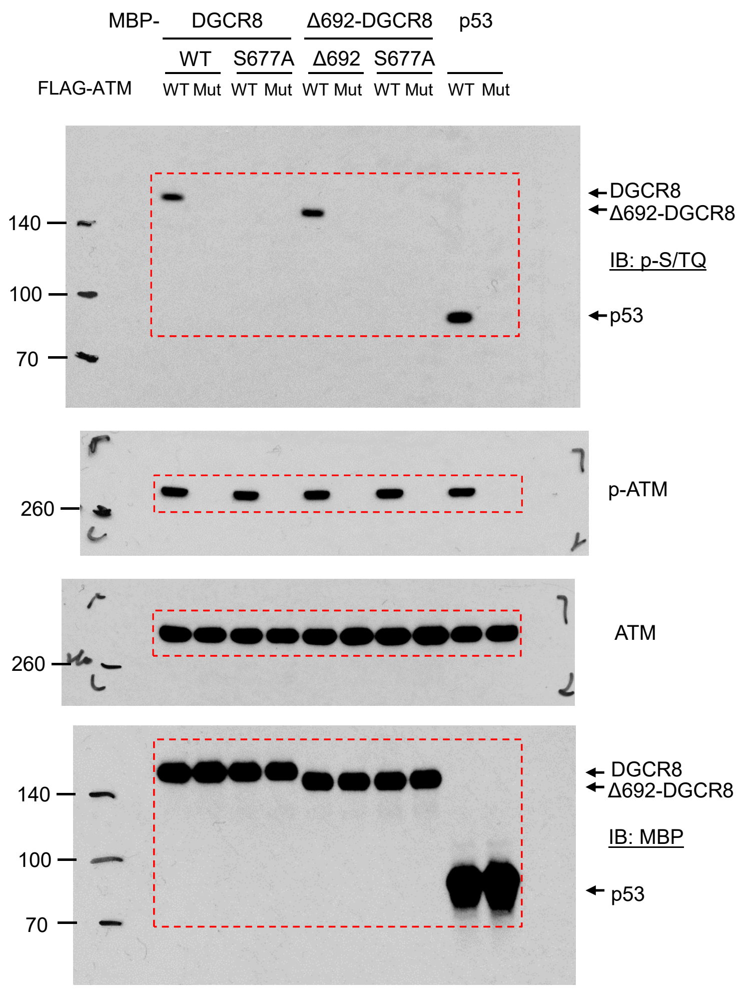

Supplementary Figure 12. All uncropped blots and gels in this manuscript.

Supplementary Figure 12

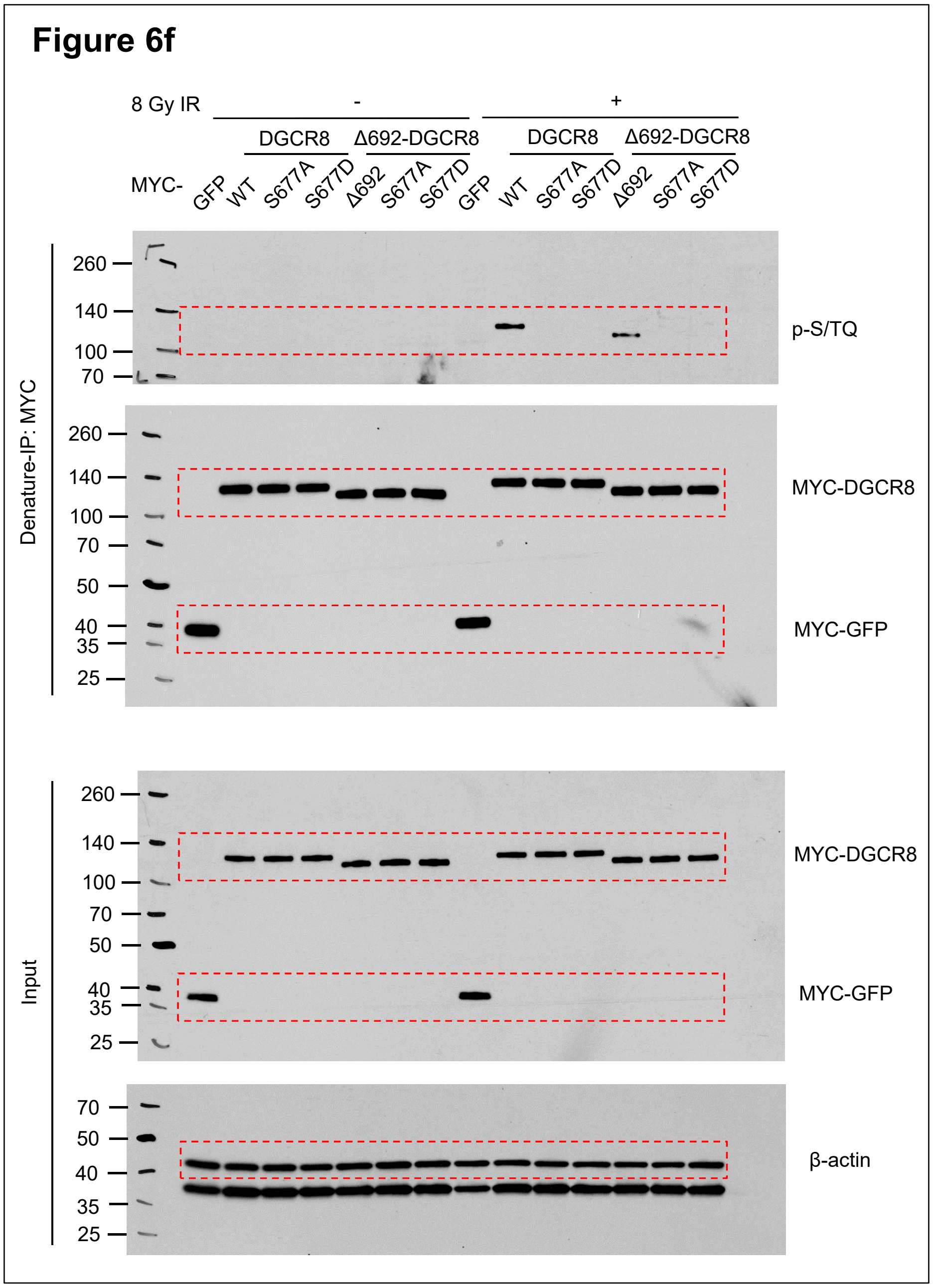

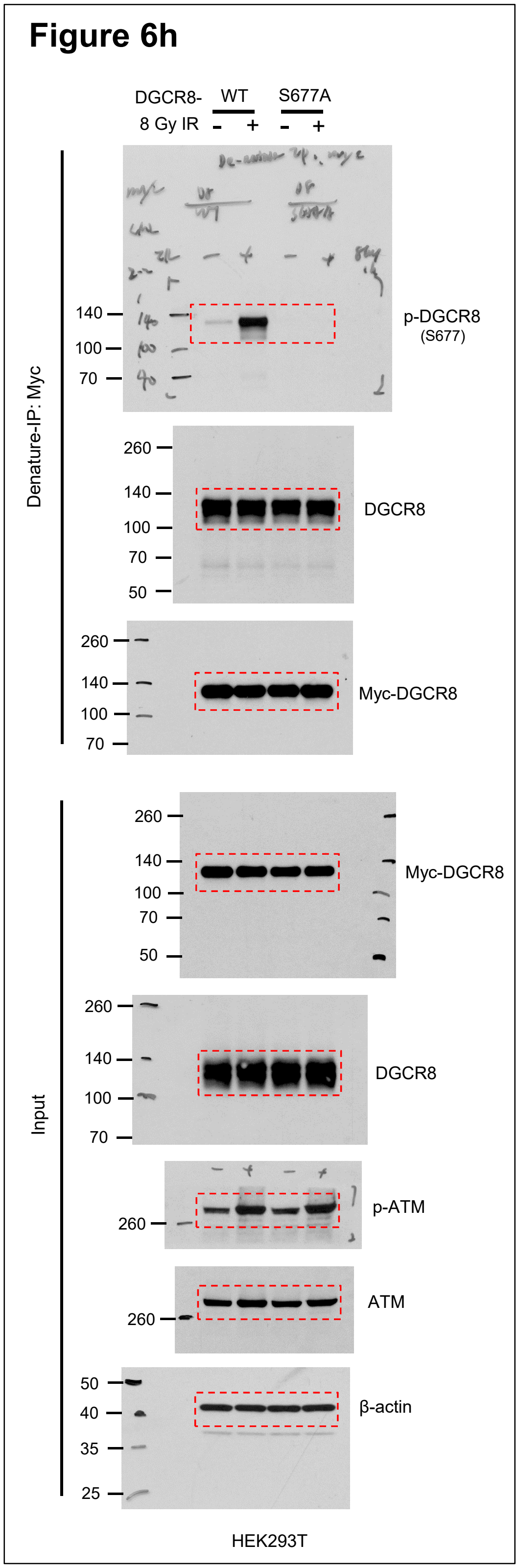

Supplementary Figure 12. All uncropped blots and gels in this manuscript.

Supplementary Figure 12

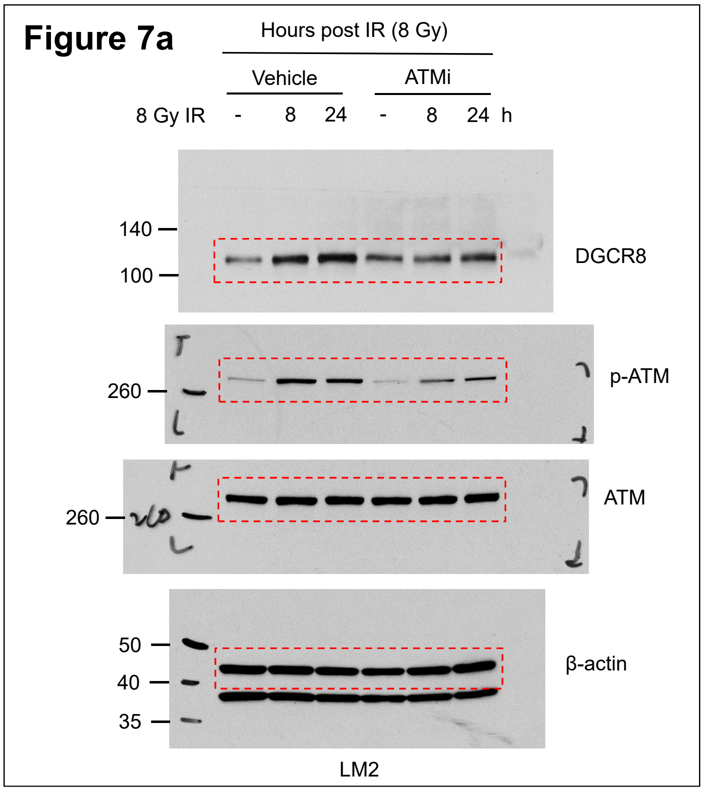

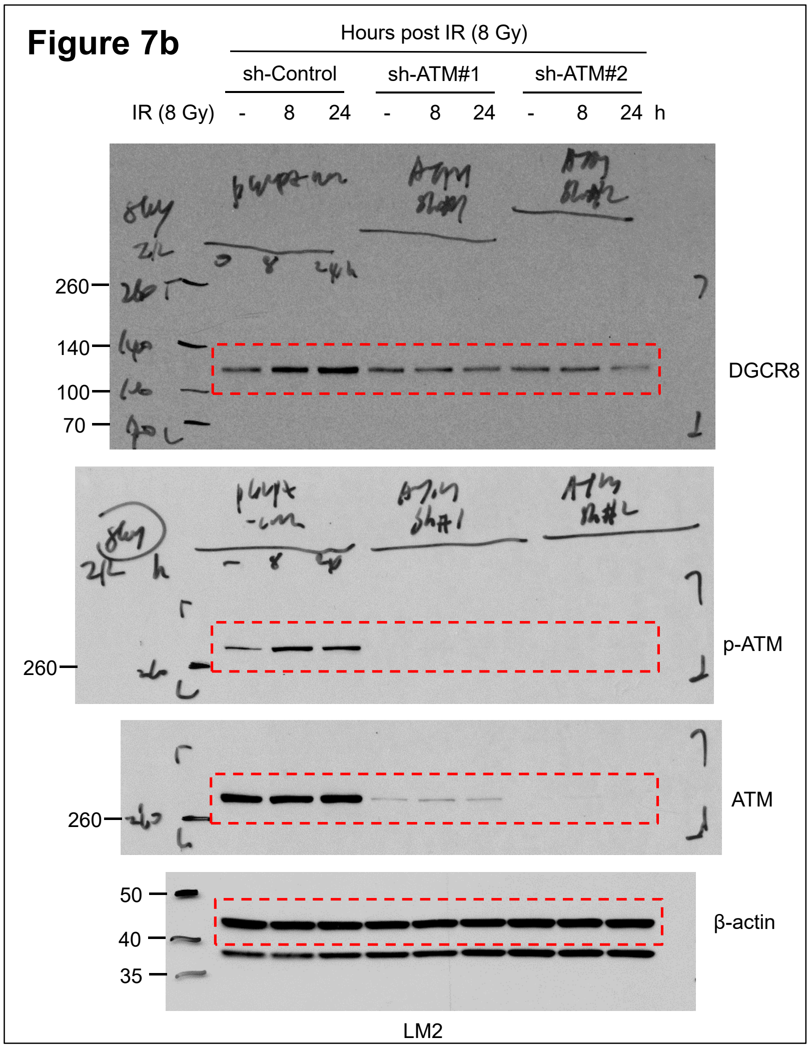

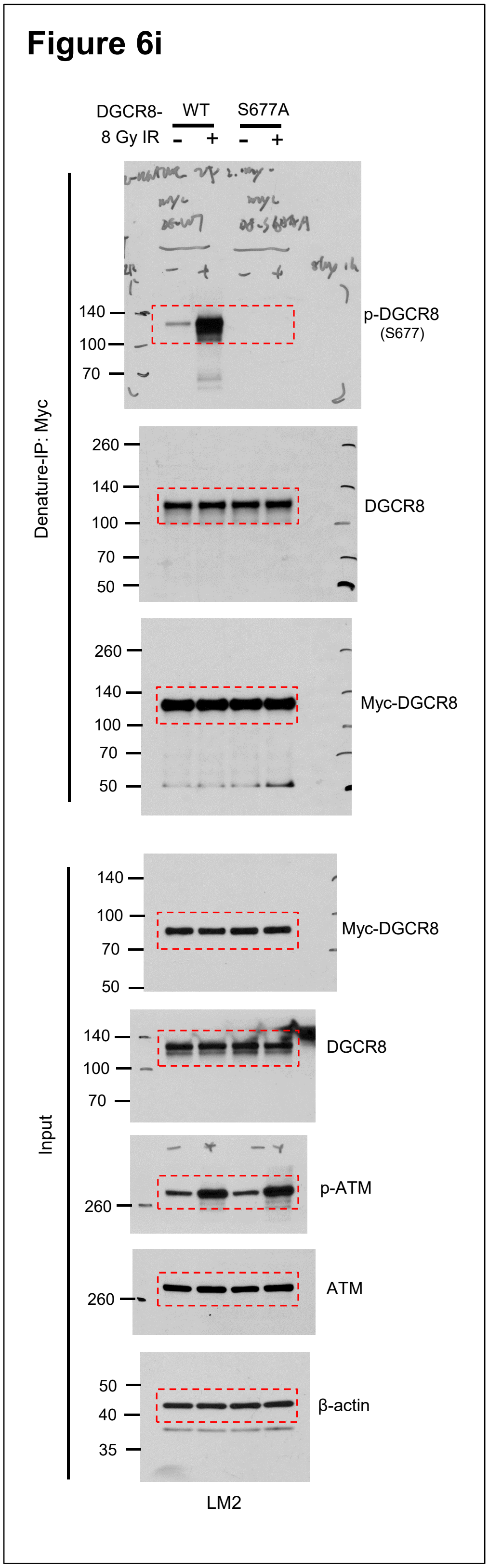

Supplementary Figure 12. All uncropped blots and gels in this manuscript.

Figure 7c

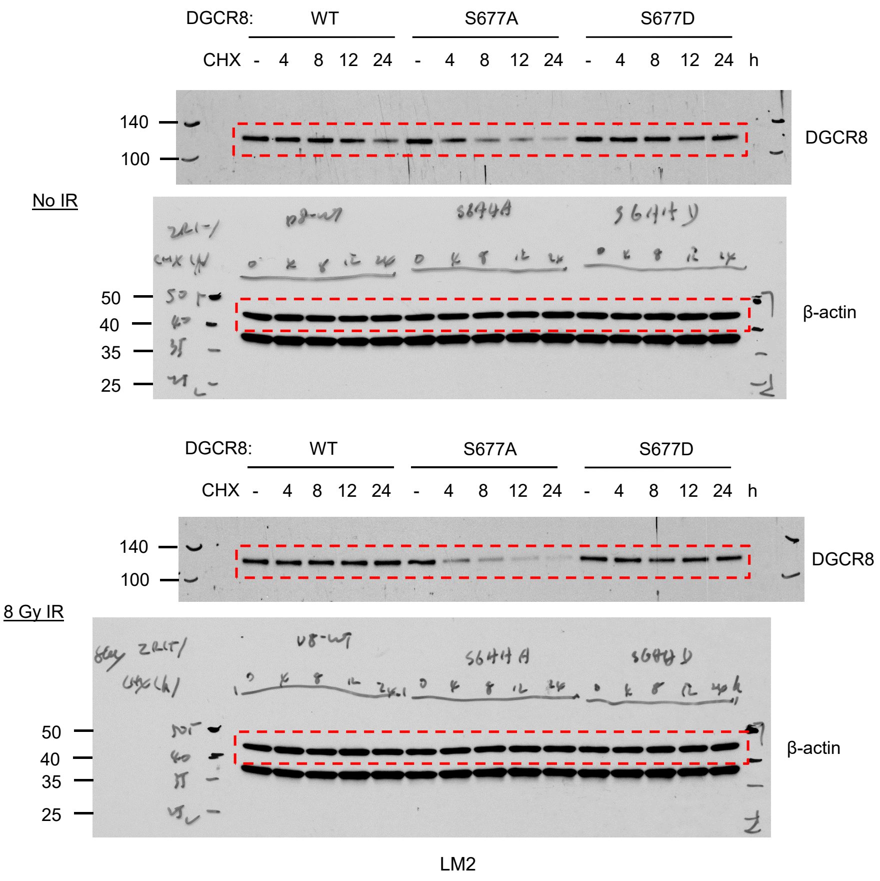

Figure 7d

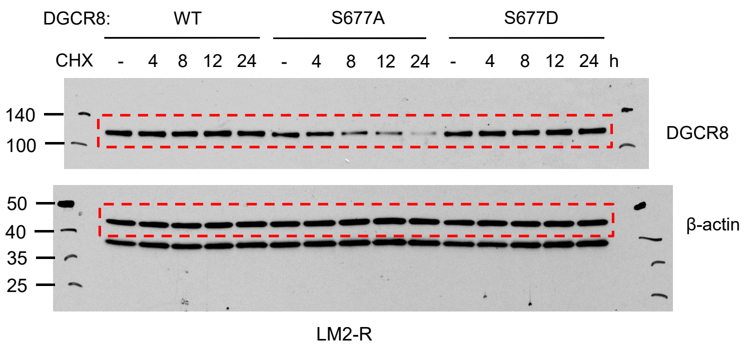

Figure 8a

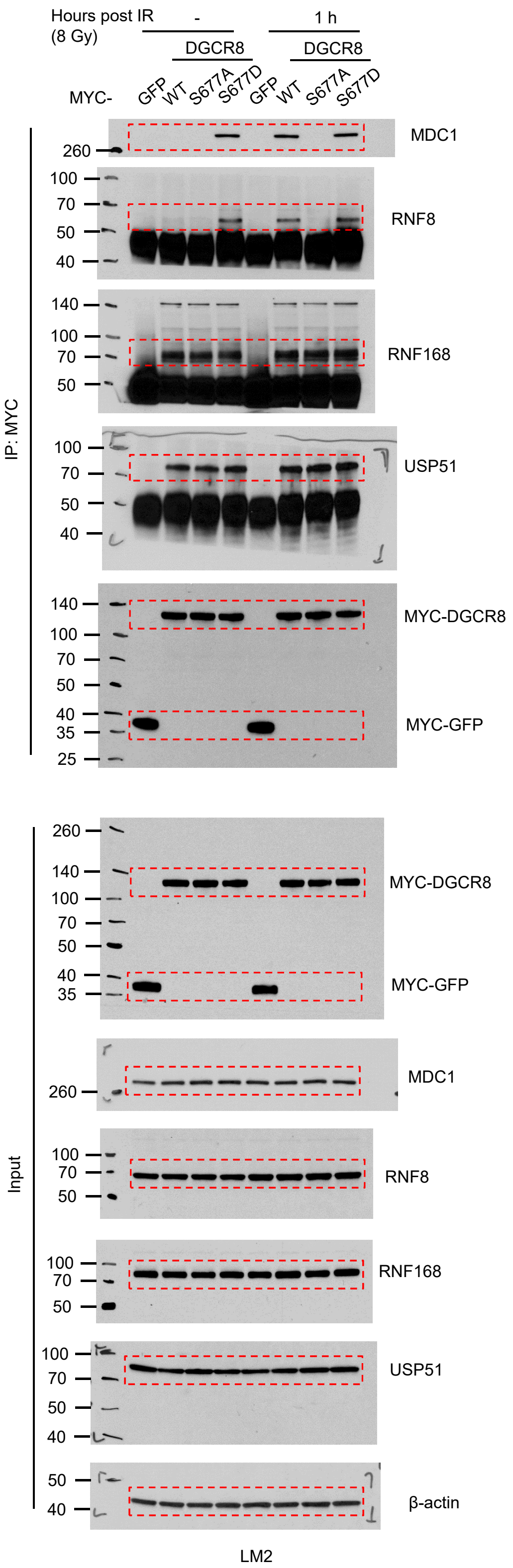

Supplementary Figure 12. All uncropped blots and gels in this manuscript.

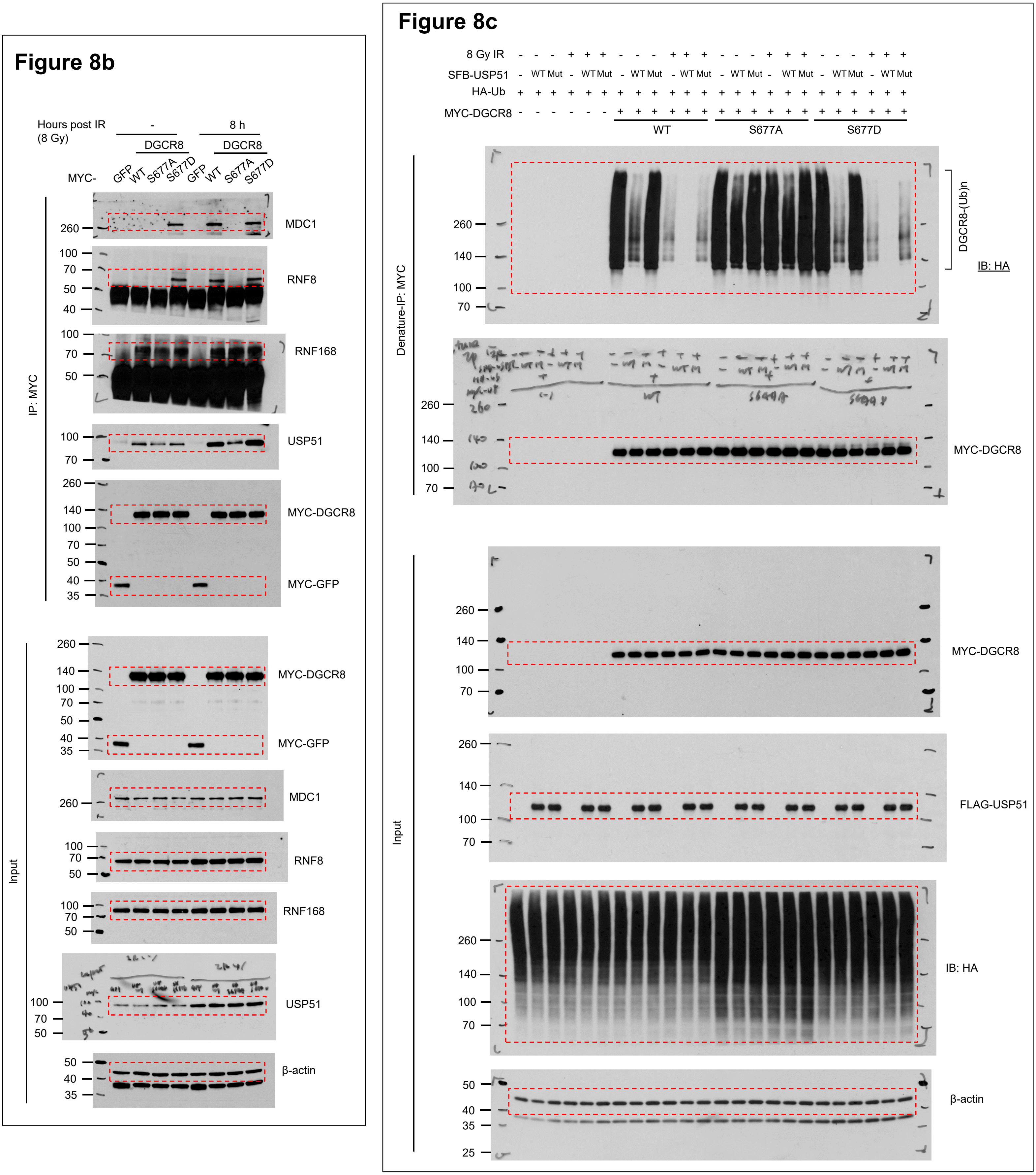

Supplementary Figure 12. All uncropped blots and gels in this manuscript.

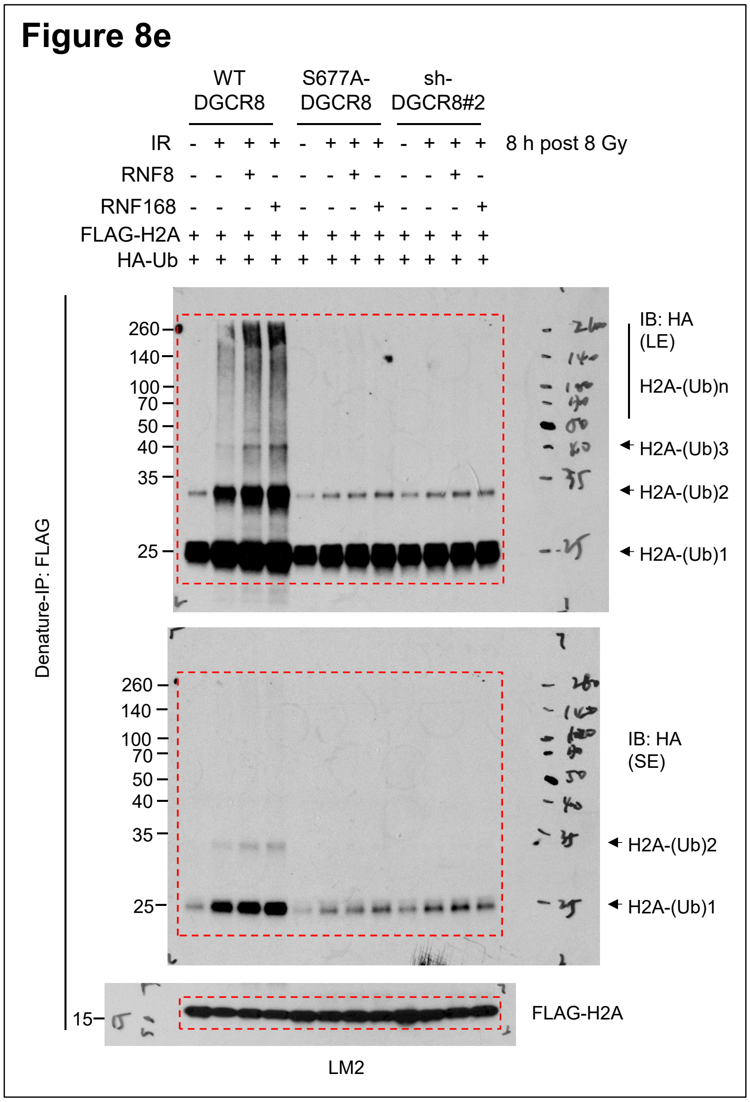

Supplementary Figure 12. All uncropped blots and gels in this manuscript.

Supplementary Figure 1a

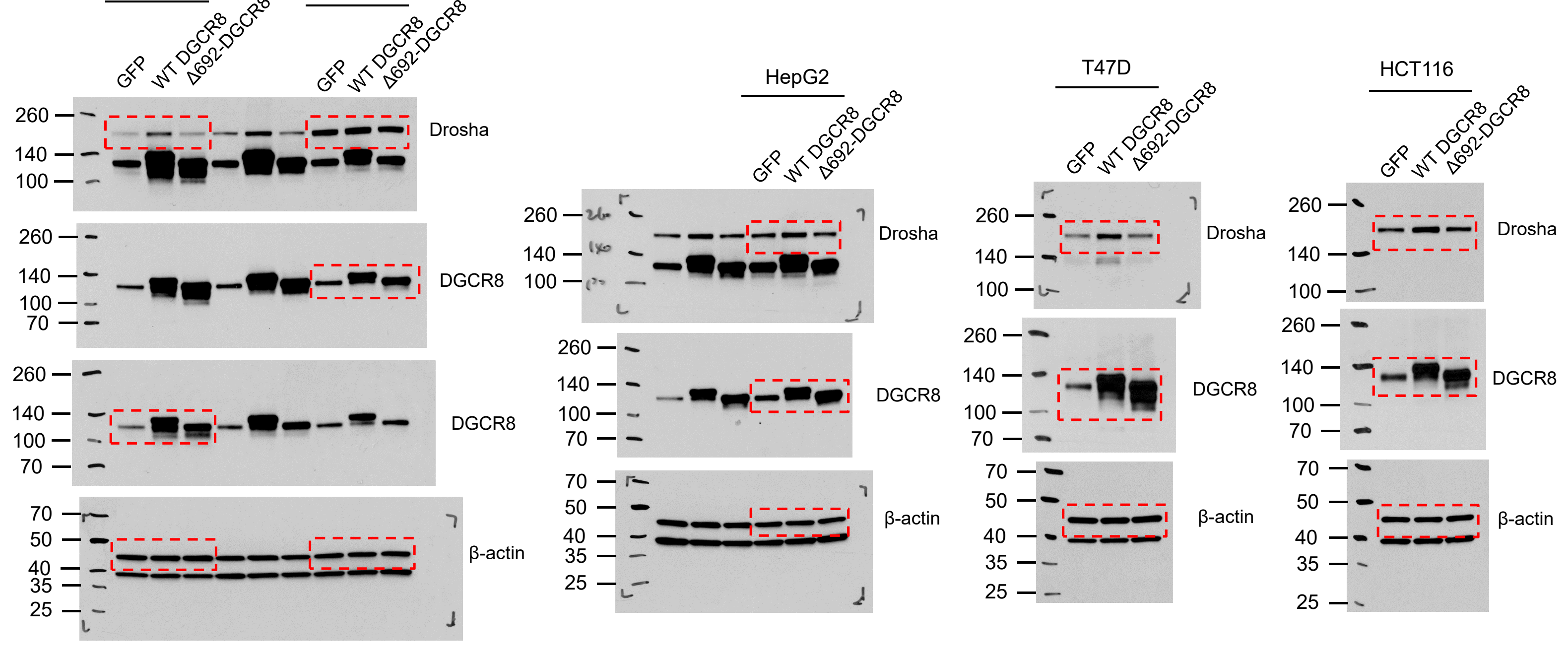

Supplementary Figure 1b

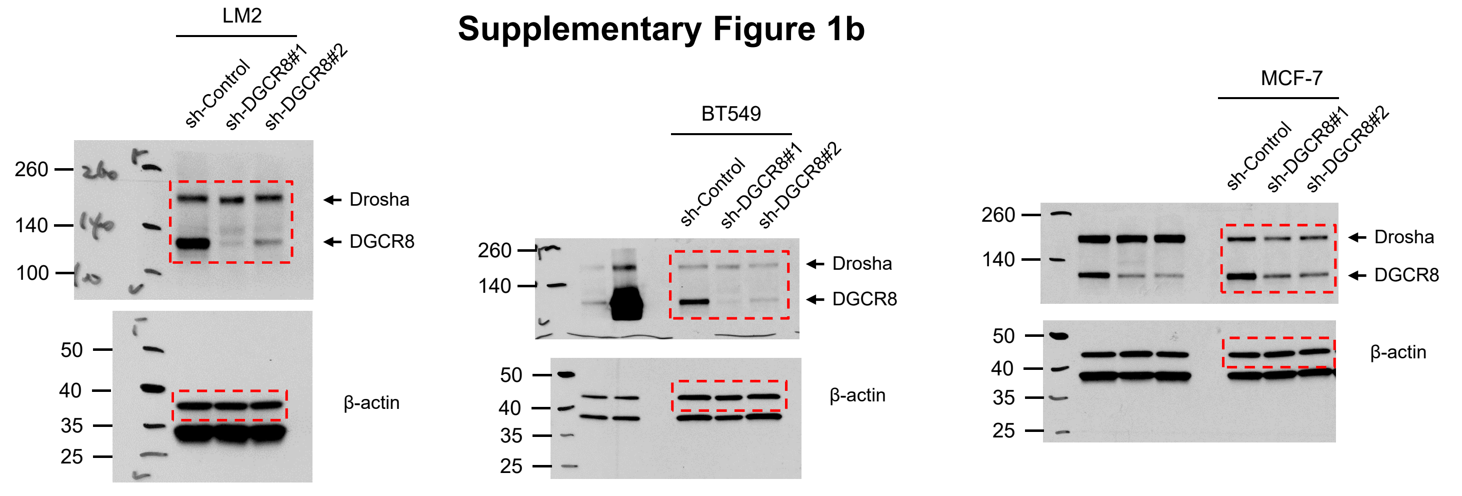

Supplementary Figure 1c

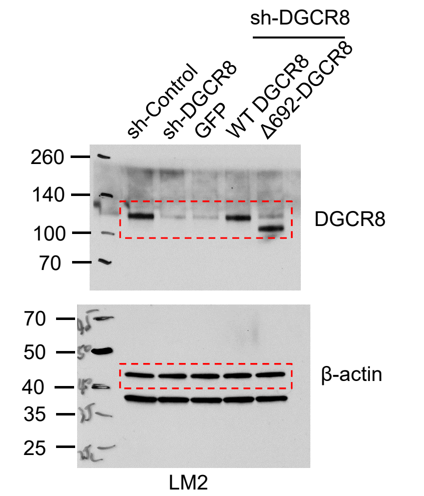

Supplementary Figure 1d

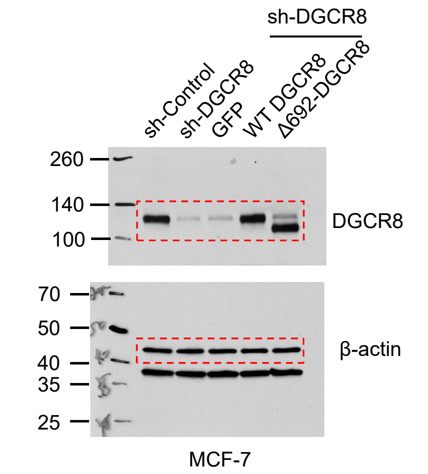

Supplementary Figure 1e

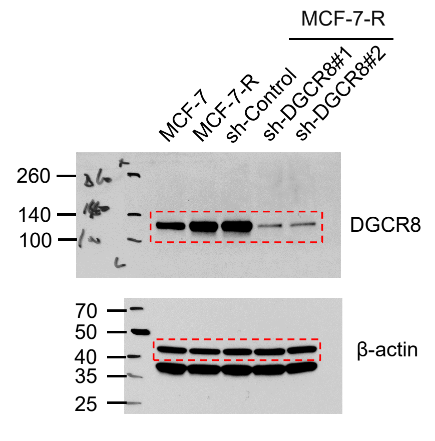

Supplementary Figure 12. All uncropped blots and gels in this manuscript.

Supplementary Figure 12

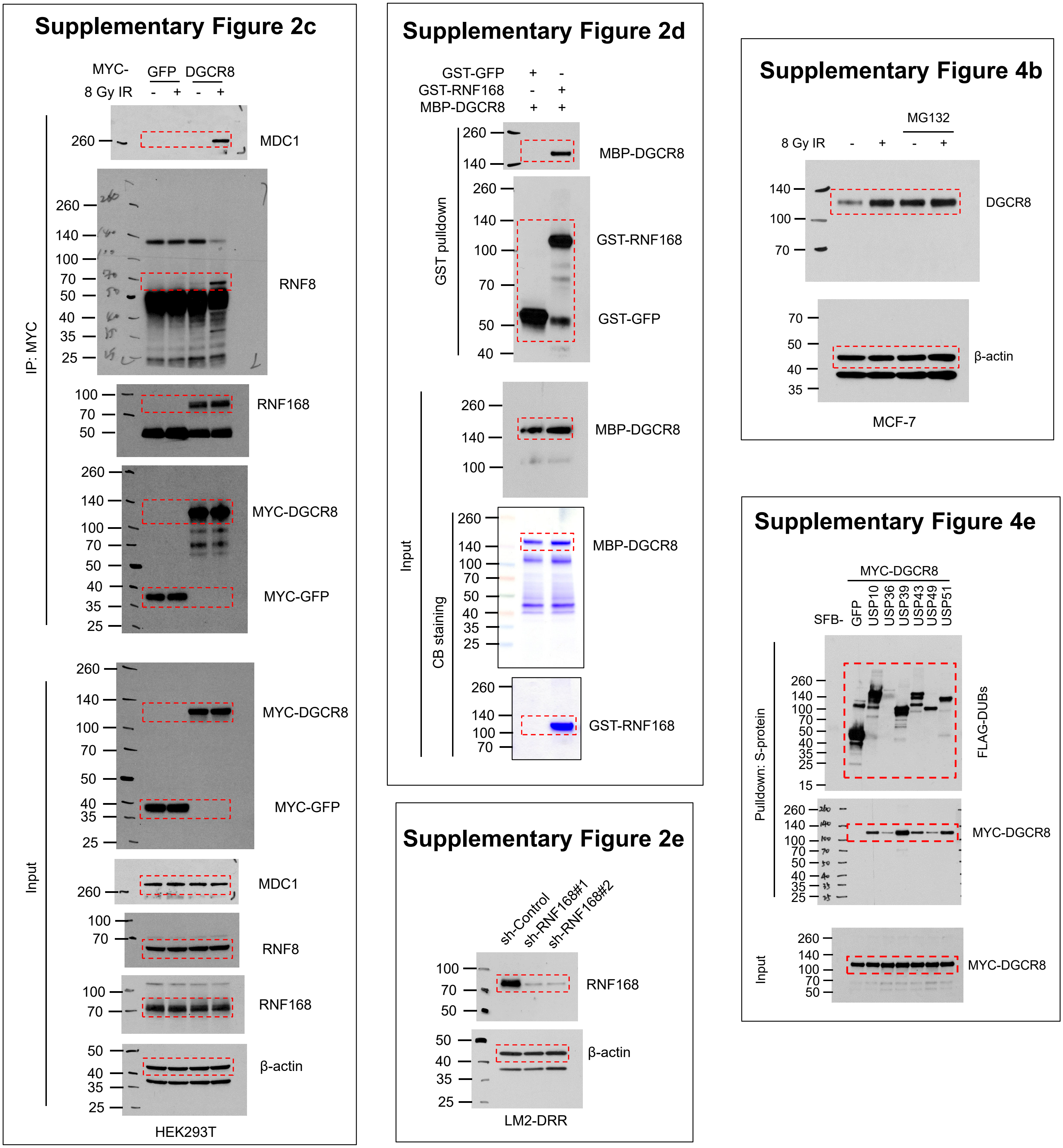

Supplementary Figure 12. All uncropped blots and gels in this manuscript.

Supplementary Figure 12

Supplementary Figure 4d

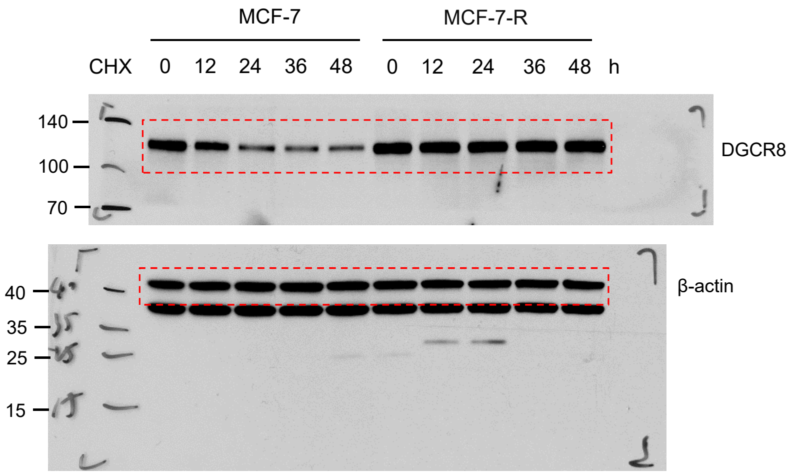

Supplementary Figure 4f

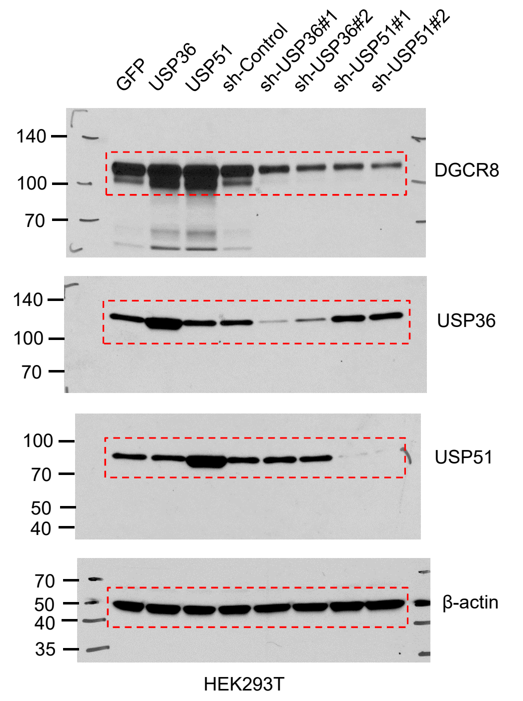

Supplementary Figure 4i

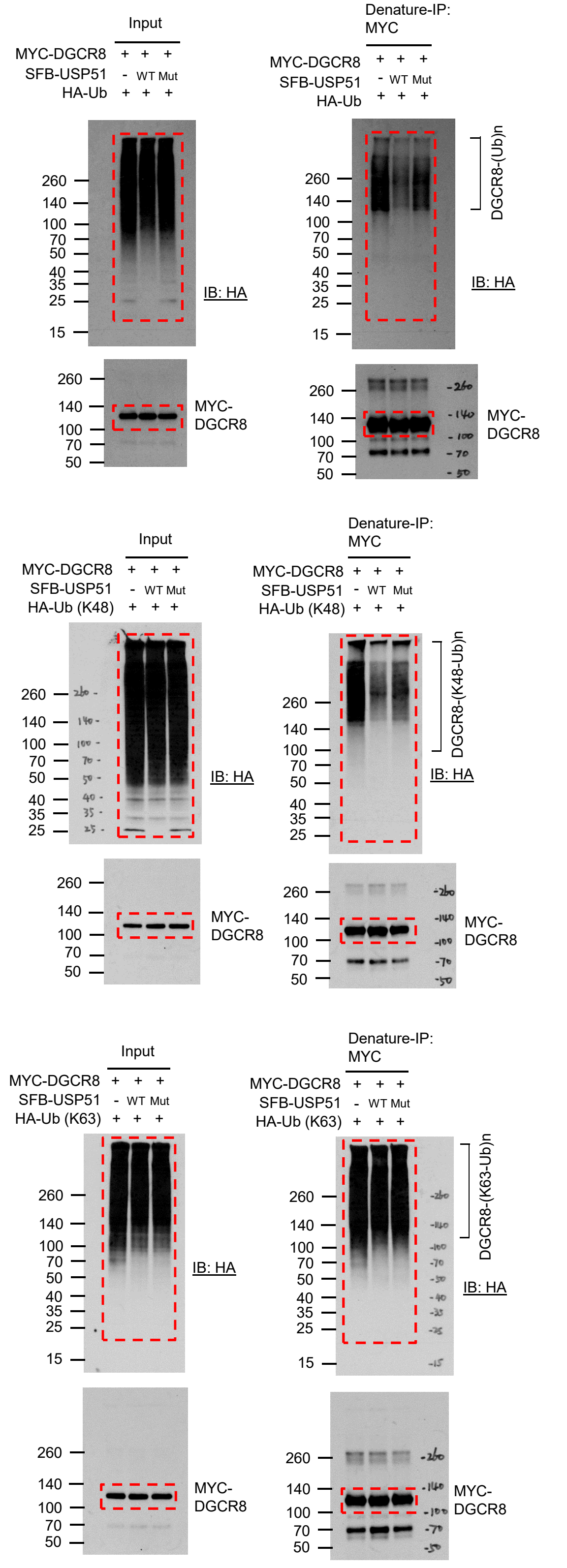

Supplementary Figure 12. All uncropped blots and gels in this manuscript.

Supplementary Figure 4j

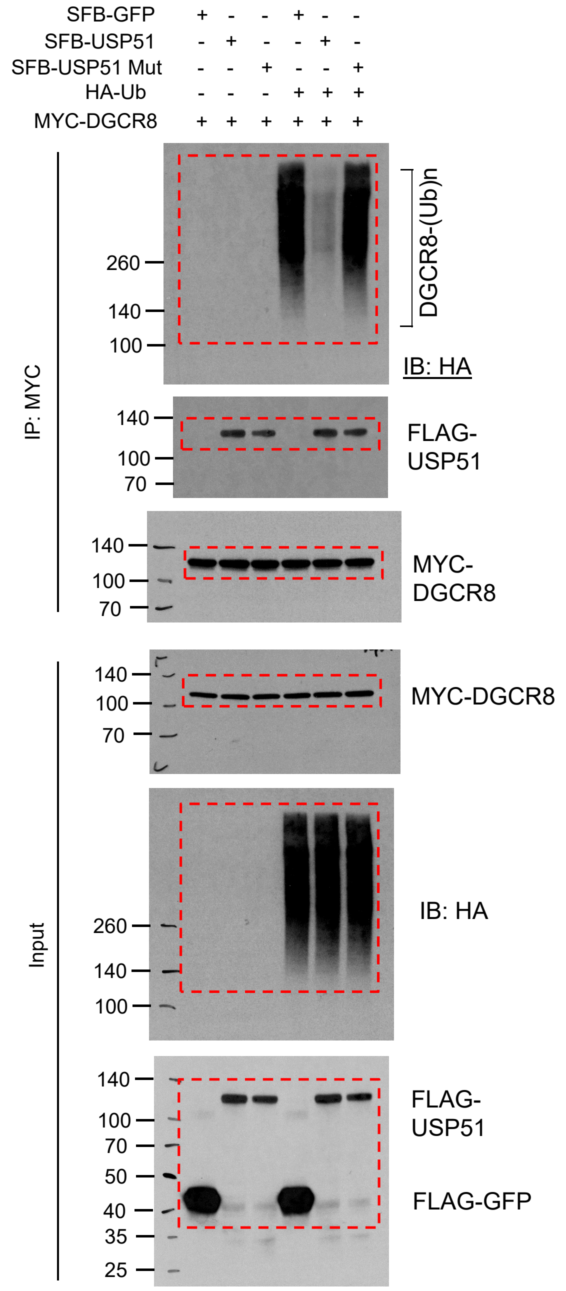

Supplementary Figure 5a

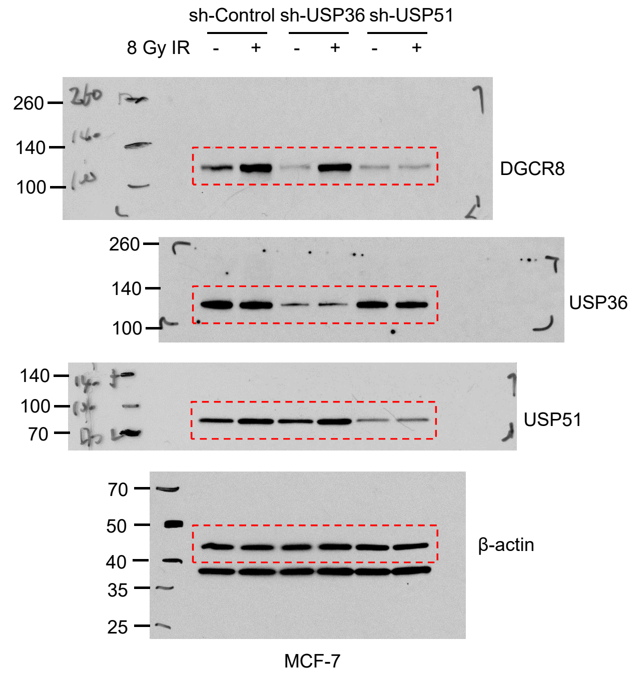

Supplementary Figure 5b

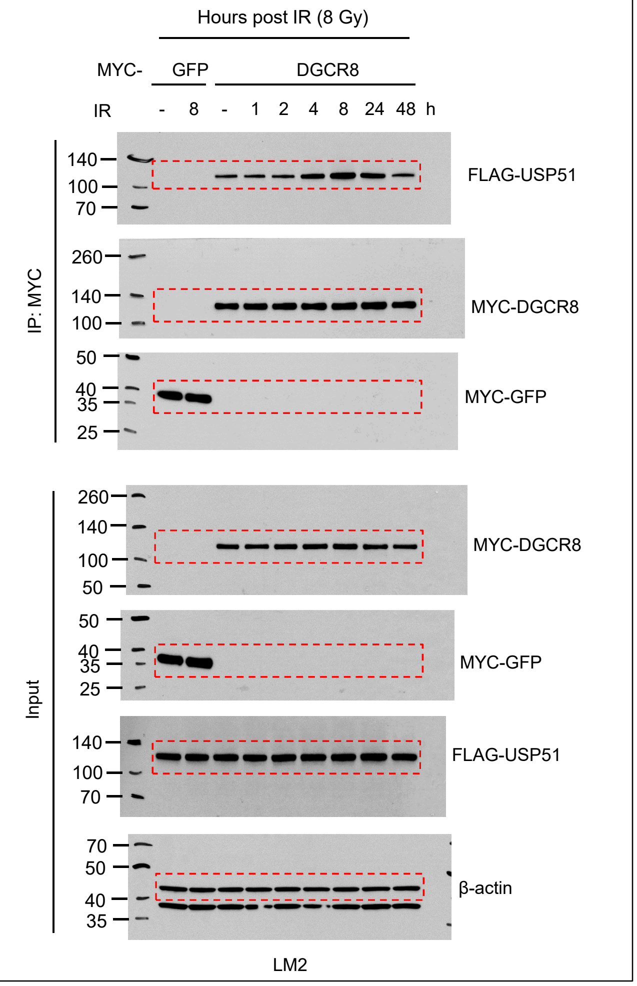

Supplementary Figure 5c

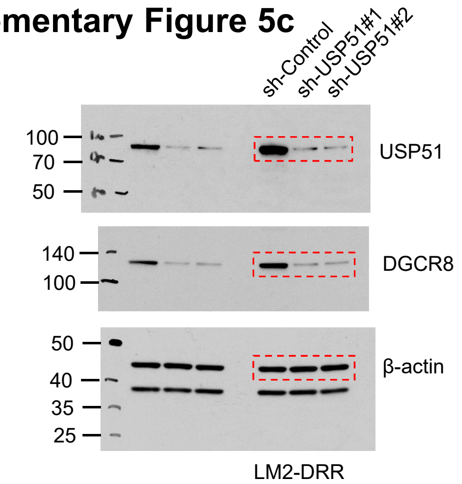

Supplementary Figure 12. All uncropped blots and gels in this manuscript.

Supplementary Figure 12

Supplementary Figure 8a

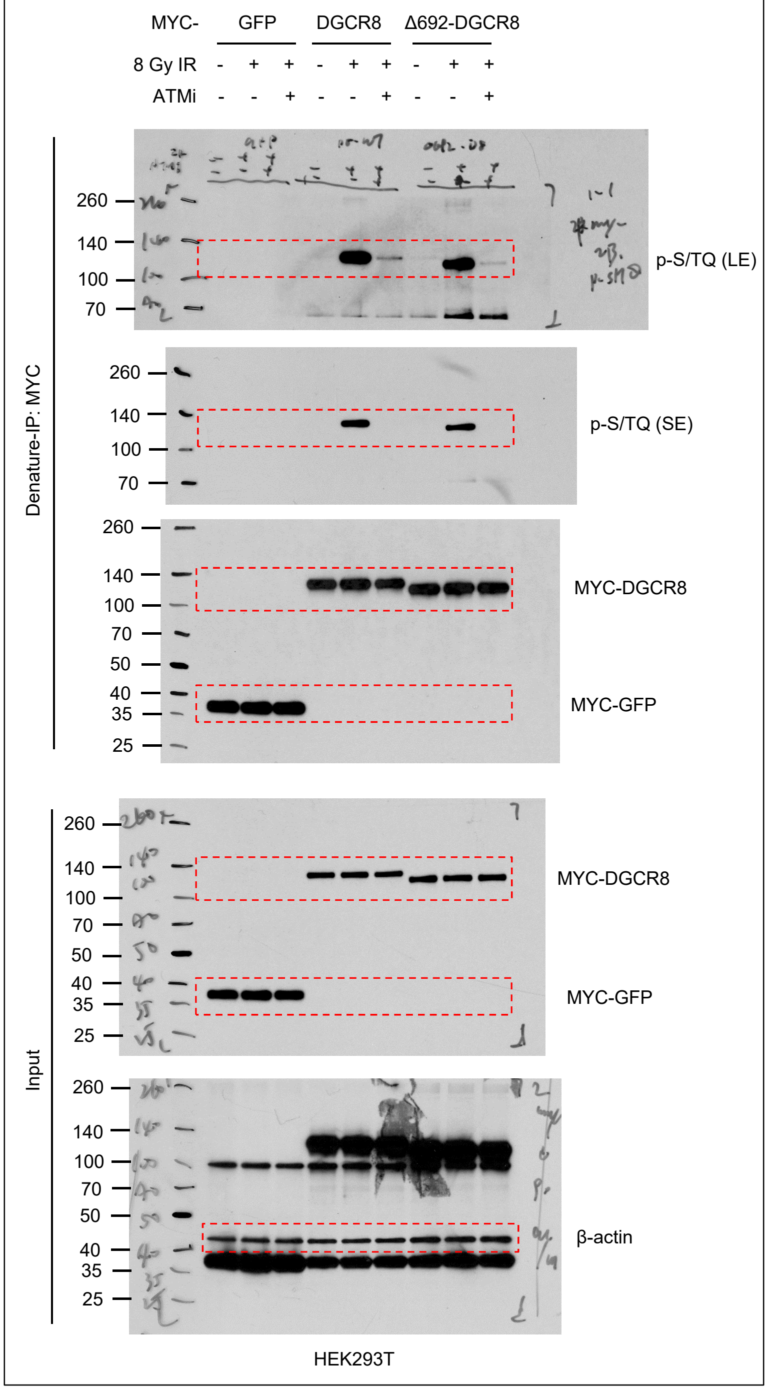

Supplementary Figure 8c

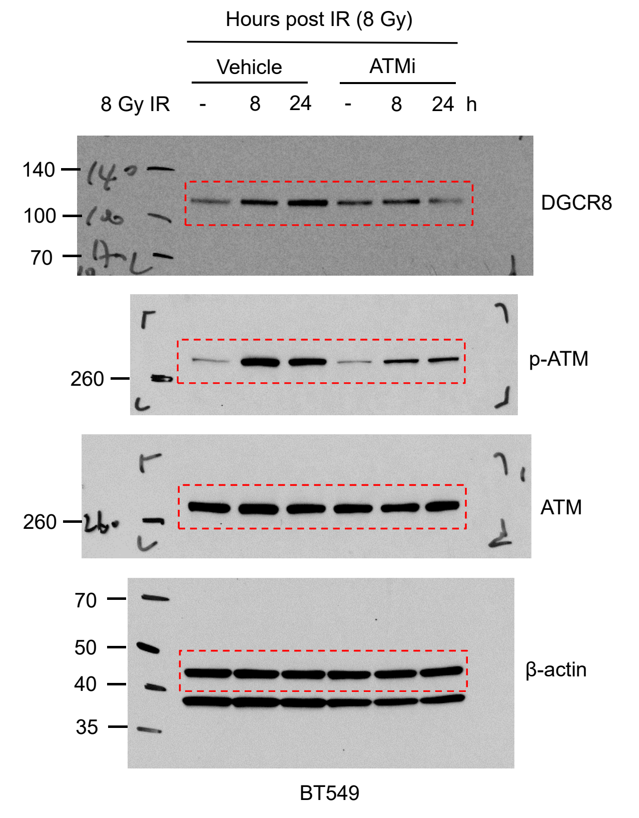

Supplementary Figure 8d

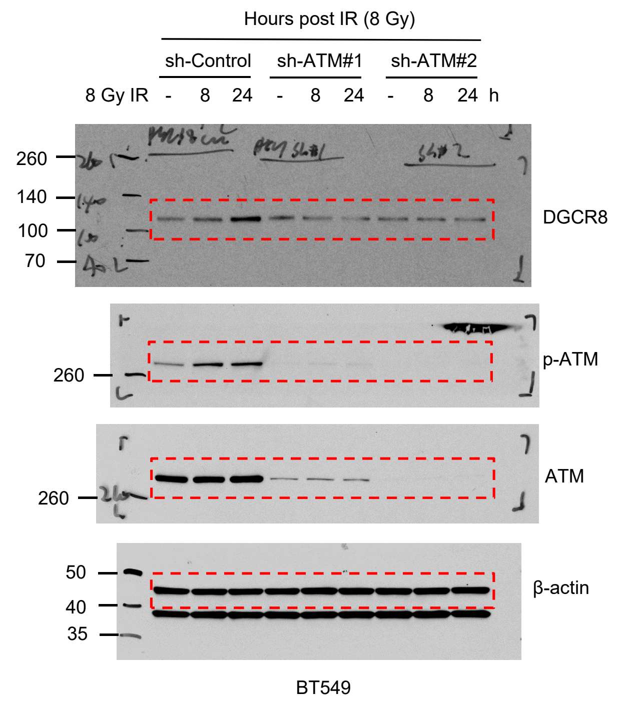

Supplementary Figure 12. All uncropped blots and gels in this manuscript.

Supplementary Figure 12

Supplementary Figure 8e

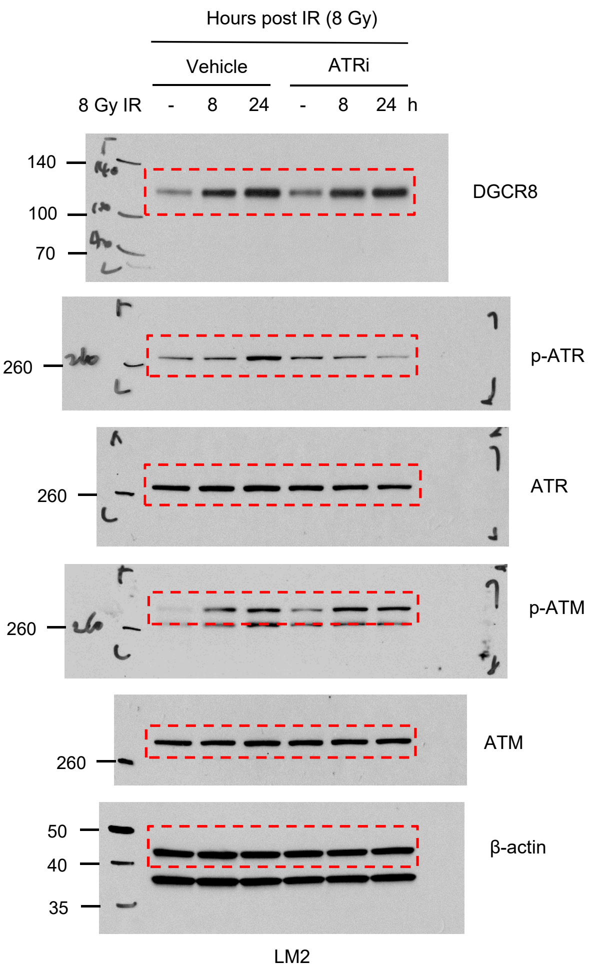

Supplementary Figure 8f

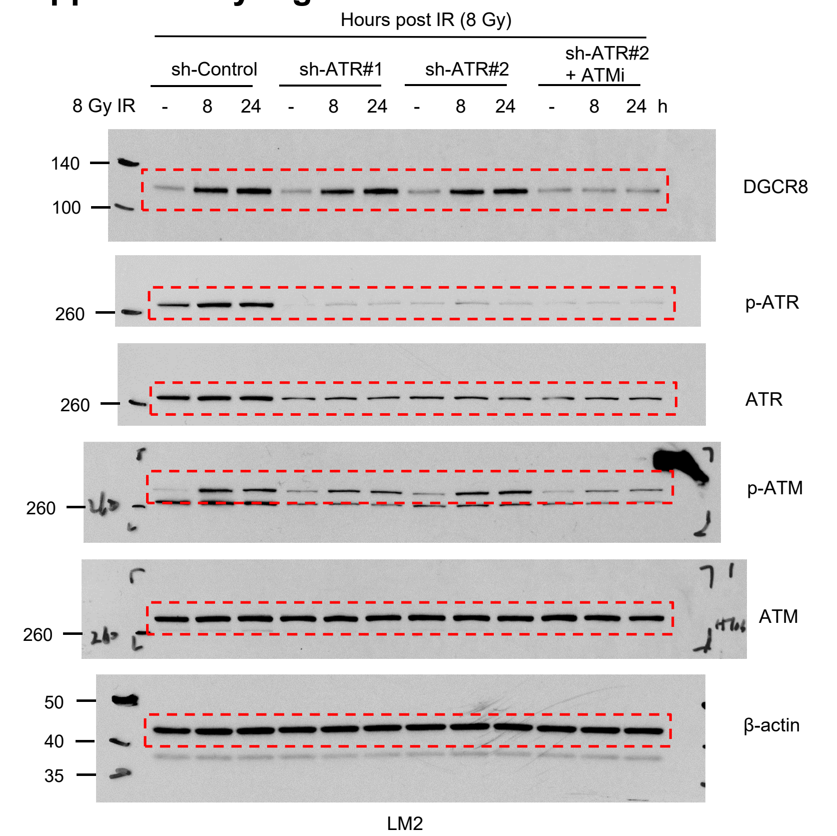

Supplementary Figure 8g

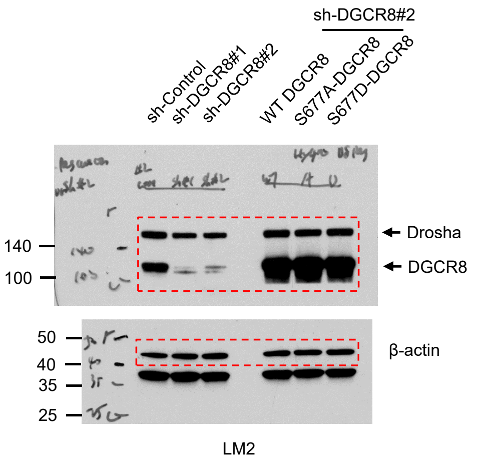

Supplementary Figure 8h

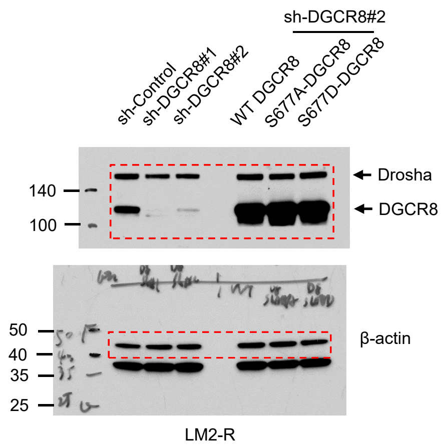

Supplementary Figure 8i

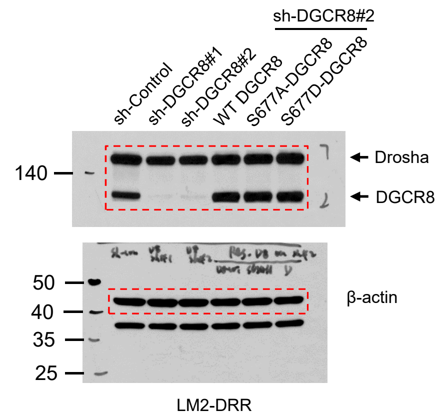

Supplementary Figure 8l

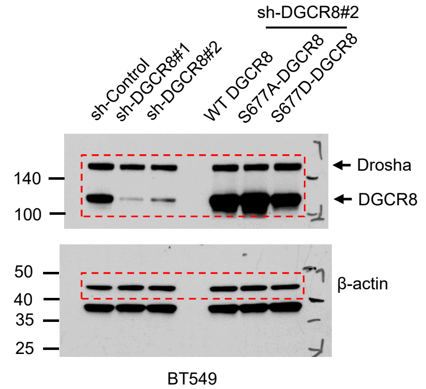

Supplementary Figure 12. All uncropped blots and gels in this manuscript.

Supplementary Table 1. Primers for qPCR

Target gene	Primer sequence (5′-3′)
DGCR8-forward	GCAAGATGCACCCACAAAGA
DGCR8-reverse	TTGAGGACACGCTGCATGTAC
GAPDH-forward	ACATCGCTCAGACACCATG
GAPDH-reverse	TGTAGTTGAGGTCAATGAAGGG
