# Supplementary material for: Non-canonical function of DGCR8 in DNA double-strand break repair signaling and tumor radioresistance
Source: Nat Commun. 2021 Jun 29;12:4033. doi: 10.1038/s41467-021-24298-z (PMC8242032; doi:10.1038/s41467-021-24298-z)
Supplement: Supplementary file 2 — Reporting Summary [file 41467_2021_24298_MOESM2_ESM.pdf]

nature research

Corresponding author(s): Li Ma

Last updated by author(s): Jun 2, 2021

Reporting Summary

Nature Research wishes to improve the reproducibility of the work that we publish. This form provides structure for consistency and transparency in reporting. For further information on Nature Research policies, see our Editorial Policies and the Editorial Policy Checklist.

Statistics

For all statistical analyses, confirm that the following items are present in the figure legend, table legend, main text, or Methods section.

n/a	Confirmed	
☐	☒	The exact sample size (n) for each experimental group/condition, given as a discrete number and unit of measurement
☐	☒	A statement on whether measurements were taken from distinct samples or whether the same sample was measured repeatedly
☐	☒	The statistical test(s) used AND whether they are one- or two-sided<br>*Only common tests should be described solely by name; describe more complex techniques in the Methods section.*
☒	☐	A description of all covariates tested
☒	☐	A description of any assumptions or corrections, such as tests of normality and adjustment for multiple comparisons
☐	☒	A full description of the statistical parameters including central tendency (e.g. means) or other basic estimates (e.g. regression coefficient) AND variation (e.g. standard deviation) or associated estimates of uncertainty (e.g. confidence intervals)
☐	☒	For null hypothesis testing, the test statistic (e.g. F, t, r) with confidence intervals, effect sizes, degrees of freedom and P value noted<br>*Give P values as exact values whenever suitable.*
☒	☐	For Bayesian analysis, information on the choice of priors and Markov chain Monte Carlo settings
☒	☐	For hierarchical and complex designs, identification of the appropriate level for tests and full reporting of outcomes
☐	☒	Estimates of effect sizes (e.g. Cohen's d, Pearson's r), indicating how they were calculated

Our web collection on statistics for biologists contains articles on many of the points above.

Software and code

Policy information about availability of computer code

Data collection	Attune™ NxT software (version H.0) was used to collect flow cytometry data. The LC-MS complete platform that consists of the easy-nLC 1200 chromatography system and the Orbitrap HF-X mass spectrometer, controlled by the Proteome Discoverer software (version 2.2, Thermo Fisher Scientific), was used to collect data for DGCR8-interacting proteins and DGCR8 phosphorylation sites.
Data analysis	ImageJ (version 1.53g) was used for densitometric analysis of Western blots. Zen 2.6 (Zeiss) software was used in confocal image processing. Graphpad Prism 8.0 was used for graphs and statistical analysis. SPSS 25.0 was used for Kaplan-Meier curves and statistical analysis. Attune™ NxT software (version H.0) was used for flow cytometry data analysis. MS raw files of the DGCR8-interacting proteins were searched against the human proteome database from UniProt (January 26, 2019, updated, 93,798 sequences) using MaxQuant software (version 1.6.7.0). Mascot v.2.6.01 was used for DGCR8 phosphorylation site analysis. Survival analysis in Figure 1l was performed by using the Kaplan-Meier Plotter (https://kmplot.com/analysis/index.php?p=service&cancer=breast, dataset: GSE2034). All software and algorithms for data analyses used in this study are well-established from previous work. All software and custom descriptions are included in the Methods section. There is no unreported algorithm used in this manuscript.

For manuscripts utilizing custom algorithms or software that are central to the research but not yet described in published literature, software must be made available to editors and reviewers. We strongly encourage code deposition in a community repository (e.g. GitHub). See the Nature Research guidelines for submitting code & software for further information.

Data

Policy information about availability of data

All manuscripts must include a data availability statement. This statement should provide the following information, where applicable:

- Accession codes, unique identifiers, or web links for publicly available datasets
- A list of figures that have associated raw data
- A description of any restrictions on data availability

The raw data and processed data for mass spectrometric analysis of DGCR8-interacting proteins and DGCR8 phosphorylation sites have been deposited to the MassIVE database with the identifier MSV000087301. UniProt is a free public resource of protein sequence and functional information (https://www.uniprot.org/). The uncropped gels and blots are shown in Supplementary Fig. 12. Source data are provided with this paper.

Field-specific reporting

Please select the one below that is the best fit for your research. If you are not sure, read the appropriate sections before making your selection.

☒ Life sciences ☐ Behavioural & social sciences ☐ Ecological, evolutionary & environmental sciences

For a reference copy of the document with all sections, see nature.com/documents/nr-reporting-summary-flat.pdf

Life sciences study design

All studies must disclose on these points even when the disclosure is negative.

Sample size	No sample size calculation was performed. All experiments were performed using sample sizes based on standard protocols in the field and our previous studies (Zhang, Nature Cell Biology 2014, PMID: 25086746; Kim, Nature Genetics 2018, PMID: 30349115). We have made efforts to avoid needless use of animals. Unless otherwise noted, n = 3 independent biological replicates were used.
Data exclusions	Mice were excluded if euthanasia had to be performed due to sickness or ulcerations prior to the end point of the experiment. No other data were excluded.
Replication	Each experiment was independently repeated three times or more, except for the animal study (one time), DUB screening (one time), tissue microarray and immunohistochemical analysis (one time), and mass spectrometric analysis (two times); the representative data are shown. All replication attempts were successful.
Randomization	Mice were randomly assigned to different treatment groups.
Blinding	For cell-based experiments, Western blotting, immunofluorescence, flow cytometry, and in vitro assays, blinding was not performed, because the investigator had to know the groups to load the samples or perform the assay. For animal experiments, an investigator (L.W.) who measured tumor size was blinded to the group allocation during animal experiments and outcome assessment. For mass spectrometric analysis, an investigator (L.N.) was blinded to the group allocation during data collection and analysis.

Reporting for specific materials, systems and methods

We require information from authors about some types of materials, experimental systems and methods used in many studies. Here, indicate whether each material, system or method listed is relevant to your study. If you are not sure if a list item applies to your research, read the appropriate section before selecting a response.

Materials & experimental systems

n/a	Involved in the study	
☐	☒	Antibodies
☐	☒	Eukaryotic cell lines
☒	☐	Palaeontology and archaeology
☐	☒	Animals and other organisms
☒	☐	Human research participants
☒	☐	Clinical data
☒	☐	Dual use research of concern

Methods

n/a	Involved in the study	
☒	☐	ChIP-seq
☐	☒	Flow cytometry
☒	☐	MRI-based neuroimaging

Antibodies

Antibodies used	Immunoblotting:<br>Antibodies against DGCR8 (1:2,000, Abcam, #ab191875), Dicer (1:1,000, Cell Signaling Technology, #5362S), Drosha (1:1,000, Cell Signaling Technology, #3364S), Exportin-5 (1:1,000, Cell Signaling Technology, #12565), γH2AX (1:1,000, Cell Signaling Technology, #9718S), H2AX (1:1,000, Cell Signaling Technology, #2595S), H2A (1:1,000, Cell Signaling Technology, #2578S), p-CHK1 (1:1,000, Cell

Signaling Technology, #12302S), CHK1 (1:1,000, Cell Signaling Technology, #2360S), p-CHK2 (1:1,000, Cell Signaling Technology, #2661S), CHK2 (1:1,000, Cell Signaling Technology, #6334S), p-ATM (1:1,000, Cell Signaling Technology, #5883S), ATM (1:1,000, Cell Signaling Technology, #2873S), p-ATR (1:1,000, Cell Signaling Technology, #2853S), ATR (1:1,000, Cell Signaling Technology, #2790S), p-S/TQ (1:1,000, Cell Signaling Technology, 2851S), MBP (1:1,000, Cell Signaling Technology, 2396S), GST (1:1,000, Cell Signaling Technology, #2622S), MDC1 (1:1000, R&D Systems, #MAB6497), RNF8 (1:1,000, Millipore, #09-813), RNF168 (1:1,000, Millipore, #ABE367), USP36 (1:1,000, a gift from Dr. Masayuki Komada at Tokyo Institute of Technology), USP51 (1:3,000, a gift from Dr. Sharon Dent at MD Anderson Cancer Center), β-actin (1:1,000, Santa Cruz Biotechnology, #sc-47778), FLAG (1:5,000, Sigma, #F3165, clone M2), HA (1:2,000, Santa Cruz Biotechnology, #sc-7392), and MYC (1:2,000, Santa Cruz Biotechnology, #sc-40, clone 9E10). The antibody against phospho-DGCR8 (pS677; 1:500) was generated at Biomatik.

Immunoprecipitation and pulldown assays:
AntibodIes against RNF8 (Proteintech, #14112-1-AP), RNF168 (Proteintech, #21393-1-AP), and DGCR8 (Bethyl Laboratories, #A302-468A).

Immunofluorescence:
Antibodies against γH2AX (1:100, Cell Signaling Technology, #9718S), γH2AX (1:100, BD Biosciences, #560443), DGCR8 (1:100, Abcam, #ab191875), MDC1 (1:200, Bio-Rad, #AHP799), RNF8 (1:200, Proteintech, #14112-1-AP), RNF168 (1:100, Millipore, #ABE367), BRCA1 (1:20, Santa Cruz Biotechnology, #sc-6954), 53BP1 (1:100, Novus Biologicals, #NB100-304), FLAG (1:500, Sigma, #F7425), MYC (1:500, Santa Cruz Biotechnology, #sc-40, clone 9E10), Alexa Fluor 488 goat anti-rabbit IgG (1:1,000, Invitrogen, ThermoFisher Scientific, #A-11008), Alexa Fluor 647 donkey anti-sheep IgG (1:1,000, Invitrogen, ThermoFisher Scientific, #A-21448), Alexa Fluor 488 goat anti-mouse IgG (1:1,000, Invitrogen, ThermoFisher Scientific, #A-11001), Alexa Fluor 594 goat anti-rabbit IgG (1:1,000, Invitrogen, ThermoFisher Scientific, #A-11012), and Alexa Fluor 594 goat anti-mouse IgG (1:1,000, Invitrogen, ThermoFisher Scientific, #A-11005).

Immunohistochemistry:
Antibodies against DGCR8 (1:250, Abcam, #ab90579) and USP51 (1:250, Abcam, #ab121147).

Validation

All antibodies used in this study have been validated, and detailed information can be found on the website from the manufacturer or related publications listed below. Some of them have also been validated by experiments shown in this manuscript under overexpression or knockdown settings. The experiments included appropriate controls. The phopho-DGCR8 (S677) antibody was validated by comparing wild-type DGCR8 and the S677A mutant of DGCR8.

DGCR8, https://www.abcam.com/dgcr8-antibody-epr18757-ab191875.html
DGCR8, https://www.bethyl.com/product/A302-468A/DGCR8+Antibody
phospho-S677 DGCR8, https://www.biomatik.com/services/custom-antibody-services/polyclonal-antibody-production.html
Dicer, https://www.cellsignal.com/products/primary-antibodies/dicer-d38e7-rabbit-mab/5362
Drosha, https://www.cellsignal.com/products/primary-antibodies/drosha-d28b1-rabbit-mab/3364
Exportin-5, https://www.cellsignal.com/products/primary-antibodies/exportin-5-d7w6w-rabbit-mab/12565
γH2AX, https://www.cellsignal.com/products/primary-antibodies/phospho-histone-h2a-x-ser139-20e3-rabbit-mab/9718
γH2AX, https://www.bdbiosciences.com/us/applications/research/apoptosis/purified-antibodies/purified-mouse-anti-h2ax-ps139-n1-431/p/560443
H2AX, https://www.cellsignal.com/products/primary-antibodies/histone-h2a-x-antibody/2595
H2A, https://www.cellsignal.com/products/primary-antibodies/histone-h2a-antibody-ii/2578
p-CHK1, https://www.cellsignal.com/products/primary-antibodies/phospho-chk1-ser317-d12h3-xp-rabbit-mab/12302
CHK1, https://www.cellsignal.com/products/primary-antibodies/chk1-2g1d5-mouse-mab/2360
p-CHK2, https://www.cellsignal.com/products/primary-antibodies/phospho-chk2-thr68-antibody/2661
CHK2, https://www.cellsignal.com/products/primary-antibodies/chk2-d9c6-xp-rabbit-mab/6334
p-ATM, https://www.cellsignal.com/products/primary-antibodies/phospho-atm-ser1981-d6h9-rabbit-mab/5883
ATM, https://www.cellsignal.com/products/primary-antibodies/atm-d2e2-rabbit-mab/2873
p-ATR, https://www.cellsignal.com/products/primary-antibodies/phospho-atr-ser428-antibody/2853
ATR, https://www.cellsignal.com/products/primary-antibodies/atr-antibody/2790
p-S/TQ, https://www.cellsignal.com/products/primary-antibodies/phospho-ser-thr-atm-atr-substrate-antibody/2851
MBP, https://www.cellsignal.com/products/primary-antibodies/mbp-tag-8g1-mouse-mab/2396
GST, https://www.cellsignal.com/products/primary-antibodies/gst-antibody/2622
MDC1, https://www.rndsystems.com/products/human-mdc1-antibody-398636_mab6497#product-details
MDC1, https://www.bio-rad-antibodies.com/static/datasheets/ahp79/human-mdc1-antibody-ahp799.pdf
RNF8, https://www.emdmillipore.com/US/en/product/Anti-RING-finger-protein-8-Antibody,MM_NF-09-813
RNF8, https://www.ptglab.com/products/RNF8-Antibody-14112-1-AP.htm
RNF168, https://www.emdmillipore.com/US/en/product/Anti-RNF168-Antibody,MM_NF-ABE367
RNF168, https://www.ptglab.com/products/RNF168-Antibody-21393-1-AP.htm
53BP1, https://www.novusbio.com/products/53bp1-antibody_nb100-304
BRCA1, https://www.scbt.com/p/brca1-antibody-d-9
USP36, https://jcs.biologists.org/content/122/5/678
USP51, https://www.sciencedirect.com/science/article/pii/S1097276516300132?via%3Dihub
β-actin, https://www.scbt.com/p/beta-actin-antibody-c4
FLAG, https://www.sigmaaldrich.com/catalog/product/sigma/f1804?lang=en&region=US&gclid=Cj0KCQjwoPL2BRDxARIsAEMm9y_7mueSXyGGIJ93O38wK_zOTdpEReC8h9UpfhgJBOzdS5ZoCG2os2gaAhGZEALw_wcB
HA, https://www.scbt.com/p/ha-probe-antibody-f-7
MYC, https://www.scbt.com/p/c-myc-antibody-9e10
DGCR8, https://www.abcam.com/dgcr8-antibody-ab90579.html

USP51, https://www.abcam.com/usp51-antibody-ab121147.html
Alexa Fluor 488 goat anti-rabbit IgG, https://www.thermofisher.com/antibody/product/Goat-anti-Rabbit-IgG-H-L-Cross-Adsorbed-Secondary-Antibody-Polyclonal/A-11008
Alexa Fluor 647 donkey anti-sheep IgG, https://www.thermofisher.com/antibody/product/Donkey-anti-Sheep-IgG-H-L-Cross-Adsorbed-Secondary-Antibody-Polyclonal/A-21448
Alexa Fluor 488 goat anti-mouse IgG, https://www.thermofisher.com/antibody/product/Goat-anti-Mouse-IgG-H-L-Cross-Adsorbed-Secondary-Antibody-Polyclonal/A-11001
Alexa Fluor 594 goat anti-rabbit IgG, https://www.thermofisher.com/antibody/product/Goat-anti-Rabbit-IgG-H-L-Cross-Adsorbed-Secondary-Antibody-Polyclonal/A-11012
Alexa Fluor 594 goat anti-mouse IgG, https://www.thermofisher.com/antibody/product/Goat-anti-Mouse-IgG-H-L-Cross-Adsorbed-Secondary-Antibody-Polyclonal/A-11005

Eukaryotic cell lines

Policy information about cell lines

Cell line source(s)	The HEK293T, T47D, MCF-7, BT549, HepG2, HCT116, and HeLa cell lines were from the American Type Culture Collection (ATCC). The LM2 cell line was from Xiang Zhang (Baylor College of Medicine) and the HEK293A cell line was from Junjie Chen's lab stock.
Authentication	Short tandem repeat (STR) profiling was done by ATCC and MD Anderson's Characterized Cell Line Core Facility.
Mycoplasma contamination	All cell lines tested negative for mycoplasma contamination.
Commonly misidentified lines (See ICLAC register)	No cell lines used in this study are in the database of commonly misidentified cell lines.

Animals and other organisms

Policy information about studies involving animals; ARRIVE guidelines recommended for reporting animal research

Laboratory animals	Solitary tumor xenografts were produced in the muscle of the right hind limb of 10-week-old female nude mice (from MD Anderson Cancer Center's internal supply). Mice were housed at 70F-74F (set point: 72F) with 40%-55% humidity (set point: 45%). The light cycle of animal rooms is 12 h of light and 12 h of dark.
Wild animals	The study did not involve wild animals.
Field-collected samples	The study did not involve samples collected from the field.
Ethics oversight	Animal experiments were performed in accordance with a protocol approved by the Institutional Animal Care and Use Committee of MD Anderson Cancer Center, and mice were euthanized when they met the institutional euthanasia criteria for tumor size or overall health condition.

Note that full information on the approval of the study protocol must also be provided in the manuscript.

Flow Cytometry

Plots

Confirm that:

- [x] The axis labels state the marker and fluorochrome used (e.g. CD4-FITC).
- [x] The axis scales are clearly visible. Include numbers along axes only for bottom left plot of group (a 'group' is an analysis of identical markers).
- [x] All plots are contour plots with outliers or pseudocolor plots.
- [x] A numerical value for number of cells or percentage (with statistics) is provided.

Methodology

Sample preparation	Indiacated cells cultured in 10-well plates were co-transfected with pCBA-I-SceI and pCAGGS DRR mCherry Donor EF1a BFP. The cells were trypsinized, washed once with PBS, resuspended in cold PBS, and subjected to flow cytometric analysis using Attune NxT.
Instrument	Attune NxT Flow Cytometer (Invitrogen, Thermo Fisher Scientific)
Software	We used Attune NxT software vH.0 to collect and analyze data.
Cell population abundance	At least 150,000 cells were analyzed for each sample.

Gating strategy

Initial cell population gating (SSC-Area vs FSC-Area) was adopted to ensure that only single cells were used for analysis. The same cell gating strategy was applied to all samples analyzed at the same time.

☒ Tick this box to confirm that a figure exemplifying the gating strategy is provided in the Supplementary Information.
